# A Tailored COF for Visible-Light Photosynthesis of 2,3-Dihydrobenzofurans

**DOI:** 10.1021/jacs.2c10471

**Published:** 2023-02-24

**Authors:** Prakash T. Parvatkar, Sharath Kandambeth, Aslam C. Shaikh, Issatay Nadinov, Jun Yin, Vinayak S. Kale, George Healing, Abdul-Hamid Emwas, Osama Shekhah, Husam N. Alshareef, Omar F. Mohammed, Mohamed Eddaoudi

**Affiliations:** † Functional Materials Design, Discovery and Development Research Group (FMD3), Advanced Membranes and Porous Materials Center (AMPM), Division of Physical Science and Engineering (PSE), King Abdullah University of Science and Technology (KAUST), Thuwal 23955-6900, Kingdom of Saudi Arabia; ‡ Advanced Membranes and Porous Materials Center (AMPM), Division of Physical Science and Engineering (PSE), King Abdullah University of Science and Technology (KAUST), Thuwal 23955-6900, Kingdom of Saudi Arabia; § Department of Applied Physics, The Hong Kong Polytechnic University, Hung Hom, Kowloon, 999077 Hong Kong People’s Republic of China; ∥ Core Laboratories, King Abdullah University of Science and Technology (KAUST), Thuwal 23955-6900, Kingdom of Saudi Arabia; ⊥ Division of Physical Science and Engineering (PSE), King Abdullah University of Science and Technology (KAUST), Thuwal 23955-6900, Kingdom of Saudi Arabia

## Abstract

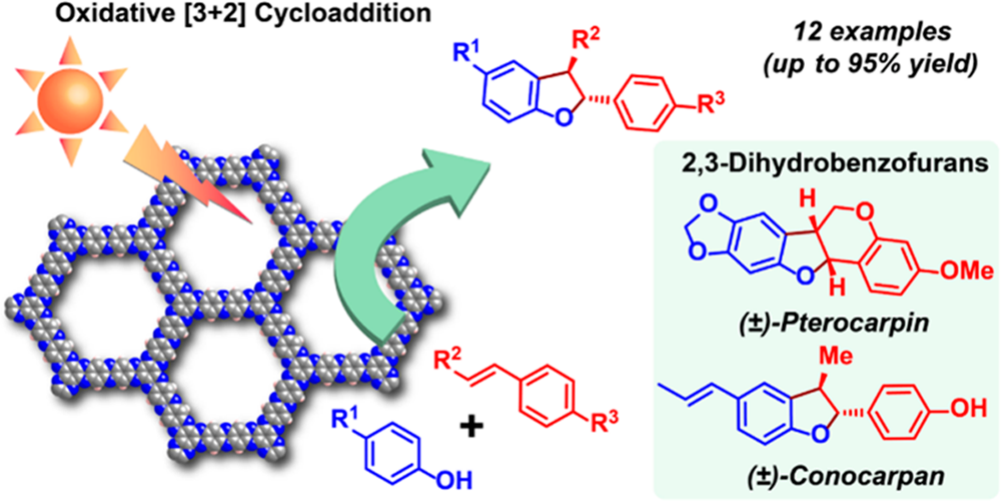

Heterogeneous photocatalysis is considered as an ecofriendly and sustainable approach for addressing energy and environmental persisting issues. Recently, heterogeneous photocatalysts based on covalent organic frameworks (COFs) have gained considerable attention due to their remarkable performance and recyclability in photocatalytic organic transformations, offering a prospective alternative to homogeneous photocatalysts based on precious metal/organic dyes. Herein, we report **Hex-Aza-COF-3** as a metal-free, visible-light-activated, and reusable heterogeneous photocatalyst for the synthesis of 2,3-dihydrobenzofurans, as a pharmaceutically relevant structural motif, via the selective oxidative [3+2] cycloaddition of phenols with olefins. Moreover, we demonstrate the synthesis of natural products (±)-conocarpan and (±)-pterocarpin via the [3+2] cycloaddition reaction as an important step using **Hex-Aza-COF-3** as a heterogeneous photocatalyst. Interestingly, the presence of phenazine and hexaazatriphenylene as rigid heterocyclic units in **Hex-Aza-COF-3** strengthens the covalent linkages, enhances the absorption in the visible region, and narrows the energy band, leading to excellent activity, charge transport, stability, and recyclability in photocatalytic reactions, as evident from theoretical calculations and real-time information on ultrafast spectroscopic measurements.

## Introduction

Visible-light-mediated organophotocatalysts are gaining considerable interest in synthetic organic chemistry owing to their energy efficiency and green nature.^1a−1c^ Although metal-based catalysts^2a−2d^ still dominate the photocatalysis field, the development of efficient metal-free photocatalysts^3a−3c^ is highly desirable due to their plausible nontoxicity and low cost. Despite their successful application in various organic transformations, homogeneous organo-dye-based photocatalysts suffer from limited stability and recyclability, requiring immobilization in heterogeneous matrices for enhancing their stability. One-dimensional (1D) polymers have been tested as an organo-heterogeneous support for the immobilization of organo-dye-based catalysts.^4a,4b^ However, 1D polymers offer limited exposure of the catalytic sites due to their low porosity, leading to poor conversion efficiency.^5^ Therefore, the quest for novel porous organo-heterogeneous matrices that offer efficient immobilization of organocatalysts while preserving the catalytic activity is of tremendous interest.

In this context, covalent organic frameworks (COFs), an emerging class of crystalline porous organic materials, offer great potential as tunable heterogeneous organocatalysts for multiple organic transformations.^6a−6c^ Their tailor-made structure, functional diversity, high surface area, insoluble nature, and high thermal stability make them an excellent candidate for catalytic applications. Furthermore, COFs are synthesized via simple organic condensation reactions that do not require any metal-based catalysts. Therefore, unlike porous organic polymers (POPs), the prospect of noble metal impurities’ interference with COF-based organo catalysis may be entirely precluded.^7a^

In recent years, two-dimensional (2D) COFs have been investigated as photocatalysts^7a−7d^ because of their predesignable open-framework structure and interesting optoelectronic properties. The optimal arrangement of periodic donor–acceptor π-columnar arrays in 2D COFs offers preorganized pathways for charge carriers and prevents the fast recombination of photogenerated charge carriers, affording enhanced photocatalytic conversion efficiency. Despite these interesting features, 2D COFs have only been applied as heterogeneous photocatalysts in fundamental organic transformations such as cross-coupling reactions,^8a−8e^ olefin isomerization,^9^ oxidation of alcohols,^10a,10b^ sulfides,^11a−11c^ amines,^12a−12c^ and boronic acids,^13a−13c^ reductive dehalogenation,^14a,14b^ and heterocyclization,^15a−15c^ whereas their application as visible-light-activated heterogeneous photocatalysts in complex organic transformations has rarely been explored.

The scarcity of highly active COF-based photocatalysts can be attributed to the low photo- and chemical stability of COFs and the limited availability of highly active organophotocatalytic cores. Most of the reported COF photocatalysts are based on acid- or base-sensitive linkages such as imines or boronate ester-based linkages, which limit their recyclability and photocatalytic activities. Moreover, to realize practical applications, a systematic investigation to understand the arrangement of active photocatalytic units in COFs is still required. In addition to enhancing the stability of COFs, achieving an efficient separation of photogenerated carriers is important for the development of effective COF-based photocatalysts. This can be performed by forming a heterojunction and introducing either an active backbone or active side chains. By adopting this approach, multiple COFs having stable linkages such as hydrazone-,^15a,16^ benzoxazole-,^13a^ thieno[3,2-*c*]pyridine-,^17^ triazine-,^8c,9,10a,10b^ and olefin-linked COFs^12a,12b,18^ were reported as photocatalysts for various organic transformations. Despite these advances, additional progress in the development of robust and effective COFs for practical photocatalysis is still urgently required.

Recently, our group reported a highly stable redox-functionalized COF, **Hex-Aza-COF-3**, as an electrode for high-performance asymmetric supercapacitors^19^ and zinc-ion supercapatteries.^20^ The completely aromatized and conjugated imine bonds in **Hex-Aza-COF-3** offer enhanced photo- and chemical stability compared to the reported imine-based COF photocatalysts. The **Hex-Aza-COF-3** framework comprises two units, namely, phenazine and hexaazatriphenylene (HAT), which act as an electron donor^21a−21c^ and an electron acceptor,^22a−22c^ respectively, and are known to endow their compounds with visible-light absorption ability. The in-built electron donor–acceptor units prompted us to explore this COF as a heterogeneous photocatalyst for organic transformations.

Herein, we report **Hex-Aza-COF-3** as an efficient metal-free, white-light-activated heterogeneous photocatalyst for the synthesis of 2,3-dihydrobenzofurans (2,3-DHBs) via the selective oxidative [3+2] cycloaddition of phenols with olefins. The synthesis of 2,3-DHBs^23a−23g^ has received considerable interest due to the ubiquitous presence of this scaffold in many pharmaceuticals and bioactive natural products. Compounds containing 2,3-DHB units display a wide range of bioactivities,^24a−24c^ such as antimalarial, anticancer, anti-inflammatory, antifungal, antibacterial, anti-HIV, antioxidative, and antihypotensive activities. The oxidative [3+2] cycloaddition of phenols with olefins has proven to be a simple and practical approach for constructing, 2,3-DHB units. To the best of our knowledge, neither oxidative [3+2] cycloaddition nor the synthesis of 2,3-DHB units has been explored using COFs as heterogeneous photocatalysts to date. We demonstrate in this study that the phenazine and HAT units strengthen the covalent linkages in **Hex-Aza-COF-3**, resulting in high photocatalytic efficiency and unprecedented recyclability after several photocycles. Furthermore, control experiments, density functional theory (DFT) calculations, and transient absorption (TA) spectroscopic studies revealed that an efficient light-induced charge transfer process is responsible for the high photocatalytic activity of **Hex-Aza-COF-3**. This contribution may not only increase the design and synthesis of COFs as robust heterogeneous photocatalysts but also shed more light on the photocatalytic mechanism.

## Results and Discussion

**Figure 1:**
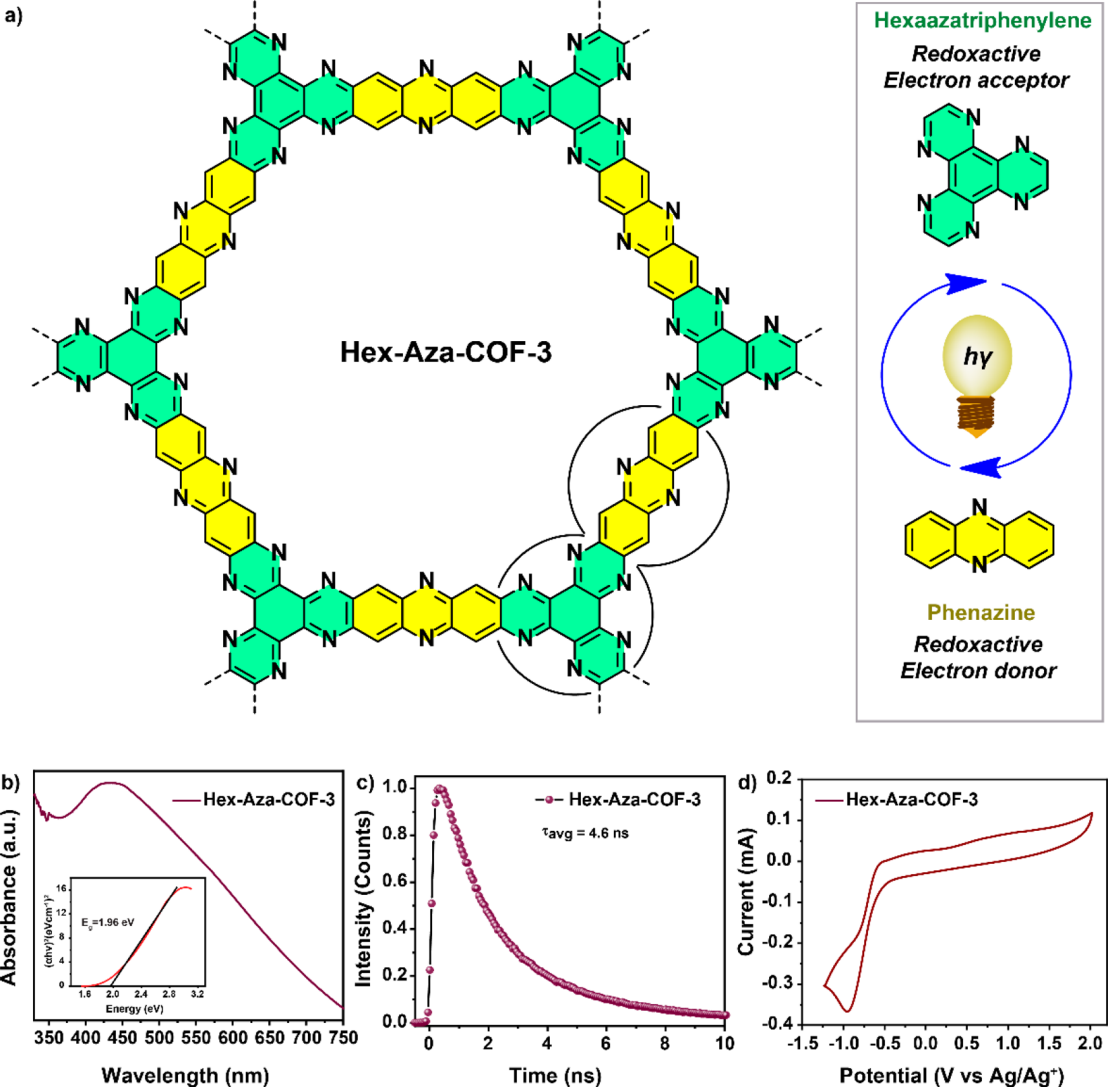
(a) Structure of **Hex-Aza-COF-3**; (b) UV–vis absorption spectrum of **Hex-Aza-COF-3** suspended in dimethylformamide (DMF); (c) photoluminescence decay curve of **Hex-Aza-COF-3** suspended in DMF; (d) cyclic voltammetric analysis of **Hex-Aza-COF-3**.

**Hex-Aza-COF-3** was synthesized via solvothermal condensation reaction between hexaketocyclohexane octahydrate and 2,3,6,7-tetraaminophenazine hydrochloride according to our previously reported procedure^19^ (see SI, Scheme S3) and characterized using powder X-ray diffraction (PXRD), Fourier-transform infrared (FTIR) spectroscopy, and ^13^C cross-polarization magic-angle-spinning (CP-MAS) NMR spectroscopies (see SI, Figure S1).^17^ First, the photophysical and electrochemical properties of **Hex-Aza-COF-3** were investigated using UV–vis absorption, photoluminescence (PL) spectroscopy, and cyclic voltammetry (CV). The UV–vis absorption spectrum (Figure 1b) revealed that **Hex-Aza-COF-3** possesses a strong light absorption over the entire visible-light region from 350 to 750 nm, which is beneficial for photocatalysis. It is to be noted that this broad spectral feature may be attributed to the extensively π-conjugated framework structure. An optical energy gap of 1.96 eV was calculated for **Hex-Aza-COF-3** using the Kubelka–Munk (KM)-transformed reflectance spectrum (Figure 1b inset). The fluorescence lifetime of **Hex-Aza-COF-3** was determined using time-resolved PL spectroscopy. More specifically, the excitation of **Hex-Aza-COF-3** resulted in a strong emission band at λ_em_ = 522 nm with an average lifetime (τ) of 4.6 ± 0.3 ns (Figure 1c). The electrochemical bandgap of **Hex-Aza-COF-3** was determined by performing a CV analysis (Figure 1d) using glassy carbon as a working electrode in anhydrous acetonitrile containing 0.1 M tetrabutylammonium hexafluorophosphate, Ag/AgCl in acetonitrile as a reference electrode, and Pt as a counter electrode with a scan rate of 100 mV s^–1^. The lowest unoccupied molecular orbital (LUMO)–highest occupied molecular orbital (HOMO) separation was reported to be 1.90 eV, which agrees with the optical bandgap of 1.96 eV.

The observed excellent optical and electrochemical properties of **Hex-Aza-COF-3** encouraged us to investigate its performance as a heterogeneous photocatalyst for organic transformations. The oxidative [3+2] cycloaddition of 4-methoxyphenol (**1a**) with *trans*-anethole (**2a**) was selected as a model reaction for our initial optimization studies. Note that this reaction yielded the desired product, 5-methoxy-2-(4-methoxyphenyl)-3-methyl-2,3-dihydrobenzofuran (**3a**), with 93% reaction yield using ammonium persulfate [(NH_4_)_2_S_2_O_8_] as an external oxidant. Although many methods have been reported for synthesizing this type of compound, more practical and environmentally benign methods are still required.

In particular, 4-methoxyphenol and *trans*-anethole were irradiated in acetonitrile using white light-emitting diodes (LEDs) at 25 °C in the presence of a catalytic amount of **Hex-Aza-COF-3** and (NH_4_)_2_S_2_O_8_ as the external oxidant, and the formation of the product was monitored using ^1^H NMR spectroscopy. After 3 h of reaction time, 32% of product formation was observed, which steadily increased with the reaction time until reaching complete conversion after 9 h of irradiation (see SI, Figure S3) with an isolated yield of 93% (Table 1, entry 1). **Hex-Aza-COF-3** showed improved activity compared with the previously reported photocatalyst for this oxidative [3+2] cycloaddition of phenol with olefin, yielding the desired product in high yield, shorter reaction time, and low catalyst loading (see SI, Table S1). To understand the role of the COF catalyst in this transformation, a blank experiment was conducted without **Hex-Aza-COF-3** (Table 1, entry 2). As expected, the peak of the desired product was not observed in the NMR analysis of the reaction crude, which demonstrates that **Hex-Aza-COF-3** is important for this transformation. Similarly, blank experiments were performed in the absence of light (Table 1, entry 3) or the external oxidant (NH_4_)_2_S_2_O_8_ (Table 1, entry 4), in which no product formation was detected. Furthermore, a light-on/light-off experiment was performed over time (see SI, Figure S4), confirming that the reaction proceeded only when the light was on, indicating that the reaction occurs via a photocatalytic pathway. Other oxidants such as *m*-chloroperbenzoic acid (*m*-CPBA), 2,3-dichloro-5,6-dicyano-1,4-benzoquinone (DDQ), di-*tert*-butyl peroxide (DTBP), and air were screened rather than (NH_4_)_2_S_2_O_8_ (Table 1, entries 5–8). No desired product was formed under these conditions (only 6% of the desired product was observed using DDQ as the oxidant), which shows that the selectivity depends on the reduction potential associated with the excited-state COF catalyst. Moreover, reducing the catalyst loading to 1 mol % (Table 1, entry 9) afforded a decrease in the yield of the product compared with the result obtained using 2.5 mol % catalyst (Table 1, entry 1). Furthermore, no product was obtained when using 10 mol % of phenazine derivative **4** (Table 1, entry 10) or HAT derivative **5** (Table 1, entry 11) as the catalyst rather than **Hex-Aza-COF-3**. We calculated the HOMO–LUMO levels of compounds **4** and **5** (see SI, Figure S5) and compared them with those of **Hex-Aza-COF-3**. Calculated bandgaps of **4** and **5** are 3.33 and 3.69 eV respectively, in close agreement with the optical bandgaps (3.50 eV for **4** and 3.55 eV for **5**) and much larger than that of **Hex-Aza-COF-3** (1.77 eV). These results indicate that the photocatalytic activity of **Hex-Aza-COF-3** is not only associated with the phenazine or HAT scaffolds but induced by the highly ordered 2D skeletons, π-conjugated crystalline framework, and porosity of the COF, collectively facilitating the separation of photogenerated electron (e^–^)–hole (h^+^) pairs and the migration of charge carriers.

**Table 1:**
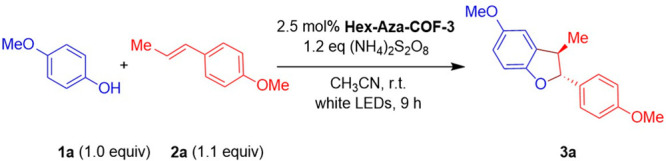

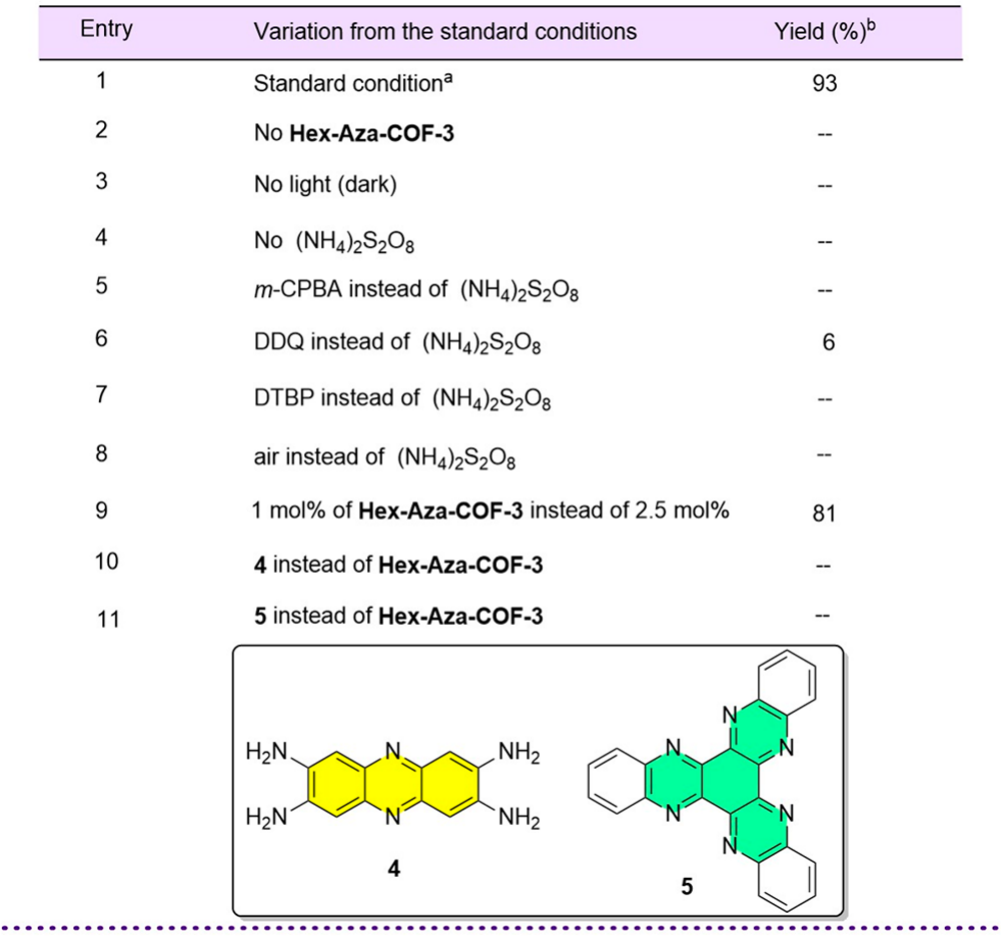
Optimization Studies for the Oxidative [3+2] Cycloaddition of Phenol with Olefin Photocatalyzed by Hex-Aza-COF-3

a Standard conditions: **1a** (0.2 mmol), **2a** (0.22 mmol), 2.5 mol % **Hex-Aza-COF-3**, 0.24 mmol of (NH_4_)_2_S_2_O_8_, 5 mL of CH_3_CN, white LEDs, room temperature, 9 h.

b Isolated yields.

Based on these optimized conditions, the scope of this photocatalyst for the synthesis of 2,3-DHB analogs was then explored by varying the substituents on the phenol and the olefin substrates. As shown in Table 2, in the presence of a catalytic amount of **Hex-Aza-COF-3**, using diverse phenolic substrates efficiently afforded the desired products in good to excellent yields (**3a**–**3l**, 83–95% yield). The reaction worked well with phenol and naphthol derivatives. Similarly, switching disubstituted olefins to monosubstituted derivatives did not affect the yield of the desired product (**3e**–**3f**, **3h**, **3k**; 83–90% yield). Furthermore, cyclic olefins were well tolerated and afforded the desired fused dihydrobenzofurans in good yields (**3i**–**3j**, 91–94% yield). The presence of electron-donating groups on the substrates proved to be necessary for this reaction to occur. Electron-donating groups^23d^ on phenol stabilize the electron-deficient phenoxonium intermediate generated in situ, which is then trapped by electron-rich olefins^23a^ to promote oxidative cyclizations. Furthermore, the optimized conditions were applied to the gram-scale synthesis of dihydrobenzofuran **3b**, which was then successfully transformed to natural product (±)-conocarpan in four steps in good overall yield (see SI, Scheme S4). Furthermore, another natural product, (±)-pterocarpin, was synthesized in one step using our established COF-based photocatalytic protocol. In particular, the oxidative [3+2] cycloaddition between sesamol and 7-methoxy-2*H*-chromene photocatalyzed by **Hex-Aza-COF-3** afforded (±)-pterocarpin in 85% yield (see SI, Scheme S5).

**Table 2:**
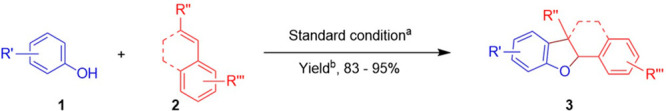

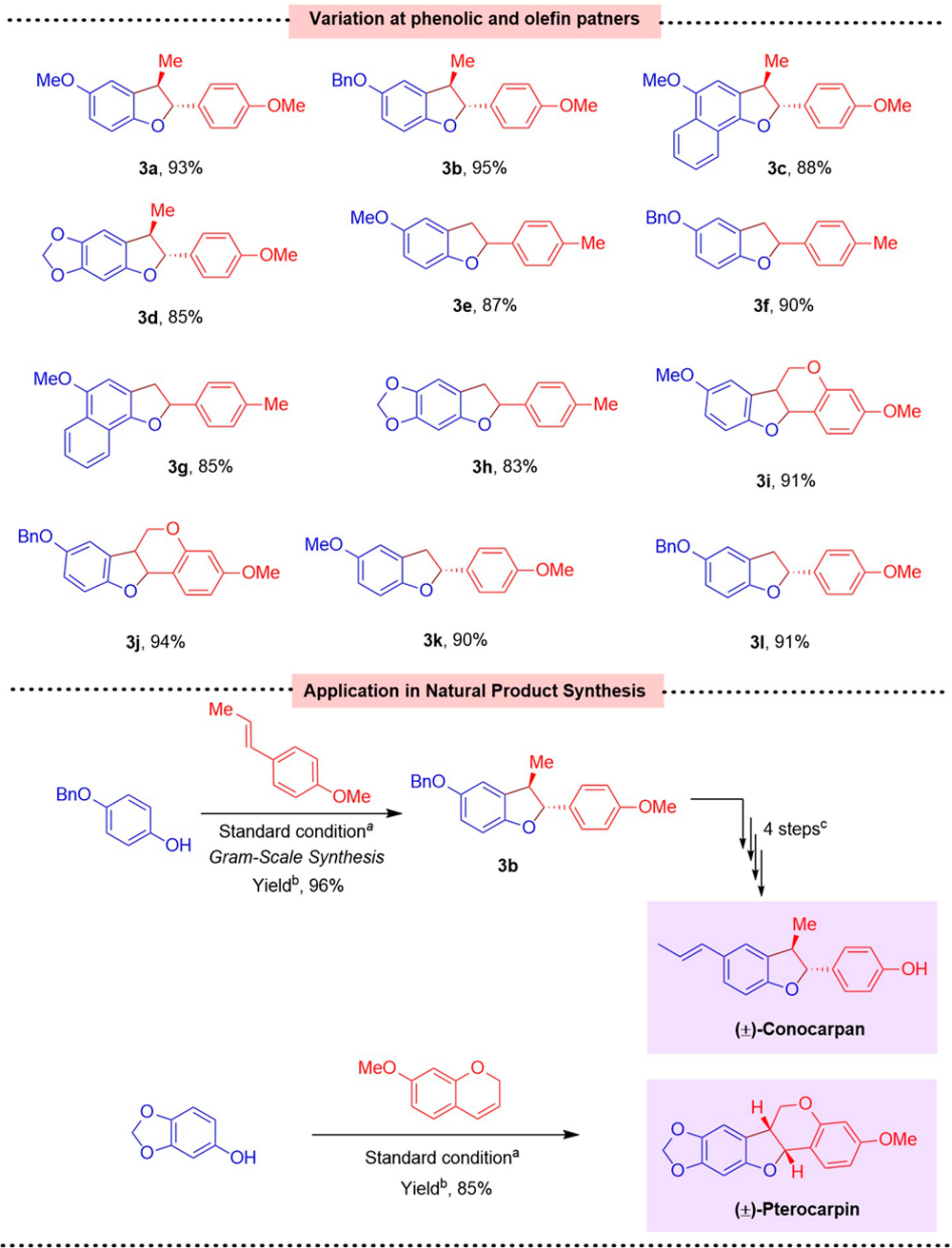
Substrate Scope and Application of **Hex-Aza-COF-3** in Natural Product Synthesis

a Standard conditions: **1** (0.2 mmol), **2** (0.22 mmol), 2.5 mol % **Hex-Aza-COF-3**, 0.24 mmol of (NH_4_)_2_S_2_O_8_, 5 mL of CH_3_CN, white LEDs, room temperature, 9 h.

b Isolated yield.

c See SI for details (Scheme S4).

**Figure 2:**
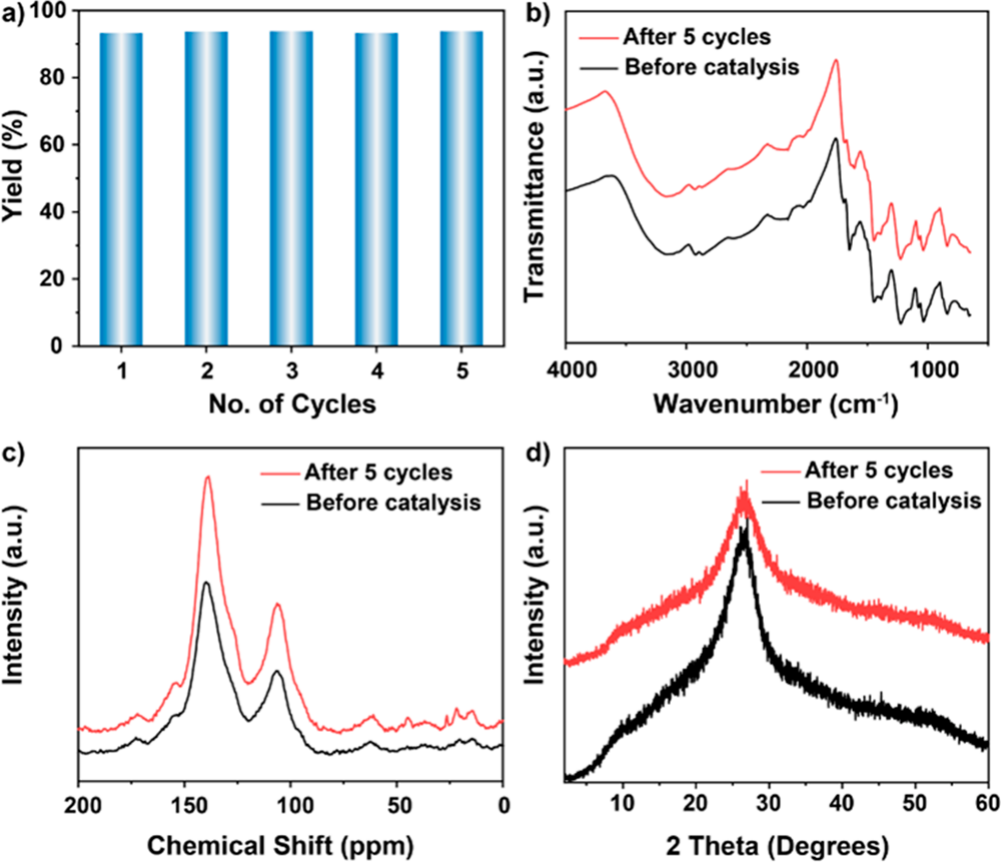
Assessment of the reusability of **Hex-Aza-COF-3**. The reusability tests were conducted under standard conditions as shown in Table 1, entry 1. (a) Plot of number of cycles vs yield; (b) FTIR analysis of **Hex-Aza-COF-3** before and after five catalytic cycles; (c) ^13^C CP-MAS NMR analysis of **Hex-Aza-COF-3** before and after five catalytic cycles; (d) PXRD analysis of **Hex-Aza-COF-3** before and after five catalytic cycles.

In addition to the activity and substrate scope of a catalyst, its recyclability is an important parameter in heterogeneous catalysis for practical application. Therefore, the reusability and stability of **Hex-Aza-COF-3** in the oxidative [3+2] cycloaddition under white light irradiation was explored. **Hex-Aza-COF-3** could be easily separated from the reaction mixture by centrifugation and used in the next run without any special treatment or reactivation procedure (see SI, Section S5). The COF retained its photocatalytic activity for up to five consecutive cycles (Figure 2a), demonstrating its reusability.

The recycled COF maintained the initial framework connectivity, morphology, crystallinity, and porosity, as evidenced by unchanged FTIR (Figure 2b), ^13^C CP-MAS NMR (Figure 2c), PXRD (Figure 2d), and N_2_ sorption isotherms (see SI, Figure S6b), suggesting the excellent stability of **Hex-Aza-COF-3**. Furthermore, the COF-catalyzed oxidative [3+2] cycloaddition reaction was explored using sunlight as the light source rather than white LEDs. To our delight, the product was obtained in high yield (91%) (see SI, Scheme S6). These results demonstrate the potential of the **Hex-Aza-COF-3** photocatalyst for practical application.

**Figure 3:**
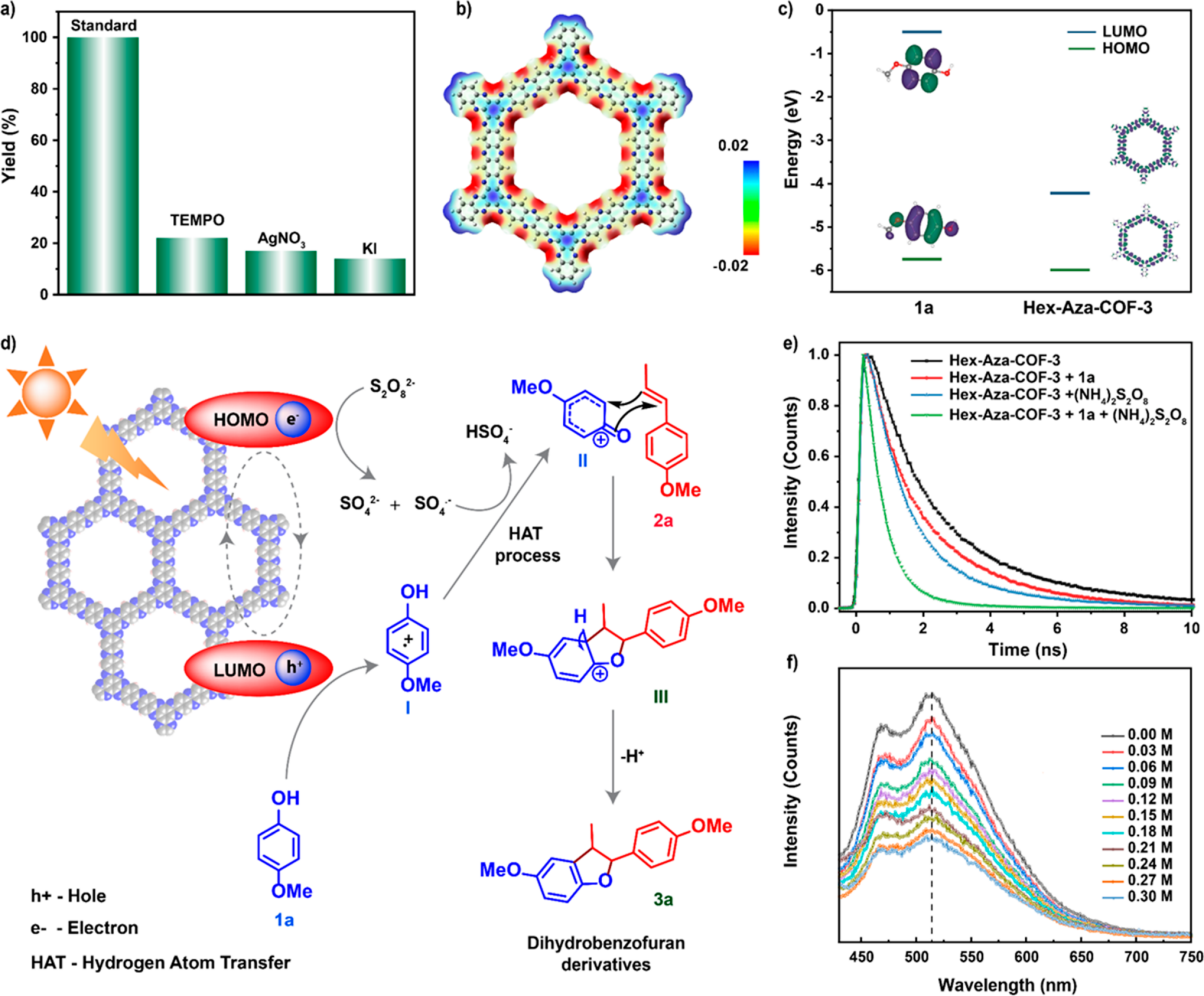
(a) Controlled scavenger experiments; (b) electrostatic potential surface of **Hex-Aza-COF-3**; (c) calculated energies and electronic charge densities of the HOMO and LUMO of substrate **1a** and **Hex-Aza-COF-3**; (d) proposed reaction mechanism; (e) photoluminescence lifetime quenching experiments measured using time-correlated single photon counting experiments; (f) steady-state emission quenching of **Hex-Aza-COF-3** with substrate **1a**.

**Figure 4:**
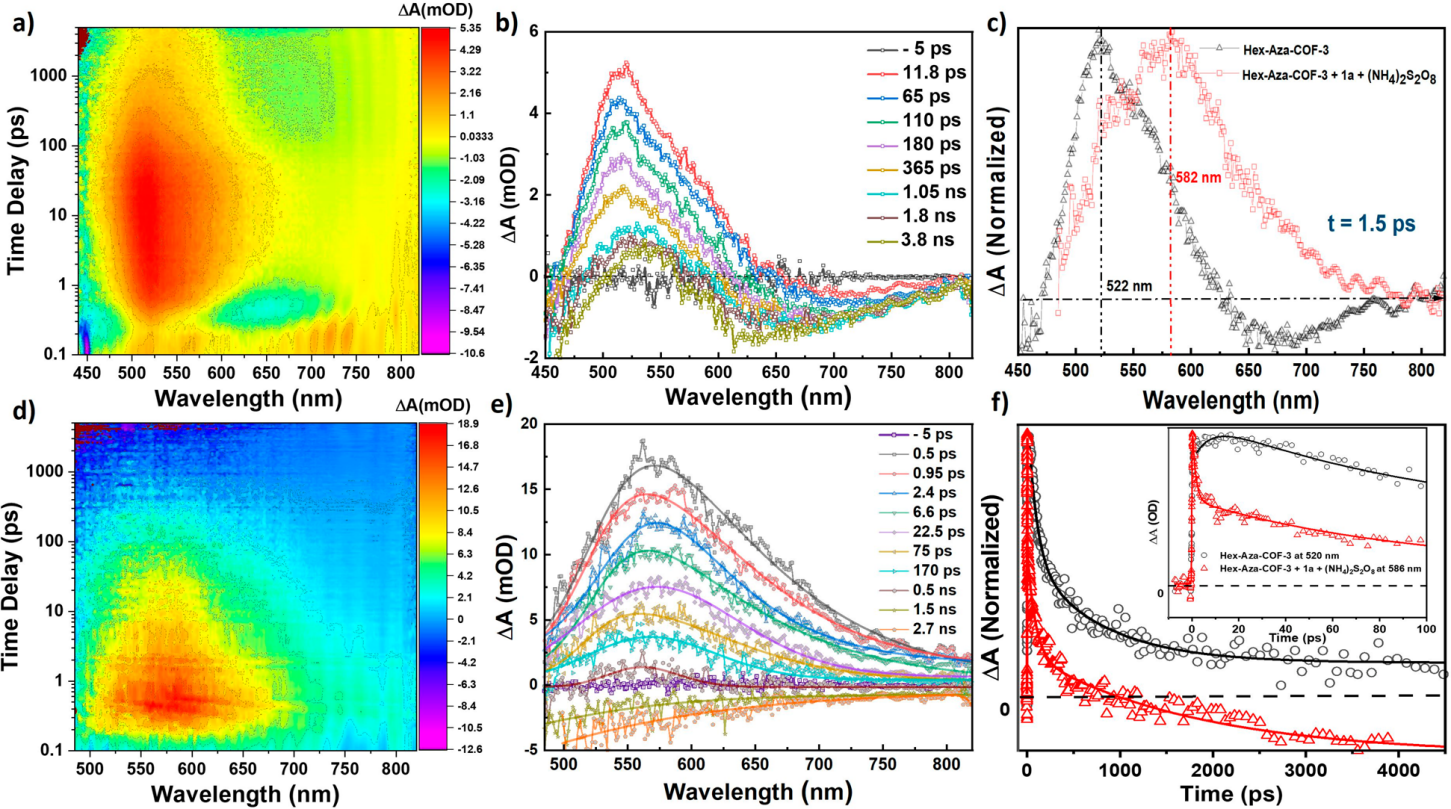
Transient absorption (TA) spectroscopic studies. (a, b) TA contour plot and spectrum of **Hex-Aza-COF-3**; (c) comparison of the TA spectra at 1.5 ps for **Hex-Aza-COF-3** with and without **1a** and (NH_4_)_2_S_2_O_8_; (d, e) TA contour plot and spectrum of **Hex-Aza-COF-3** with **1a** and (NH_4_)_2_S_2_O_8_; (f) kinetic traces of **Hex-Aza-COF-3** at 520 nm and **Hex-Aza-COF-3** with **1a** and (NH_4_)_2_S_2_O_8_ at 586 nm at late and early (inset) times. λ_exc_ = 400 nm.

To systematically understand the reaction mechanism of this photocatalysis process, a number of controlled experiments were performed (Table 1 and Figure 3a) and monitored by ^1^H NMR analysis. The reactions were conducted under the optimized conditions in the presence of TEMPO as a radical scavenger, AgNO_3_ as an e^–^ scavenger, and KI as an h^+^ scavenger (Figure 3a). In the presence of TEMPO, the yield of the desired product considerably decreased (∼22%), which confirms that the reaction proceeds via a radical pathway. Similarly, the presence of either AgNO_3_ or KI inhibited the product formation (17% and 14%, respectively), suggesting that both reductive e^–^ and oxidative h^+^ participate in the photocatalytic process. Furthermore, DFT calculations (Figure 3b,c) were performed on **Hex-Aza-COF-3** and substrate **1a**. Note that **Hex-Aza-COF-3** was treated as a macromolecule with H terminations to represent the periodical COF structure. The calculated bandgap of molecular **Hex-Aza-COF-3** was 1.77 eV (Figure 3c), which in agreement with the value obtained in the optical experiments (Figure 1b, inset). As shown in Figure 3b, the mapping of the electrostatic potential surface of **Hex-Aza-COF-3** indicates that the segments of −C–N=C– tend to withdraw electrons because of the electronegativity in the vicinity (shown in red). Figure 3c shows the calculated energies for the LUMO and HOMO of these molecules. Once **Hex-Aza-COF-3** is photoexcited, **1a** can capture an h^+^ from **Hex-Aza-COF-3** to become a radical cation because the HOMO level of **1a** is higher than that of **Hex-Aza-COF-3**. Moreover, the e^–^ transfer from **Hex-Aza-COF-3** to **1a** is prohibited because the LUMO level of **1a** is much higher. According to our controlled experiments, DFT calculation studies, and previous literature,^23d^ a plausible reaction mechanism was proposed, as shown in Figure 3d. Upon light irradiation, **Hex-Aza-COF-3** afforded effective e^–^–h^+^ charge separation. Then, h^+^ oxidizes phenolic substrate **1a** to radical cation intermediate **I** and e^–^ reduces persulfate to form SO_4_^2–^ and SO_4_^•–^ species. The in situ generated SO_4_^•–^ is known to be a good hydrogen atom transfer agent;^25^ therefore, it could extract the H^•^ from phenolic radical cation **I** to produce resonance-stabilized phenoxonium cation intermediate **II**, which undergoes [3+2] cycloaddition with electron-rich olefin **2a** to form cyclized intermediate **III**. Intermediate **III** rapidly undergoes aromatization to afford the desired product **3a**. Furthermore, PL lifetime, Stern–Volmer analysis, and TA spectroscopic studies were performed to support the proposed reaction mechanism. First, the excited-state lifetimes of pure **Hex-Aza-COF-3** and those of **Hex-Aza-COF-3** in the presence of either **1a** or (NH_4_)_2_S_2_O_8_ or both were investigated by time-correlated single photon counting measurements (Figure 3e). A decrease in the excited-state lifetime of **Hex-Aza-COF-3** (τ_avg_ = 4.6 ns) was observed with either **1a** (τ_avg_ = 2.2 ns) or (NH_4_)_2_S_2_O_8_ (τ_avg_ = 2.4 ns), and the presence of both **1a** and (NH_4_)_2_S_2_O_8_ decreased the excited-state lifetime further (τ_avg_ = 1.2 ns), suggesting the involvement of both h^+^ and e^–^ in the reaction mechanism. Subsequently, a steady-state emission quenching experiment was performed (Figure 3f) for **Hex-Aza-COF-3** with substrate **1a**. Noticeably, a decrease in the intensity of the emission spectra with increasing the concentration of **1a** was observed. Finally, TA spectroscopic experiments were conducted for **Hex-Aza-COF-3** dispersed in dimethylformamide (Figure 4), which revealed an excited-state absorption (ESA) band for **Hex-Aza-COF-3** centered at 522 nm, reaching its peak intensity after 11.8 ps (Figure 4a,b). This time scale is associated with the reorganization of solvent molecules around **Hex-Aza-COF-3** in the charge transfer state. The evolving negative signal in the region of 570–800 nm is the ground-state bleach from the tail end of the broad ground-state absorption band (Figure 4b). The TA spectrum of **Hex-Aza-COF-3** with (NH_4_)_2_S_2_O_8_ and **1a** (Figure 4d,e) demonstrated a broader and red-shifted (Δλ = 60 nm) ESA signal centered at 582 nm. A comparison of spectra **4b** and **4e** at early times (1.5 ps) suggests two different excited-state conditions for **Hex-Aza-COF-3** while in the presence of (NH_4_)_2_S_2_O_8_ and **1a** (Figure 4c). The shoulder at 522 nm (dashed black line) corresponds to the exact position of the lone **Hex-Aza-COF-3** ESA shown in Figure 4b and is indicative of an intramolecular charge transfer state that is present with and without (NH_4_)_2_S_2_O_8_ and **1a**. This suggests that the charge transfer between the **Hex-Aza-COF-3** subunits (phenazine and HAT) occurs at early times after excitation and precedes the intermolecular e^–^ and h^+^ transfer with S_2_O_8_^2–^ and **1a**, respectively. The new band at 582 nm (red dashed line) suggests that the photocatalyst possesses a new ionic excited state caused by the exchange of e^–^ and h^+^, which is stabilized by the polar solvent. A comparison of the dynamics between the two conditions (Figure 4f) shows a faster decay of the excited state **Hex-Aza-COF-3** with (NH_4_)_2_S_2_O_8_ and **1a** when compared to the lone **Hex-Aza-COF-3**. This feature is congruent with the quenching of the excited state of **Hex-Aza-COF-3** that occurs during the rapid transfer of the e^–^ and h^+^ between the photocatalyst and (NH_4_)_2_S_2_O_8_ and **1a**. The negative signal at around 1 ns is associated with the **Hex-Aza-COF-3** (with (NH_4_)_2_S_2_O_8_ and **1a**) and is assigned to separated ionic species within the polar solvent. μs-TA measurements (see SI, Figure S8) reveal that the separated ionic species take 48.1 μs to return to their neutral state. The kinetics of the lone **Hex-Aza-COF-3** also reveal a negative signal, but it recovers faster at 12.2 μs. This is associated with the solvent stabilization of the e^–^ and h^+^ separated charges (free ions) on the **Hex-Aza-COF-3**.

## Conclusion

In summary, we demonstrated the photophysical and electrochemical properties of **Hex-Aza-COF-3** and its successful deployment as a visible-light-activated heterogeneous photocatalyst for the oxidative [3+2] cycloaddition of phenols with olefins to form 2,3-dihydrobenzofurans, which are medicinally important skeletons and critical building units for various pharmaceutical agents and bioactive natural products. This photocatalytic process was applied to the synthesis of natural products (±)-conocarpan and (±)-pterocarpin via oxidative [3+2] cycloaddition as the key step. Markedly, reusability experiments confirmed the high stability of **Hex-Aza-COF-3**, which maintained its activity, porosity, crystallinity, covalent bonding, and morphology. Moreover, DFT calculations, PL quenching experiments, Stern–Volmer analysis, TA spectroscopic measurements, and controlled scavenger experiments permitted the proposal of a plausible reaction mechanism of charge transfer and charge separation for this photocatalytic cycle. We believe that this COF-based photocatalyst not only provides a green and facile approach to access 2,3-dihydrobenzofuran scaffolds but also widens the application scope of metal-free COF-based photocatalysts for the sustainable development of additional complex organic transformations.

## Supporting Information Available

Detailed experimental procedures, characterization data, DFT calculations, and copies of ^1^H and ^13^C NMR of all synthesized compounds (PDF)
