# Supplementary material for: A Tailored COF for Visible-Light Photosynthesis of 2,3-Dihydrobenzofurans
Source: J Am Chem Soc. 2023 Feb 24;145(9):5074–82. doi: 10.1021/jacs.2c10471 (PMC9999419; doi:10.1021/jacs.2c10471)
Supplement: Supplementary file 1 — ja2c10471_si_001.pdf [file ja2c10471_si_001.pdf]

Supporting Information

A Tailored COF for Visible-light Photosynthesis of 2,3-Dihydrobenzofurans

Prakash T. Parvatkar,[1] Sharath Kandambeth,[1] Aslam C. Shaikh,[1] Issatay Nadinov,[2] Jun Yin,[2,3] Vinayak S. Kale,[1] George Healing,[2] Abdul-Hamid Emwas,[4] Osama Shekhah,[1] Husam N. Alshareef,[5] Omar F Mohammed[2] and Mohamed Eddaoudi[1]*

[1]Functional Materials Design, Discovery and Development Research Group (FMD3), Advanced Membranes and Porous Materials Center (AMPM), Division of Physical Science and Engineering (PSE), King Abdullah University of Science and Technology (KAUST), Thuwal 23955-6900, Kingdom of Saudi Arabia.

[2]Advanced Membranes and Porous Materials Center (AMPM), Division of Physical Science and Engineering (PSE), King Abdullah University of Science and Technology (KAUST), Thuwal 23955-6900, Kingdom of Saudi Arabia.

[3]Department of Applied Physics, The Hong Kong Polytechnic University, Hung Hom, Kowloon, 999077 Hong Kong, P. R. China.

[4]Core Laboratories, King Abdullah University of Science and Technology (KAUST), Thuwal 23955-6900, Kingdom of Saudi Arabia.

[5]Division of Physical Science and Engineering (PSE), King Abdullah University of Science and Technology (KAUST), Thuwal 23955-6900, Kingdom of Saudi Arabia

E-mail: mohamed.eddaoudi@kaust.edu.sa

Table of Contents

Section S1: General Remarks S3

Section S2: Synthesis and Characterization of Hex-Aza COF-3 .... S4

1. Synthesis of 2,3,6,7-Tetraamino-phenazine Hydrochloride (4) S4
2. Synthesis of Hexaazatrinaphthylene (5) S5
3. Synthesis of Hex-Aza-COF-3 .... S5
4. Characterization Data for Hex-Aza-COF-3 .... S6

Section S3: Hex-Aza-COF-3 Photocatalysis S7

1. Photochemical Reaction Setup S7
2. General Procedure for the Photocatalytic Reaction S7
3. Time-Dependent Studies S8
4. Light-on/Light-off Experiment S8
5. Comparison of the Catalytic Activities of Present Work with the Reported Work S9
6. Computational Methods and UV-Vis Spectroscopy Studies S10
7. Scope of the Photocatalytic Reaction S11
8. Characterization Data for 2,3-Dihydrobenzofuran Derivatives 3a–l S12
9. Gram-Scale Synthesis of 2,3-Dihydrobenzofuran Derivative 3b S18

Section S4: Application of Hex-Aza-COF-3 Photocatalysis to Natural Product Synthesis S18

1. Synthesis of (±)-Conocarpan S18
2. Synthesis of (±)-Pterocarpin S20

Section S5: Photocatalyst Recyclability Studies S22

Section S6: Stern–Volmer Analysis S23

Section S7: µs-Transient Absorption measurement S23

Section S8: Photocatalysis using Sunlight as the Light Source S24

Section S9: References S25

Section S10: ^{1}H and ^{13}C NMR Spectra S26

Section S1: General Remarks

Hex-Aza COF-3 and the organic ligands **4** and **5** used in this study were prepared according to procedures reported in the literature. Other starting materials, reagents, and solvents were purchased from commercial sources and used without further purification unless otherwise stated. ^{1}H and ^{13}C NMR spectra were recorded at ambient temperature on a Bruker Advance III instrument operating at 400 and 500 MHz (^{1}H NMR) and 100 and 125 MHz (^{13}C NMR) in the solvent indicated using the signal of the residual solvent as an internal standard ($CDCl_3$ δ 7.26 ppm or DMSO-d_6 δ 2.50 ppm for ^{1}H NMR and $CDCl_3$ δ 77.16 ppm or DMSO-d_6 δ 39.52 ppm for ^{13}C NMR). ^{1}H NMR data are reported as follows: chemical shift, multiplicity (s = singlet, d = doublet, dd = doublet of doublets, t = triplet, q = quartet, m = multiplet), coupling constant (Hz), and integration. Solid-state ^{13}C NMR spectra were recorded using a Bruker 400 AVANAC III spectrometer equipped with a 4 mm double-resonance CP MAS Bruker probe. All ^{13}C cross-polarization magic-angle-spinning (CP-MAS) NMR spectra were recorded at a resonance frequency of 100.62 MHz under a spinning rate of 12 kHz using a 4 mm triple-resonance Bruker MAS probe. To achieve a sufficient signal-to-noise ratio in a reasonable time, the ^{13}C CP-MAS NMR spectra were recorded by collecting 12 k scans with a recycle delay time of 7 s. The temperature was maintained at 298 K for all the experiments and the CP contact time was set to 2 ms using ramp 100 for variable amplitude CP. Bruker Topspin 3.5pl7 software (Bruker BioSpin, Rheinstetten, Germany) was used for both data collection and spectral processing. Thin-layer chromatography (TLC) was performed using silica gel 60 F_{254} precoated plates and visualized with exposure to UV light (254 nm) or by iodine staining. Flash chromatography was performed on Biotage (Model: Isolera). Powder X-ray diffraction (PXRD) patterns were obtained on a D8 Advance X-ray diffractometer (Bruker, Germany) with Cu Kα radiation (λ = 1.5406 Å). Fourier transform infrared (FTIR) spectra (4000–600 cm^{-1}) were recorded on a Thermo Scientific Nicolet 6700 apparatus. Low-pressure N_2 adsorption studies of the **Hex-Aza COF-3** were conducted on a fully automated Autosorb-IC micropore gas analyzer (Quantachrome Instruments) at relative pressures up to 1 atm. UV–vis absorption spectra of **Hex-Aza-COF-3** suspensions were collected using a Shimadzu UV-1900 instrument. Fluorescence spectra were measured using a microplate reader (SpectraMax M5, Molecular Devices). Photoluminescence lifetime was measured using the time-correlated single photon counting technique (FluoTime 250, fluorescence lifetime spectrometer). Femtosecond transient absorption spectroscopic measurements were performed using a Helios spectrometer (Ultrafast Systems). The beam from an Astrella Ti:Saph (Coherent) pulsed laser (100 fs, 1 kHz, 800 nm) was split and directed to an optical parametric amplifier (Newport Spectra-Physics) to tune the excitation wavelength to 400 nm. This beam was focused on the sample after passing through a mechanical chopper (500 Hz). The other 800 nm branch of the beam went to a delay stage to obtain the time resolution and was then focused on a CaF_2 crystal to generate a white-light probe. The white light was split to a reference channel

and overlapped in the sample with the pump beam. A cyclic voltammetry (CV) analysis was conducted using CH Instruments Electrochemical Analyzer/Workstation (Model 700E Series). The electrode ink was prepared using Hex-Aza-COF-3 (9 mg) and Nafion binder (1 mg) in water:ethanol (1:1), ultrasonicated for a few hours, coated onto a 5 mm glassy carbon electrode, and perfectly dried. Glassy carbon in anhydrous acetonitrile (CH_3CN) containing 0.1 M tetrabutylammonium hexafluorophosphate was used as the working electrode, Ag/AgCl in CH_3CN as the reference electrode, and Pt as the counter electrode with a scan rate of 100 mV s^{-1}. Light irradiation experiments were performed with a ZLED CLS 6000 lamp (ZETT OPTICS) comprising white LEDs as a cold light source. The light flux was ~650 lumen with an approximate wavelength range from 400 to 700 nm. For all experiments, the light intensity was adjusted to 100%.

Section S2: Synthesis and Characterization of Hex-Aza COF-3

1. Synthesis of 2,3,6,7-Tetraamino-phenazine Hydrochloride (4)

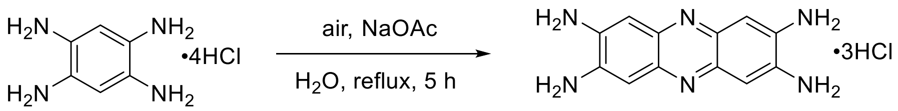

4

Scheme S1: Synthesis of monomer **4**

Compound **4** was prepared following a previously reported procedure (**Scheme S1**).[1] To a solution of 1,2,4,5-tetraamino-benzene-tetrahydrochloride (3.25 g, 11.44 mmol) in distilled water (30 mL), sodium acetate (7.51 g, 37.69 mmol) was added, and the reaction mixture was refluxed while passing compressed air through the solution for 5 h. The reaction mixture was allowed to cool to room temperature, causing the precipitation of a solid. The solid was separated by filtration, washed with water, and dried under vacuum to afford 2,3,6,7-tetraamino-phenazine hydrochloride (**4**; 2.19 g, 69% yield) as a deep-purple solid. The spectroscopic data are consistent with those previously reported in the literature.[1]

^{1}H NMR (400 MHz, DMSO-d_6) δ = 6.78 (s, 4H), 5.44 (s, 8H).

^{13}C NMR (100 MHz, DMSO-d_6) δ = 140.68, 138.74, 104.69.

2. Synthesis of Hexaazatrinaphthylene (5)

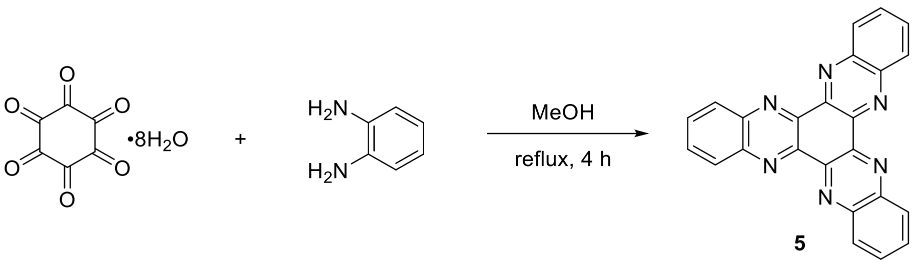

Scheme S2: Synthesis of monomer **5**

Compound **5** was prepared using a reported procedure (**Scheme S2**).[2] To a mixture of hexaketocyclohexane octahydrate (1.52 g, 4.87 mmol) and 1,2-phenylenediamine (1.74 g, 16.09 mmol) anhydrous methanol (50 mL) was added and the mixture was refluxed for 4 h. The reaction mixture was cooled to room temperature, and a solid precipitated out. The solid was separated by filtration, washed with methanol, and dried under vacuum to give **6** (2.19 g, 89% yield, based on hexaketocyclohexane octahydrate) as yellow-green flake solids. The spectroscopic data are consistent with those previously reported in the literature.[2]

^{1}H NMR (400 MHz, $CDCl_3$) δ = 8.72 (dd, *J* = 6.6, 3.4 Hz, 6H), 8.06 (dd, *J* = 6.6, 3.4 Hz, 6H).
^{13}C NMR (125 MHz, $CDCl_3$) δ = 143.58, 143.55, 132.28, 130.66.

3. Synthesis of Hex-Aza-COF-3

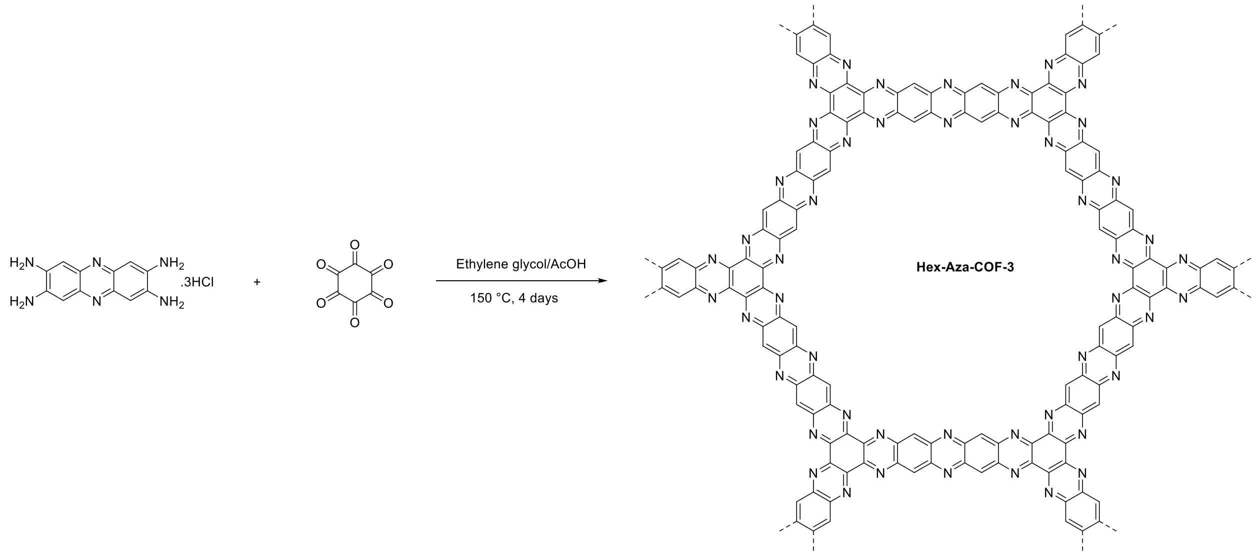

Scheme S3: Synthesis of **Hex-Aza-COF-3**

Hex-Aza-COF-3 was prepared as per a reported procedure (**Scheme S3**)[3] via solvothermal condensation reaction of hexaketocyclohexane octahydrate (93.5 mg, 0.30 mmol) and 2,3,6,7-tetraaminophenazine hydrochloride (157.1 mg, 0.45 mmol) in a 1:1 mixture of ethylene glycol (5 mL) and 6.0 M acetic acid (5 mL). Initially, reactants and solvents were transferred to 70 mL screw-capped Pyrex tubes under nitrogen atmosphere and sonicated for 15 min. After sonication, the Pyrex tubes were transferred to a preheated oven at 65 °C. The Pyrex tubes were then incubated at 65 °C for 4 h, and the temperature of the oven was then slowly raised to 120–150 °C. After four days of reaction, the Pyrex tubes were removed from the oven. **Hex-Aza-COF-3** was isolated as a black powder by filtration, washed with dimethylformamide (DMF), acetone, and water and activated at room temperature under vacuum for 12 h. This activated COF catalyst was directly employed for the photocatalytic reaction.

4. Characterization Data for Hex-Aza-COF-3

The data obtained for **Hex-Aza-COF-3** are in full agreement with those previously published in the literature.[3]

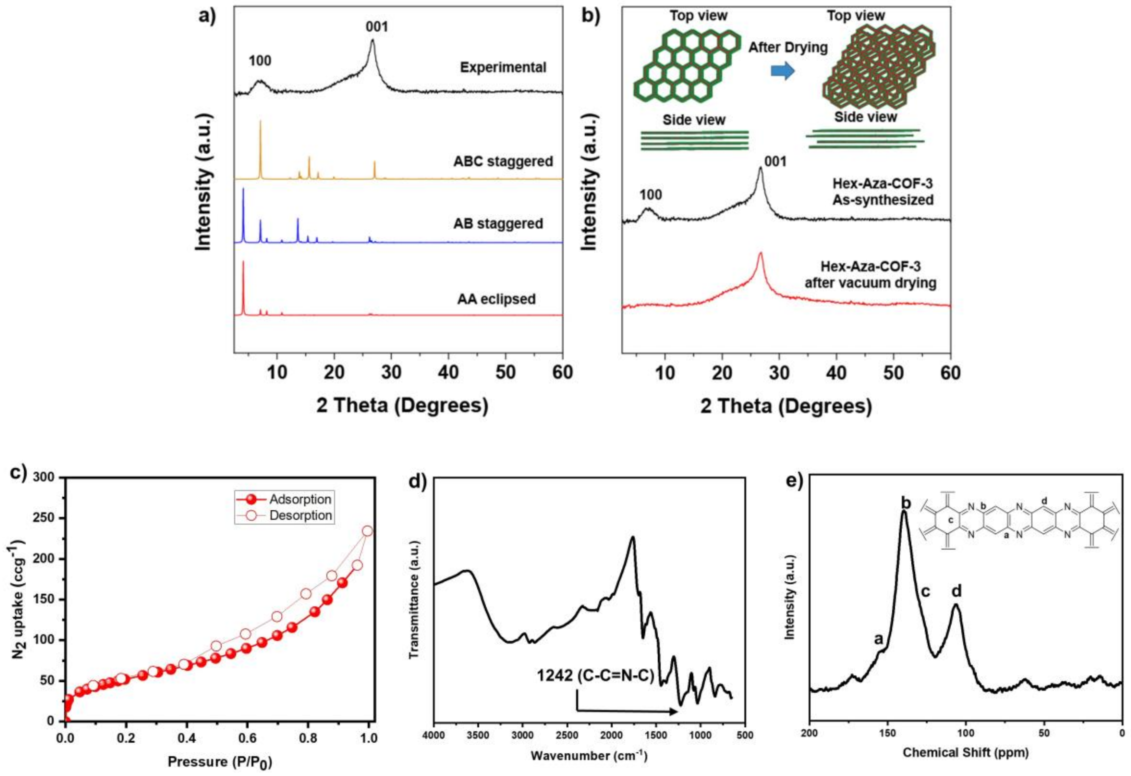

Figure S1: Characterization of **Hex-Aza-COF-3:** a) Comparison of experimental PXRD pattern of as-synthesized **Hex-Aza-COF-3** with different simulated stacking models; b) Change in PXRD pattern of **Hex-Axza-COF-3** before and after vacuum drying; c) N_2 adsorption isotherm at 77 K; d) FTIR; e) ^{13}C CP-MAS NMR.

Section S3: Hex-Aza-COF-3 Photocatalysis

1. Photochemical Reaction Setup

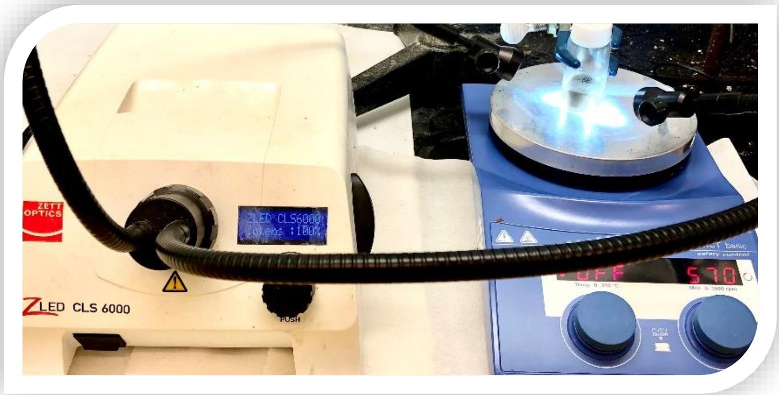

Figure S2: Photochemical setup for the photocatalysis

2. General Procedure for the Photocatalytic Reaction

A 20 mL scintillation vial equipped with a stir bar was charged with **Hex-Aza-COF-3** (0.05 mmol) and CH_3CN (5 mL). Subsequently, phenol **1** (0.2 mmol), olefin **2** (0.22 mmol), and ammonium persulfate (0.24 mmol) were added. The reaction mixture was sonicated for 15 min, followed by stirring at room temperature for 9 h under white LED irradiation using ZLED CLS 600 (ZETT OPTICS) as the light source at full power. The reaction mixture was transferred to a centrifuge tube and centrifuged at 5000 rpm for 5 min, and the liquid phase was separated. To the residue was added CH_3CN (5 mL), mixed thoroughly, centrifuged at 5000 rpm for 5 min, and the liquid phase was separated. The combined liquid phase was concentrated under reduced pressure, and the crude product was purified by Flash chromatography on a Biotage Snap Cartridge (KP-Sil 10 g) using a gradient solvent system (5% to 20% ethyl acetate in hexanes) to afford product **3**.

3. Time-Dependent Studies

These experiments were performed as per the general procedure using 4-methoxyphenol (75 mg, 0.6 mmol), *trans*-anethole (99 µL, 0.66 mmol), ammonium persulfate (165 mg, 0.72 mmol), and **Hex-Aza-COF-3** (12.6 mg, 0.015 mmol). The progress of the reaction was monitored by ^{1}H NMR analysis (**Figure S3**).

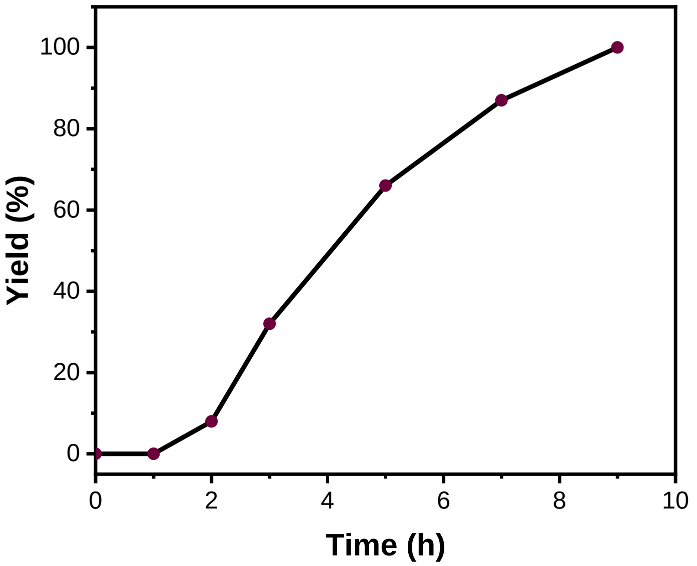

Figure S3: Time-dependent curve

4. Light-on/Light-off Experiment

This experiment was performed as per the general procedure using 4-methoxyphenol (150 mg, 1.2 mmol), *trans*-anethole (198 µL, 1.32 mmol), ammonium persulfate (330 mg, 1.44 mmol), and **Hex-Aza-COF-3** (25.2 mg, 0.015 mmol) in CH_3CN (15 mL). The light was switched on and off every 3 h, and the samples taken after each time interval with light on and light off were analyzed by ^{1}H NMR analysis (**Figure S4**).

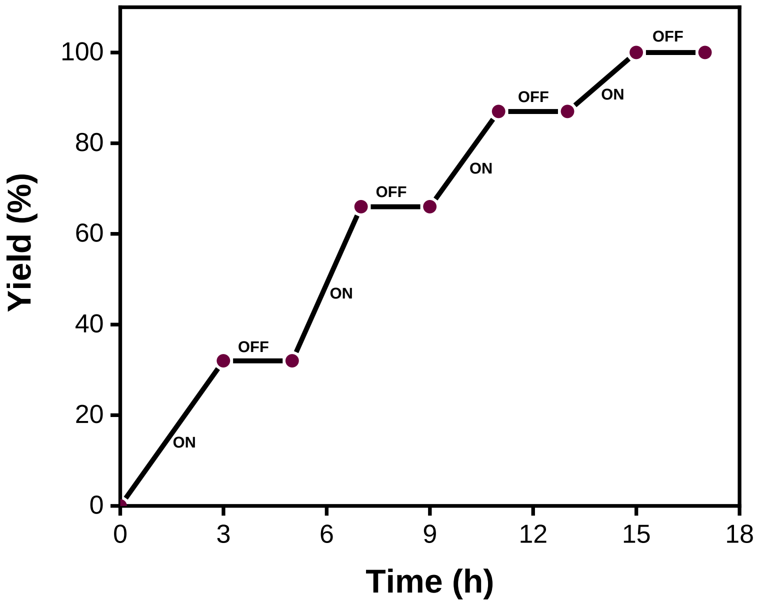

Figure S4: Light-on/light-off experiment over time

5. Comparison of the Catalytic Activities of Present Work with the Reported Work

Table S1. Comparison of the **Hex-Aza-COF-3** with other reported Photocatalyst for the Oxidative [3+2] Cycloaddition of Phenol with Olefin

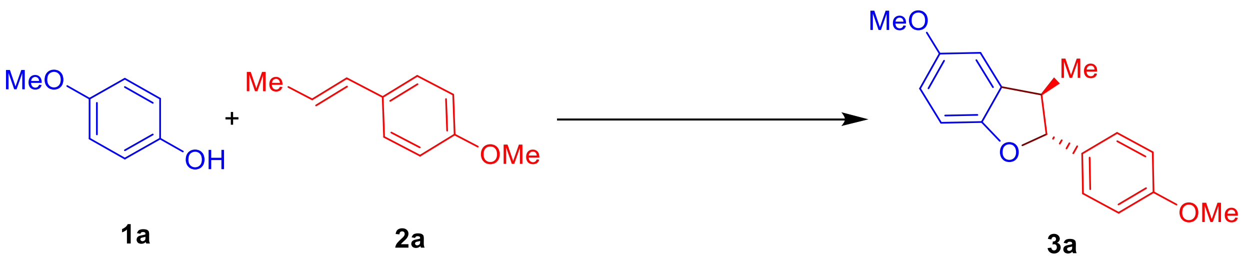

1a 2a 3a

Entry	Photocatalyst	Light Source	Catalyst Loading (mol%)	Time (h)	Yield (%)	Ref.
1	$Ru(bpz)_3(PF_6)_2$	Fluorescent light bulb	5	27	84	4
2	Aminoacridinium	Blue LED	2.5	40	77	5
3	Hex-Aza-COF-3	White LED	2.5	9	93	Present work

6. Computational Methods and UV-Vis Spectroscopy Studies

Density functional theory (DFT) calculations were performed to optimize the ground-state geometries of substrate **1a**, **4**, **5** and **Hex-Aza-COF-3** using the B3LYP functional and the 6-311G(d,p) basis set as implemented in Gaussian09 program (Revision D. 01). The solvent effects of CH_3CN (ε = 35.688) were considered in the geometry optimizations and electronic calculations using the polarizable continuum model.

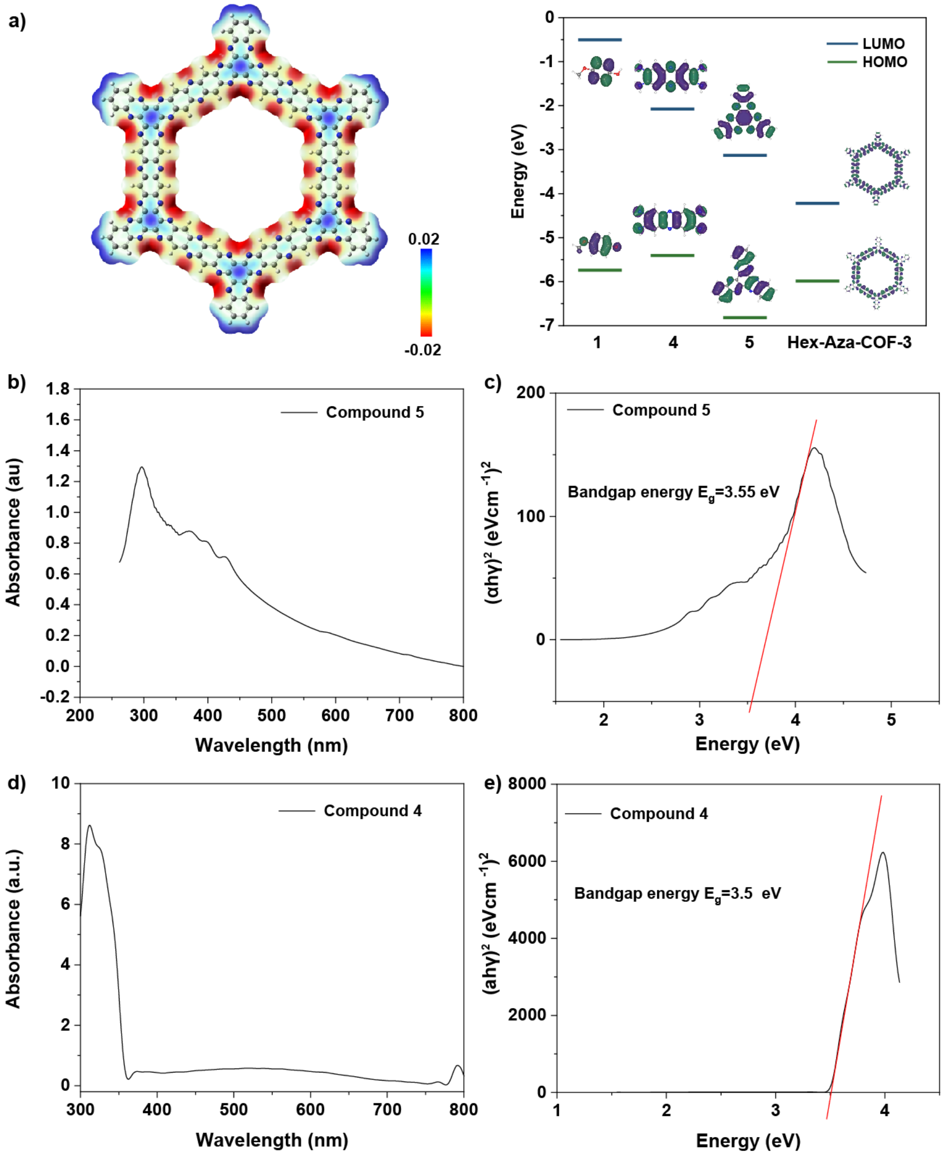

Figure S5: a) Theoretically calculated HOMO and LUMO levels of compound **4** and **5** in comparison to **Hex-Aza-COF-3.** b) UV–Vis absorption spectrum of compound **5**. c) Band gap energy (Eg) determination of compound **5** from the Tauc plot. d) UV–Vis absorption spectrum of compound **4**. e) Band gap energy (Eg) determination of compound **4** from the Tauc plot.

7. Scope of the Photocatalytic Reaction

Table S2. Synthesis of 2,3-Dihydrobenzofuran Derivatives 3a–l

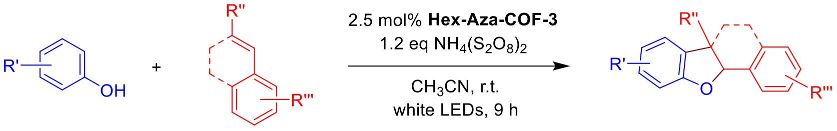

1 (1.0 equiv) **2** (1.1 equiv) **3**

Entry	Phenol 1	Olefin 2	2,3-DHB Product 3	Isolated Yield (%)
**a**	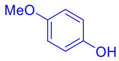	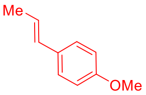	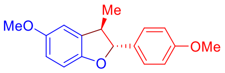	93
**b**	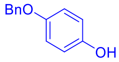	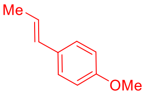	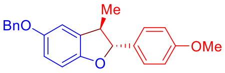	95
**c**	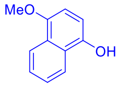	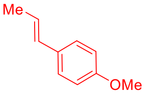	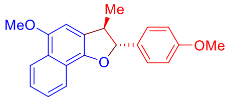	88
**d**	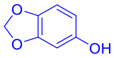	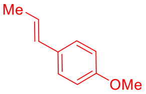	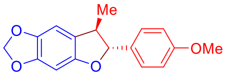	85
**e**	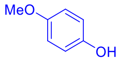	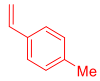	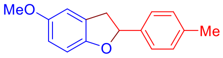	87
**f**	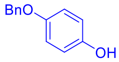	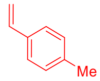	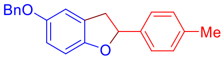	90
**g**	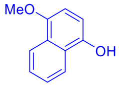	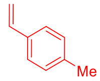	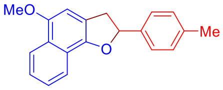	85
**h**	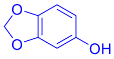	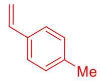	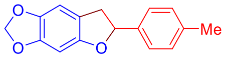	83
**i**	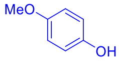	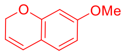	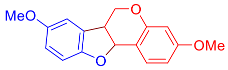	91
**j**	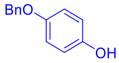	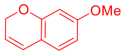	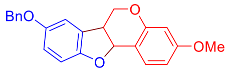	94
**k**	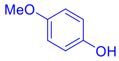	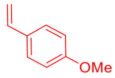	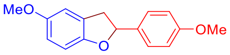	90
**l**	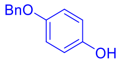	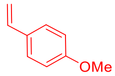	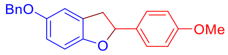	91

8. Characterization Data for 2,3-Dihydrobenzofuran Derivatives 3a–l

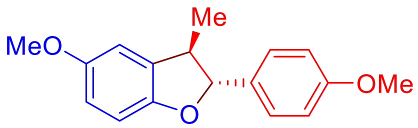

3a

5-Methoxy-2-(4-methoxyphenyl)-3-methyl-2,3-dihydrobenzofuran (3a) was prepared as per the general procedure using 4-methoxyphenol (25 mg, 0.2 mmol), *trans*-anethole (33 µL, 0.22 mmol), ammonium persulfate (55 mg, 0.24 mmol), and **Hex-Aza-COF-3** (4.2 mg, 0.005 mmol). Product **3a** was obtained as a colorless oil (50.6 mg, 93% yield) after purification by flash chromatography. The NMR data are in full agreement with those previously published in the literature.[4]

^{1}H NMR (400 MHz, $CDCl_3$) δ = 7.36 (dd, *J* = 8.5, 1.3 Hz, 2H), 6.91 (dd, *J* = 8.6, 1.4 Hz, 2H), 6.73 (q, *J* = 9.4, 8.8 Hz, 3H), 5.08 (d, *J* = 9.1 Hz, 1H), 3.82 (d, *J* = 1.4 Hz, 3H), 3.78 (s, 3H), 3.46–3.35 (m, 1H), 1.38 (d, *J* = 5.6 Hz, 3H).
^{13}C NMR (100 MHz, $CDCl_3$) δ = 159.76, 154.52, 153.38, 133.26, 132.80, 127.81, 114.13, 112.97, 110.21, 109.48, 92.74, 56.19, 55.46, 45.81, 17.70.
HRMS-ESI (m/z) calculated for $C_{17}H_{18}O_3Na$ $[M+Na]^+$ 293.1148, found 293.1191.

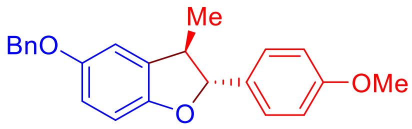

3b

5-(Benzyloxy)-2-(4-methoxyphenyl)-3-methyl-2,3-dihydrobenzofuran (3b) was prepared as per the general procedure using 4-benzyloxyphenol (40 mg, 0.2 mmol), *trans*-anethole (33 µL, 0.22 mmol), ammonium persulfate (55 mg, 0.24 mmol), and **Hex-Aza-COF-3** (4.2 mg, 0.005 mmol). Product **3b** was obtained as a white solid (65.8 mg, 93% yield) after purification by flash chromatography. The NMR data are in full agreement with those previously published in the literature.[4]
^{1}H NMR (400 MHz, $CDCl_3$) δ = 7.39 (dq, *J* = 25.8, 8.9, 8.5 Hz, 7H), 6.91 (d, *J* = 8.2 Hz, 2H), 6.82–6.72 (m, 3H), 5.08 (d, *J* = 9.1 Hz, 1H), 5.02 (s, 2H), 3.82 (s, 3H), 3.46–3.35 (m, 1H), 1.37 (d, *J* = 6.8 Hz, 3H).
^{13}C NMR (100 MHz, $CDCl_3$) δ = 159.77, 153.74, 153.62, 137.50, 133.29, 132.77, 128.69, 128.03, 127.81, 127.68, 114.13, 111.43, 109.48, 92.77, 71.21, 55.46, 45.79, 17.68.
HRMS-ESI (m/z) calculated for $C_{23}H_{22}O_3Na$ $[M+Na]^+$ 369.1461, found 369.1490.

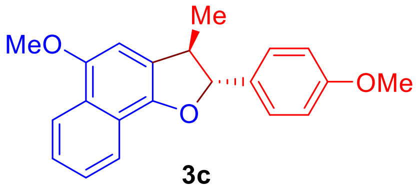

3c

5-Methoxy-2-(4-methoxyphenyl)-3-methyl-2,3-dihydronaphtho[1,2-b]furan (3c) was prepared as per the general procedure using 4-methoxynaphth-1-ol (35 mg, 0.2 mmol), *trans*-anethole (33 μL, 0.22 mmol), ammonium persulfate (55 mg, 0.24 mmol), and **Hex-Aza-COF-3** (4.2 mg, 0.005 mmol). Product **3c** was obtained as a white solid (56.6 mg, 93% yield) after purification by flash chromatography. The NMR data are in full agreement with those previously published in the literature.[4]

^{1}H NMR (400 MHz, $CDCl_3$) δ = 8.25–8.18 (m, 1H), 7.99–7.91 (m, 1H), 7.49–7.37 (m, 4H), 6.92 (d, *J* = 8.6 Hz, 2H), 6.68 (s, 1H), 5.30–5.26 (m, 1H), 3.99 (s, 3H), 3.82 (s, 3H), 3.65–3.56 (m, 1H), 1.48 (d, *J* = 6.8 Hz, 3H).

^{13}C NMR (100 MHz, $CDCl_3$) δ = 159.71, 150.50, 148.02, 133.62, 127.96, 127.73, 126.19, 125.62, 125.17, 123.63, 122.62, 121.51, 121.07, 114.16, 100.53, 92.65, 56.23, 55.48, 47.22, 18.94.

HRMS-ESI (m/z) calculated for $C_{21}H_{20}O_3Na$ $[M+Na]^+$ 343.1305, found 343.1349.

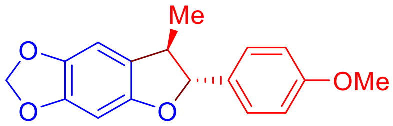

3d

6-(4-Methoxyphenyl)-7-methyl-6,7-dihydro-[1,3]dioxolo[4,5-f]benzofuran (3d) was prepared as per the general procedure using sesamol (27.6 mg, 0.2 mmol), *trans*-anethole (33 μL, 0.22 mmol), ammonium persulfate (55 mg, 0.24 mmol), and **Hex-Aza-COF-3** (4.2 mg, 0.005 mmol). Product **3d** was obtained as a colorless oil (48.3 mg, 85% yield) after purification by flash chromatography.

^{1}H NMR (400 MHz, $CDCl_3$) δ = 7.35 (d, *J* = 8.6 Hz, 2H), 6.91 (d, *J* = 8.7 Hz, 2H), 6.61 (s, 1H), 6.44 (s, 1H), 5.90 (d, *J* = 1.4 Hz, 2H), 5.08 (d, *J* = 8.9 Hz, 1H), 3.82 (s, 3H), 3.40–3.29 (m, 1H), 1.34 (d, *J* = 6.7 Hz, 3H).

^{13}C NMR (100 MHz, $CDCl_3$) δ = 159.89, 153.84, 147.54, 141.87, 132.87, 127.87, 123.35, 114.24, 93.37, 55.57, 45.58, 18.40.

HRMS-ESI (m/z) calculated for $C_{17}H_{16}O_4Na$ $[M+Na]^+$ 307.0941, found 307.0965.

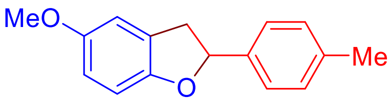

3e

5-Methoxy-2-(*p*-tolyl)-2,3-dihydrobenzofuran (3e) was prepared as per the general procedure using 4-methoxyphenol (25 mg, 0.2 mmol), 4-methylstyrene (29.2 µL, 0.22 mmol), ammonium persulfate (55 mg, 0.24 mmol), and **Hex-Aza-COF-3** (4.2 mg, 0.005 mmol). Product **3e** was obtained as a pale yellow solid (42.1 mg, 87% yield) after purification by flash chromatography. The spectroscopic data are in full agreement with those previously published in the literature.[6]

^{1}H NMR (400 MHz, $CDCl_3$) δ = 7.29 (d, *J* = 8.1 Hz, 2H), 7.17 (d, *J* = 7.9 Hz, 2H), 6.80–6.72 (m, 2H), 6.69 (dd, *J* = 8.7, 2.7 Hz, 1H), 5.70 (t, *J* = 8.8 Hz, 1H), 3.77 (s, 3H), 3.57 (dd, *J* = 15.7, 9.3 Hz, 1H), 3.19 (dd, *J* = 15.7, 8.3 Hz, 1H), 2.35 (s, 3H).

^{13}C NMR (100 MHz, $CDCl_3$) δ = 154.09, 153.66, 138.80, 137.66, 129.16, 127.53, 125.69, 84.14, 55.91, 38.69, 21.04.

HRMS-ESI (m/z) calculated for $C_{16}H_{16}O_2Na$ $[M+Na]^+$ 263.1043, found 263.1053.

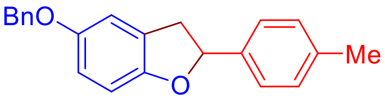

3f

5-(Benzyloxy)-2-(*p*-tolyl)-2,3-dihydrobenzofuran (3f) was prepared according to the general procedure using 4-benzyloxyphenol (40 mg, 0.2 mmol), 4-methylstyrene (29.2 µL, 0.22 mmol), ammonium persulfate (55 mg, 0.24 mmol, and **Hex-Aza-COF-3** (4.2 mg, 0.005 mmol). Product **3f** was obtained as a white solid (56.9 mg, 90% yield) after purification by flash chromatography.

^{1}H NMR (400 MHz, $CDCl_3$) δ = 7.45–7.36 (m, 4H), 7.31 (dd, *J* = 14.8, 7.6 Hz, 3H), 7.18 (d, *J* = 7.8 Hz, 2H), 6.85 (d, *J* = 2.2 Hz, 1H), 6.80–6.73 (m, 2H), 5.70 (t, *J* = 8.8 Hz, 1H), 5.01 (s, 2H), 3.56 (dd, *J* = 15.7, 9.3 Hz, 1H), 3.18 (dd, *J* = 15.7, 8.3 Hz, 1H), 2.35 (s, 3H).

^{13}C NMR (100 MHz, $CDCl_3$) δ = 154.15, 153.56, 139.04, 137.93, 137.54, 129.43, 128.69, 128.01, 127.81, 127.62, 125.96, 114.38, 112.47, 109.34, 84.44, 71.19, 38.94, 21.31.

HRMS-ESI (m/z) calculated for $C_{20}H_{20}O_2Na$ $[M+Na]^+$ 339.1367, found 339.1356.

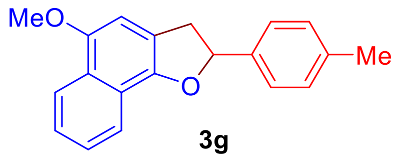

3g

5-Methoxy-2-(*p*-tolyl)-2,3-dihydronaphtho[1,2-b]furan (3g) was prepared according to the general procedure using 4-methoxynaphth-1-ol (35 mg, 0.2 mmol), 4-methylstyrene (29.2 µL, 0.22 mmol), ammonium persulfate (55 mg, 0.24 mmol), and **Hex-Aza-COF-3** (4.2 mg, 0.005 mmol). Product **3g** was obtained as a white solid (49.6 mg, 85% yield) after purification by flash chromatography.

^{1}H NMR (400 MHz, $CDCl_3$) δ = 8.25–8.19 (m, 1H), 7.99–7.96 (m, 1H), 7.51–7.43 (m, 2H), 7.36 (d, J = 8.0 Hz, 2H), 7.19 (d, J = 7.9 Hz, 2H), 6.74 (s, 1H), 5.91 (dd, J = 9.7, 7.9 Hz, 1H), 3.97 (s, 3H), 3.79 (dd, J = 15.5, 9.5 Hz, 1H), 3.36 (dd, J = 15.4, 7.9 Hz, 1H), 2.36 (s, 3H).
^{13}C NMR (100 MHz, $CDCl_3$) δ = 149.96, 148.31, 139.38, 137.51, 129.11, 125.85, 125.65, 125.20, 124.78, 122.29, 121.13, 120.62, 117.92, 101.42, 83.98, 55.86, 39.97, 21.00.
HRMS-ESI (m/z) calculated for $C_{20}H_{18}O_2Na$ $[M+Na]^+$ 313.1199, found 313.1246.

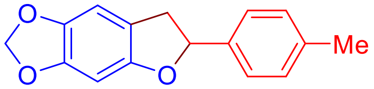

3h

6-(*p*-Tolyl)-6,7-dihydro-[1,3]dioxolo[4,5-f]benzofuran (3h) was prepared according to the general procedure using sesamol (27.6 mg, 0.2 mmol), 4-methylstyrene (29.2 µL, 0.22 mmol), ammonium persulfate (55 mg, 0.24 mmol), and **Hex-Aza-COF-3** (4.2 mg, 0.005 mmol). Product **3h** was obtained as an off-white solid (42.2 mg, 83% yield) after purification by flash chromatography.

^{1}H NMR (400 MHz, $CDCl_3$) δ = 7.31–7.25 (m, 3H), 7.18 (d, J = 7.8 Hz, 2H), 6.65 (s, 1H), 6.45 (s, 1H), 5.90 (d, J = 2.6 Hz, 2H), 5.71 (t, J = 8.8 Hz, 1H), 3.49 (dd, J = 15.2, 9.4 Hz, 1H), 3.11 (dd, J = 15.2, 8.3 Hz, 1H), 2.35 (s, 3H).
^{13}C NMR (100 MHz, $CDCl_3$) δ = 153.96, 147.09, 141.35, 138.63, 137.65, 129.11, 125.61, 117.09, 104.80, 100.91, 92.84, 84.76, 38.24, 20.99.
HRMS-ESI (m/z) calculated for $C_{16}H_{14}O_3Na$ $[M+Na]^+$ 277.0835, found 277.0883.

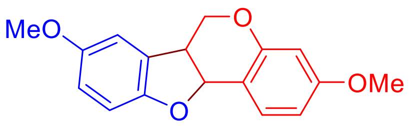

3i

3,8-Dimethoxy-6a,11a-dihydro-6H-benzofuro[3,2-c]chromene (3i) was prepared as per the general procedure using 4-methoxyphenol (25 mg, 0.2 mmol), 7-methoxy-*2H*-chromene (35.9 mg, 0.22 mmol), ammonium persulfate (55 mg, 0.24 mmol), and **Hex-Aza-COF-3** (4.2 mg, 0.005 mmol). Product **3i** was obtained as an off-white solid (52.1 mg, 91% yield) after purification by flash chromatography.

^{1}H NMR (400 MHz, $CDCl_3$) δ = 7.43 (d, *J* = 8.5 Hz, 1H), 6.85 (d, *J* = 2.6 Hz, 1H), 6.79–6.69 (m, 2H), 6.64 (dd, *J* = 8.5, 2.5 Hz, 1H), 6.48 (d, *J* = 2.5 Hz, 1H), 5.48 (d, *J* = 6.8 Hz, 1H), 4.28 (dd, *J* = 11.0, 5.1 Hz, 1H), 3.78 (d, *J* = 5.6 Hz, 6H), 3.70 (t, *J* = 11.0 Hz, 1H), 3.56 (ddd, *J* = 11.3, 6.8, 5.2 Hz, 1H).

^{13}C NMR (100 MHz, $CDCl_3$) δ = 161.22, 156.70, 154.52, 153.57, 132.04, 128.30, 114.04, 112.57, 111.30, 110.35, 109.39, 101.81, 78.04, 66.43, 56.21, 55.58, 40.80.

HRMS-ESI (m/z) calculated for $C_{17}H_{16}O_4Na$ $[M+Na]^+$ 307.0941, found 307.0983.

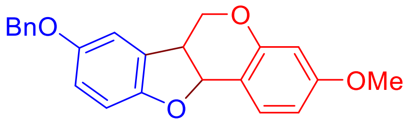

3j

8-(Benzyloxy)-3-methoxy-6a,11a-dihydro-6H-benzofuro[3,2-c]chromene (3j) was prepared as per the general procedure using 4-benzyloxyphenol (40 mg, 0.2 mmol), 7-methoxy-*2H*-chromene (35.9 mg, 0.22 mmol), ammonium persulfate (55 mg, 0.24 mmol), and **Hex-Aza-COF-3** (4.2 mg, 0.005 mmol). Product **3j** was obtained as a white solid (67.7 mg, 94% yield) after purification by flash chromatography. The NMR data are in full agreement with those previously published in the literature.[7]

^{1}H NMR (400 MHz, $CDCl_3$) δ = 7.40 (d, *J* = 8.5 Hz, 1H), 6.72 (s, 1H), 6.64 (dd, *J* = 8.6, 2.6 Hz, 1H), 6.47 (d, *J* = 2.6 Hz, 1H), 6.44 (s, 1H), 5.95–5.86 (m, 2H), 5.48 (d, *J* = 6.9 Hz, 1H), 4.23 (dd, *J* = 11.0, 5.0 Hz, 1H), 3.79 (s, 3H), 3.65 (t, *J* = 10.9 Hz, 1H), 3.48 (ddd, *J* = 11.3, 7.0, 5.1 Hz, 1H).

^{13}C NMR (100 MHz, $CDCl_3$) δ = 160.84, 156.38, 154.06, 147.89, 141.49, 131.55, 117.74, 112.14, 108.97, 104.53, 101.42, 101.09, 93.63, 78.30, 66.30, 55.18, 40.00.

HRMS-ESI (m/z) calculated for $C_{23}H_{20}O_4Na$ $[M+Na]^+$ 383.1254, found 383.1292.

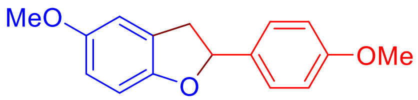

3k

5-Methoxy-2-(4-methoxyphenyl)-2,3-dihydrobenzofuran (3k) was prepared as per the general procedure using 4-methoxyphenol (25 mg, 0.2 mmol), 4-methoxystyrene (33 μL, 0.22 mmol), ammonium persulfate (55 mg, 0.24 mmol), and **Hex-Aza-COF-3** (4.2 mg, 0.005 mmol). Product **3k** was obtained as a white solid (46.4 mg, 90% yield) after purification by flash chromatography. The MNR data are in full agreement with those previously published in the literature.[8]

^{1}H NMR (500 MHz, $CDCl_3$) δ = 7.36–7.31 (m, 2H), 6.92–6.87 (m, 2H), 6.80–6.77 (m, 1H), 6.75 (d, J = 8.6 Hz, 1H), 6.69 (dd, J = 8.6, 2.7 Hz, 1H), 5.68 (t, J = 8.8 Hz, 1H), 3.81 (s, 3H), 3.77 (s, 3H), 3.55 (dd, J = 15.7, 9.2 Hz, 1H), 3.20 (dd, J = 15.7, 8.4 Hz, 1H).

^{13}C NMR (125 MHz, $CDCl_3$) δ 159.60, 154.37, 153.87, 134.06, 127.86, 127.45, 114.15, 113.11, 111.32, 109.32, 84.33, 56.19, 55.46, 38.86.

HRMS-ESI (m/z) calculated for $C_{16}H_{16}O_3Na$ $[M+Na]^+$ 279.0992, found 279.1045.

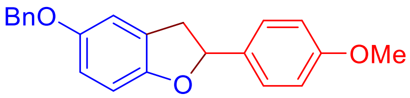

3l

5-(Benzyloxy)-2-(4-methoxyphenyl)-2,3-dihydrobenzofuran (3l) was prepared as per the general procedure using 4-benzyloxyphenol (40 mg, 0.2 mmol), 4-methoxystyrene (33 μL, 0.22 mmol), ammonium persulfate (55 mg, 0.24 mmol), and **Hex-Aza-COF-3** (4.2 mg, 0.005 mmol). Product **3l** was obtained as a white solid (58.6 mg, 91% yield) after purification by flash chromatography.

^{1}H NMR (500 MHz, $CDCl_3$) δ = 7.44 (d, J = 7.2 Hz, 2H), 7.40 (t, J = 7.5 Hz, 2H), 7.37–7.29 (m, 3H), 6.93–6.89 (m, 2H), 6.87 (d, J = 2.5 Hz, 1H), 6.81–6.73 (m, 2H), 5.69 (t, J = 8.8 Hz, 1H), 5.02 (s, 2H), 3.82 (s, 3H), 3.55 (dd, J = 15.7, 9.2 Hz, 1H), 3.20 (dd, J = 15.8, 8.4 Hz, 1H).

^{13}C NMR (125 MHz, $CDCl_3$) δ 159.34, 153.83, 153.30, 137.29, 133.76, 128.42, 127.74, 127.60, 127.35, 127.19, 114.14, 113.88, 112.21, 109.07, 84.09, 70.94, 55.19, 38.57.

HRMS-ESI (m/z) calculated for $C_{22}H_{20}O_3Na$ $[M+Na]^+$ 355.1305, found 355.1343.

9. Gram-Scale Synthesis of 2,3-Dihydrobenzofuran Derivative 3b

Compound **3b** was prepared as per the general procedure using 4-benzyloxyphenol (0.61 g, 3.05 mmol), *trans*-anethole (500 µL, 3.35 mmol), ammonium persulfate (1.05 g, 3.64 mmol), and **Hex-Aza-COF-3** (63 mg, 0.07 mmol). Product **3b** was obtained as a white solid (1.01 g, 96% yield) after purification by flash chromatography. The same NMR data as described above were obtained. 2,3-Dihydrobenzofuran derivative **3b** was then converted to (±)-conocarpan.

Section S4: Application of Hex-Aza-COF-3 Photocatalysis to Natural Product Synthesis

1. Synthesis of (±)-Conocarpan

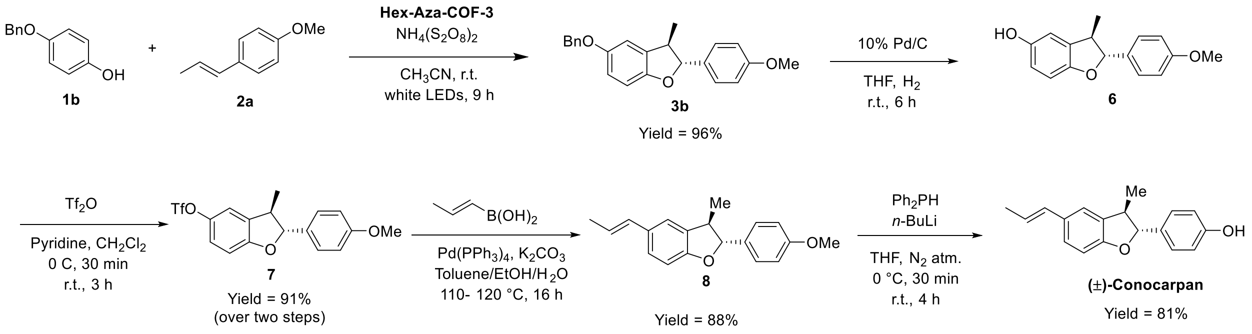

Scheme S4: Synthesis of **(±)-conocarpan**

2-(4-Methoxyphenyl)-3-methyl-2,3-dihydrobenzofuran-5-ol (6): To a solution of compound **3b** (0.4g, 1.15 mmol) in THF (25 mL), 10% Pd/C (40 mg) was added. The reaction mixture was bubbled with H_2 gas using a balloon equipped with a syringe needle and stirred at room temperature in H_2 atmosphere for 6 h. The reaction mixture was filtered over Celite and the filtrate was concentrated under reduced pressure and dried at 45 ˚C in a vacuum oven. The dried crude product (0.296 g, quantitative yield) was used for the next step without further purification.

2-(4-Methoxyphenyl)-3-methyl-2,3-dihydrobenzofuran-5-yl trifluoromethanesulfonate (7): The crude compound **6** (0.296 g, 1.15 mmol) was placed in a round bottom flask equipped with a stir bar, and CH_2Cl_2 (20 mL) was then added. The solution was cooled to 0 ˚C in an ice bath and then flushed with N_2 gas. Subsequently, pyridine (500 µL, 6.3 mmol) was added in one portion. Triflic anhydride (391 µL, 2.32 mmol) was then added dropwise over a period of 10 min via syringe at 0 ˚C. After stirring at 0 ˚C for 10 min, the mixture was allowed to warm to room temperature

and stirred for an additional 3 h at this temperature. The reaction was quenched by adding 10 mL of 1 M HCl, and the resulting mixture was transferred to a separation funnel and diluted with CH_2Cl_2 (25 mL). The organic layer was separated from the aqueous layer, which was extracted once with CH_2Cl_2 (25 mL), and the combined organic extracts were washed with sat. aq. $NaHCO_3$ (20 mL) and brine (20 mL), dried over anhydrous Na_2SO_4, and concentrated under reduced pressure. The crude product was purified by flash chromatography on a Biotage Snap Cartridge (KP-Sil 50 g) using a gradient solvent system (5% to 20% ethyl acetate in hexanes) to afford product **7** as a white solid (0.41 g, 91% yield over two steps). The spectroscopic data are in full agreement with those previously published in the literature.[4]

^{1}H NMR (400 MHz, $CDCl_3$) δ = 7.40–7.34 (m, 2H), 7.11–7.04 (m, 2H), 6.98–6.92 (m, 2H), 6.85 (d, *J* = 8.6 Hz, 1H), 5.21 (d, *J* = 9.2 Hz, 1H), 3.85 (s, 3H), 3.55–3.46 (m, 1H), 1.43 (d, *J* = 6.8 Hz, 3H).

^{13}C NMR (101 MHz, $CDCl_3$) δ = 159.94, 158.66, 143.35, 134.27, 131.60, 127.69, 121.25, 117.16, 114.16, 110.12, 93.53, 55.36, 45.21, 17.46.

(4-Methoxyphenyl)-3-methyl-5-((E)-prop-1-en-1-yl)-2,3-dihydrobenzofuran (8): To a mixture of compound **7** (0.32g, 0.82 mmol), *trans*-1-propen-1-ylboronic acid (0.14g, 1.63 mmol) and K_2CO_3 (0.34g, 2.46 mmol), toluene (20 mL), ethanol (5 mL), and water (7.5 mL) was added. The reaction mixture was flushed with N_2 gas, and $Pd(PPh_3)_4$ was then added (0.09 g, 0.08 mmol). The reaction was stirred at 110–120 ˚C for 16 h under N_2 gas atmosphere and then quenched by adding 1 M HCl at room temperature. The resulting mixture was diluted with ethyl acetate (25 mL) and water (25 mL) and the organic layer was separated from the aqueous layer. The aqueous layer was extracted once with ethyl acetate (25 mL), and the combined organic extracts were washed with sat. aq. $NaHCO_3$ (20 mL) and brine (20 mL), dried over anhydrous Na_2SO_4, and concentrated under reduced pressure. The crude product was purified by flash chromatography on a Biotage Snap Cartridge (KP-Sil 50 g) using a gradient solvent system (5% to 20% ethyl acetate in hexanes) to afford product **8** as a colorless oil (0.2 g, 88% yield). The spectroscopic data are in full agreement with those previously published in the literature.[4]

^{1}H NMR (400 MHz, $CDCl_3$) δ = 7.38–7.30 (m, 2H), 7.13 (dd, *J* = 10.7, 2.1 Hz, 2H), 6.91 (d, *J* = 8.7 Hz, 2H), 6.76 (s, 1H), 6.37 (dd, *J* = 15.7, 1.8 Hz, 1H), 6.09 (dq, *J* = 15.7, 6.6 Hz, 1H), 5.10 (d, *J* = 8.9 Hz, 1H), 3.82 (s, 3H), 3.47–3.33 (m, 1H), 1.86 (dd, *J* = 6.6, 1.7 Hz, 3H), 1.40 (d, *J* = 6.8 Hz, 3H).

^{13}C NMR (101 MHz, $CDCl_3$) δ = 159.67, 158.35, 132.68, 132.41, 131.24, 130.77, 127.66, 126.31, 123.03, 120.73, 114.03, 109.29, 92.67, 55.34, 45.22, 18.43, 17.81.

(±)-Conocarpan: To a solution of diphenylphosphine (330 µL, 1.91 mmol) in THF (10 mL) *n*-butyllithium (2.5 M in hexane, 940 µL) was added at 0 ˚C dropwise over a period of 5 min under N_2 gas atmosphere. The solution was stirred at 0 ˚C for 30 min, and compound **8** (0.15 g, 0.54 mmol) was added in THF (1 mL) via syringe. The reaction

mixture was warmed to room temperature and stirred for 4 h. Then, 2 M NaOH (10 mL) was added to the reaction and the solution was washed with hexane (3 × 25 mL). The basic aqueous layer was cooled in an ice bath, acidified with 4 M HCl (pH = 3–4), and then extracted with CH_2Cl_2 (3 × 25 mL). The combined organic extracts were dried over anhydrous Na_2SO_4 and concentrated under reduced pressure. The crude product was purified by flash chromatography on a Biotage Snap Cartridge (KP-Sil 50 g) using a gradient solvent system (5% to 40% ethyl acetate in hexanes) to afford **(±)-conocarpan** as a colorless oil (0.31 g, 81% yield). The spectroscopic data are in full agreement with those previously published in the literature.[9]

^{1}H NMR (400 MHz, $CDCl_3$) δ = 7.32–7.28 (m, 2H), 7.16–7.09 (m, 2H), 6.86–6.82 (m, 2H), 6.76 (d, *J* = 8.1 Hz, 1H), 6.37 (dd, *J* = 15.7, 1.9 Hz, 1H), 6.09 (dq, *J* = 15.7, 6.6 Hz, 1H), 5.08 (d, *J* = 8.9 Hz, 1H), 4.80 (s, 1H), 3.39 (p, *J* = 7.1 Hz, 1H), 1.90–1.83 (m, 3H), 1.39 (d, *J* = 6.8 Hz, 3H).

^{13}C NMR (100 MHz, $CDCl_3$) δ = 158.74, 156.03, 133.40, 132.81, 131.72, 131.19, 128.33, 126.76, 123.51, 121.18, 115.90, 109.74, 93.05, 45.69, 18.88, 18.29.

2. Synthesis of (±)-Pterocarpin

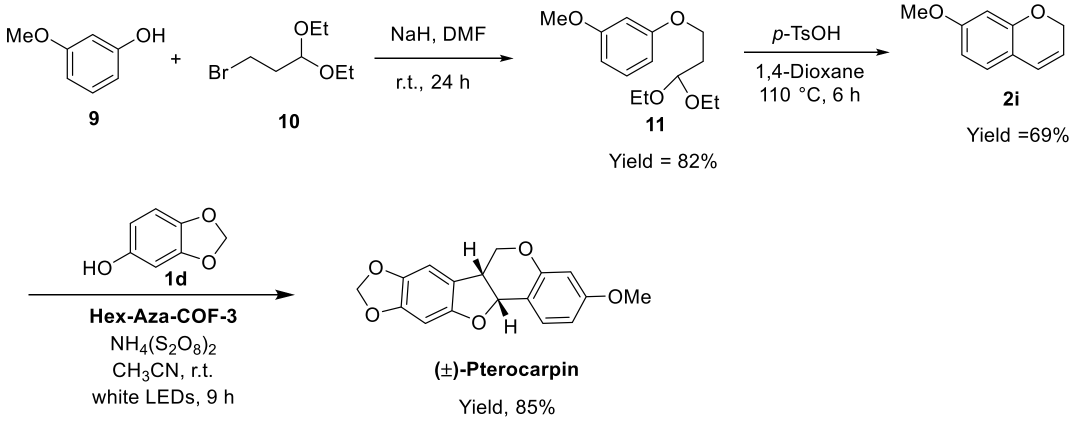

Scheme S5: Synthesis of **(±)-pterocarpin**

1-(3,3-Diethoxypropoxy)-3-methoxybenzene (11): To a solution of 3-methoxyphenol (1.51 g, 12 mmol) in DMF (20 mL), NaH (0.97 g of a 60% suspension in oil, 24 mmol) was added at 0 ˚C in an ice bath. The mixture was stirred at room temperature for 30 min, and 3-bromo-1,1-diethoxypropane (2.57 g, 12 mmol) was then added. The reaction mixture was heated at 80 ˚C for 16 h and then cooled to room temperature and neutralized with acetic acid. The neutral solution was extracted with Et_2O (3 × 30 mL), dried over anhydrous Na_2SO_4, and concentrated under reduced pressure. The crude product was purified by flash chromatography on a Biotage Snap Cartridge (KP-Sil 50 g) using a

gradient solvent system (2%–30% ethyl acetate in hexanes) to afford product **11** as a clear oil (2.54 g 82% yield). The spectroscopic data are in full agreement with those previously published in the literature.[10]

^{1}H NMR (400 MHz, $CDCl_3$) δ = 7.17 (t, *J* = 8.2 Hz, 1H), 6.53–6.44 (m, 3H), 4.75 (t, *J* = 5.7 Hz, 1H), 4.04 (t, *J* = 6.2 Hz, 2H), 3.79 (s, 3H), 3.74–3.66 (m, 2H), 3.53 (dq, *J* = 9.4, 7.0 Hz, 2H), 2.09 (q, *J* = 6.1 Hz, 2H), 1.21 (t, *J* = 7.0 Hz, 6H).

^{13}C NMR (101 MHz, $CDCl_3$) δ = 160.83, 160.19, 129.85, 106.75, 106.25, 100.98, 100.45, 64.10, 61.84, 55.27, 33.87, 15.36.

7-Methoxy-*2H*-chromene (2i): To a solution of 1-(3,3-diethoxypropoxy)-3-methoxybenzene (1.21 g, 4.76 mmol) in 1,4-dioxane (15 mL), *p*-toluenesulfonic acid monohydrate (0.09 g, 0.47 mmol) was added, and the mixture was stirred at 110 ˚C for 5 h. After cooling to room temperature, the reaction mixture was diluted with Et_2O and washed with 10% aq. NaOH (2 × 25 mL), water (25 mL), and brine (25 mL). The organic layer was dried over anhydrous Na_2SO_4 and concentrated under reduced pressure. The crude product was purified by flash chromatography on a Biotage Snap Cartridge (KP-Sil 50 g) using a gradient solvent system (2% to 30% ethyl acetate in hexanes) to afford product **2i** as a clear oil (0.53 g 69% yield). The spectroscopic data are in full agreement with those previously published in the literature.[10]

^{1}H NMR (400 MHz, $CDCl_3$) δ = 6.87 (d, *J* = 8.3 Hz, 1H), 6.42 (dd, *J* = 8.3, 2.5 Hz, 1H), 6.40–6.35 (m, 2H), 5.63 (dt, *J* = 9.8, 3.6 Hz, 1H), 4.79 (dd, *J* = 3.6, 1.8 Hz, 2H), 3.77 (s, 3H).

^{13}C NMR (101 MHz, $CDCl_3$) δ = 160.55, 155.31, 127.24, 124.24, 118.87, 115.70, 106.93, 101.72, 65.62, 55.35.

(±)-Pterocarpin: This compound was prepared as per the general procedure using sesamol (27.6 mg, 0.2 mmol), 7-methoxy-*2H*-chromene (35.9 mg, 0.22 mmol), ammonium persulfate (55 mg, 0.24 mmol), and **Hex-Aza-COF-3** (4.2 mg, 0.005 mmol). **(±)-Pterocarpin** was obtained as an off-white solid (50.8 mg, 85% yield) after purification by flash chromatography. The NMR data are in full agreement with those previously published in the literature.[7]

^{1}H NMR (400 MHz, $CDCl_3$) δ = 7.45–7.32 (m, 6H), 6.92 (d, *J* = 2.5 Hz, 1H), 6.85–6.70 (m, 2H), 6.64 (dd, *J* = 8.5, 2.6 Hz, 1H), 6.48 (d, *J* = 2.5 Hz, 1H), 5.49 (d, *J* = 6.8 Hz, 1H), 5.01 (s, 2H), 4.27 (dd, *J* = 11.0, 5.0 Hz, 1H), 3.79 (s, 3H), 3.70 (t, *J* = 10.9 Hz, 1H), 3.56 (ddd, *J* = 11.3, 6.8, 5.1 Hz, 1H).

^{13}C NMR (100 MHz, $CDCl_3$) δ = 161.17, 156.65, 153.75, 153.65, 137.32, 131.98, 128.72, 128.24, 128.11, 127.67, 115.21, 112.49, 112.42, 110.30, 109.34, 101.75, 78.01, 71.20, 66.36, 55.52, 40.72.

HRMS-ESI (m/z) calculated for $C_{17}H_{14}O_5Na$ $[M+Na]^+$ 321.0792, found 321.0780.

Section S5: Photocatalyst Recyclability Studies

The photocatalytic reaction was performed according to the general procedure using 4-methoxyphenol (150 mg, 1.2 mmol), *trans*-anethole (198 µL, 1.32 mmol), ammonium persulfate (330 mg, 1.44 mmol), and **Hex-Aza-COF-3** (25.2 mg, 0.015 mmol) in CH_3CN (15 mL). On completion of the reaction, **Hex-Aza-COF-3** was recovered by centrifugation and washed with CH_3CN (10 mL), water (10 mL), THF (10 mL), and acetone (10 mL), dried at room temperature under vacuum overnight, and used for the next cycle. **Hex-Aza-COF-3** was used up to five consecutive cycles and after each cycle, product **3a** was isolated and purified by flash chromatography on a Biotage Snap Cartridge (KP-Sil 25 g) using a gradient solvent system (5% to 20% ethyl acetate in hexanes). The results of this study are summarized in **Figure S6**.

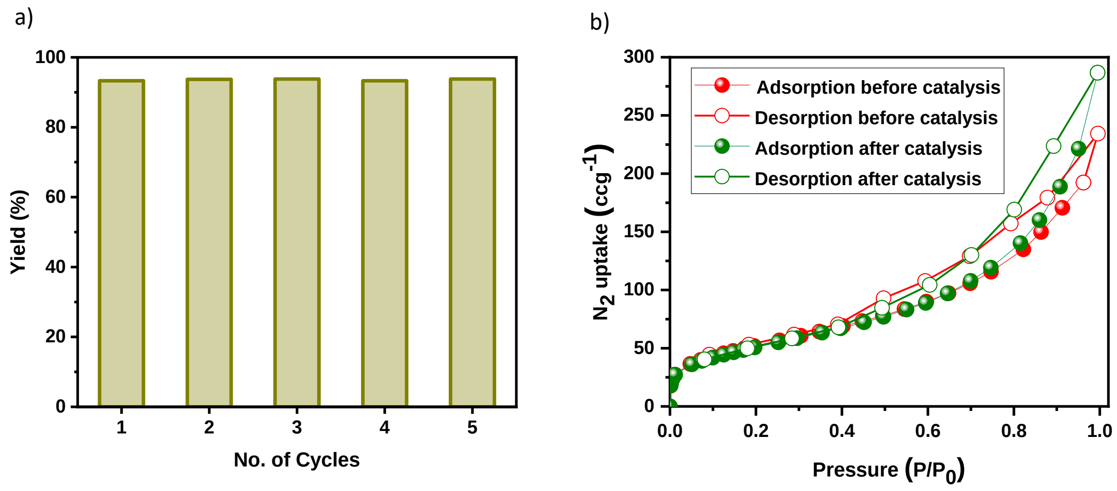

Figure S6: a) Recycling test of **Hex-Aza-COF-3**; b) N_2 adsorption isotherm of **Hex-Aza-COF-3** before and after the catalysis.

Section S6: Stern–Volmer Analysis

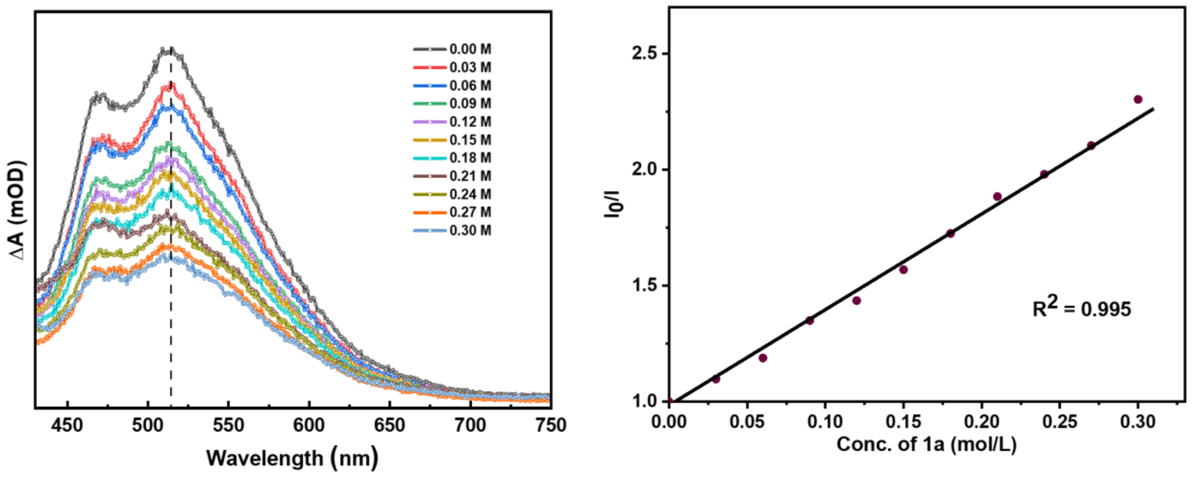

Figure S7: Steady-state emission quenching of **Hex-Aza-COF-3** with substrate **1a** (Stern–Volmer analysis)

Section S7: µs-Transient Absorption measurement

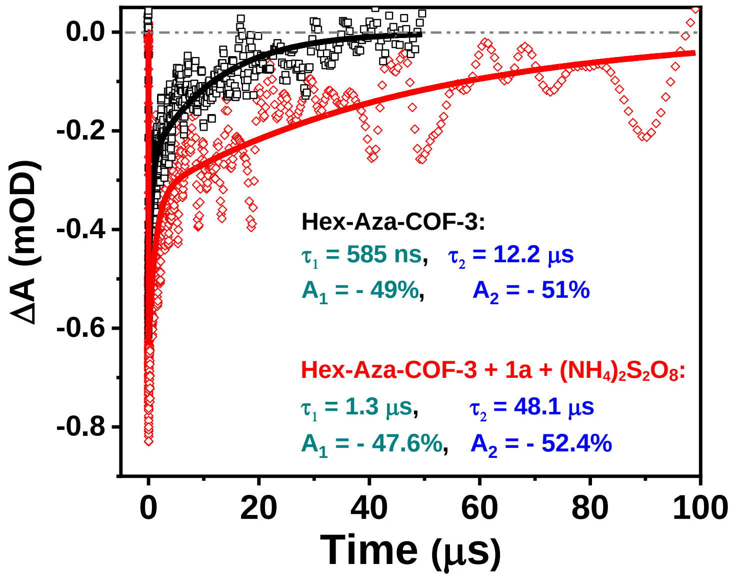

Figure S8: µs-Transient Absorption measurement. Kinetic traces and associated time constants for **Hex-Aza-COF-3** at 600 nm and **Hex-Aza-COF-3** with **1a** and $(NH_4)_2S_2O_8$ at 600 nm. λ_{exc} = 400 nm

Section S8: Photocatalysis using Sunlight as the Light Source

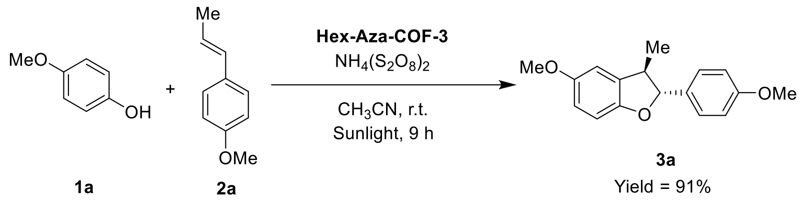

Scheme S6: Photocatalysis using sunlight

A 20 mL scintillation vial equipped with a stir bar was charged with **Hex-aza-COF-3** (4.2 mg, 0.005 mmol) and CH_3CN (5 mL). Subsequently, 4-methoxyphenol (25 mg, 0.2 mmol), *trans*-anethole (33 µL, 0.22 mmol), and ammonium persulfate (55 mg, 0.24 mmol) were added. The reaction mixture was sonicated for 15 min and then stirred at room temperature for 9 h under sunlight as the light source. The reaction mixture was transferred to a centrifuge tube and centrifuged at 5000 rpm for 5 min, and the liquid phase was separated. To the residue, CH_3CN (5 mL) was added, mixed thoroughly, centrifuged at 5000 rpm for 5 min, and the liquid phase was separated. The combined liquid phase was concentrated under reduced pressure and the crude product was purified by flash chromatography on a Biotage Snap Cartridge (KP-Sil 10 g) using a gradient solvent system (5%–20% ethyl acetate in hexanes) to afford product **3a**.

Section S9: References

(1) Bindewald, E.; Lorenz, R.; Hubner, O.; Brox, D.; Herten, D. P.; Kaifer, E.; Himmel, H. J. Tetraguanidino-functionalized phenazine and fluorene dyes: synthesis, optical properties and metal coordination. *Dalton. Trans.* **2015,** *44* (7), 3467-3485.

(2) Wu, W.-H. H., M.-J.; Zeng, Q.; Xian, W.-R.; Liao, W.-M.; He, J. Electrical and magnetic properties of a radical-based Co(II) coordination complex with CeH···π and π···π supramolecular interactions. *Inorg. Chem. Commun.* **2019,** *103*, 149-153.

(3) Kandambeth, S.; Jia, J.; Wu, H.; Kale, V. S.; Parvatkar, P. T.; Czaban-Jozwiak, J.; Zhou, S.; Xu, X.; Ameur, Z. O.; Abou-Hamad, E.; Emwas, A. H.; Shekhah, O.; Alshareef, H. N.; Eddaoudi, M. Covalent Organic Frameworks as Negative Electrodes for High-Performance Asymmetric Supercapacitors. *Adv. Energy Mater.* **2020,** *10* (38), 2001673.

(4) Blum, T. R.; Zhu, Y.; Nordeen, S. A.; Yoon, T. P. Photocatalytic Synthesis of Dihydrobenzofurans by Oxidative [3+2] Cycloaddition of Phenols. *Angew. Chem., Int. Ed.* **2014,** *53* (41), 11056-11059.

(5) Fischer, C.; Kerzig, C.; Zilate, B.; Wenger, O. S.; Sparr, C. Modulation of Acridinium Organophotoredox Catalysts Guided by Photophysical Studies. *ACS Catal.* **2020,** *10* (1), 210-215.

(6) Huang, Z.; Jin, L. Q.; Feng, Y.; Peng, P.; Yi, H.; Lei, A. Iron-Catalyzed Oxidative Radical Cross-Coupling/Cyclization between Phenols and Olefins. *Angew. Chem., Int. Ed.* **2013,** *52* (28), 7151-7155.

(7) Mohr, A. L.; Lombardo, V. M.; Arisco, T. M.; Morrow, G. W. Synthesis of Pterocarpan-Type Heterocycles via Oxidative Cycloadditions of Phenols and Electron-Rich Arenes. *Synth. Commun.* **2009,** *39* (21), 3845-3855.

(8) Jing, Z. R.; Liang, D. D.; Tian, J. M.; Zhang, F. M.; Tu, Y. Q. Enantioselective Construction of 2-Aryl-2,3-dihydrobenzofuran Scaffolds Using Cu/SPDO-Catalyzed [3+2] Cycloaddition. *Org. Lett.* **2021,** *23* (4), 1258-1262.

(9) Snider, B. B.; Han, L.; Xie, C. Synthesis of 2,3-dihydrobenzofurans by $Mn(OAc)_3$-based oxidative cycloaddition of 2-cyclohexenones with alkenes. Synthesis of (+/-)-conocarpan. *J. Org. Chem.* **1997,** *62* (20), 6978-6984.

(10) Stokes, S.; Mustain, R.; Pickle, L.; Mead, K. T. Rhodium-catalyzed cyclopropanations of 2-aryl-2H-chromenes with dialkyl malonate esters. A comparison of alpha-diazo derivatives and phenyliodonium ylides. *Tetrahedron Lett.* **2012,** *53* (30), 3890-3893.

Section S10: ^{1}H and ^{13}C NMR Spectra

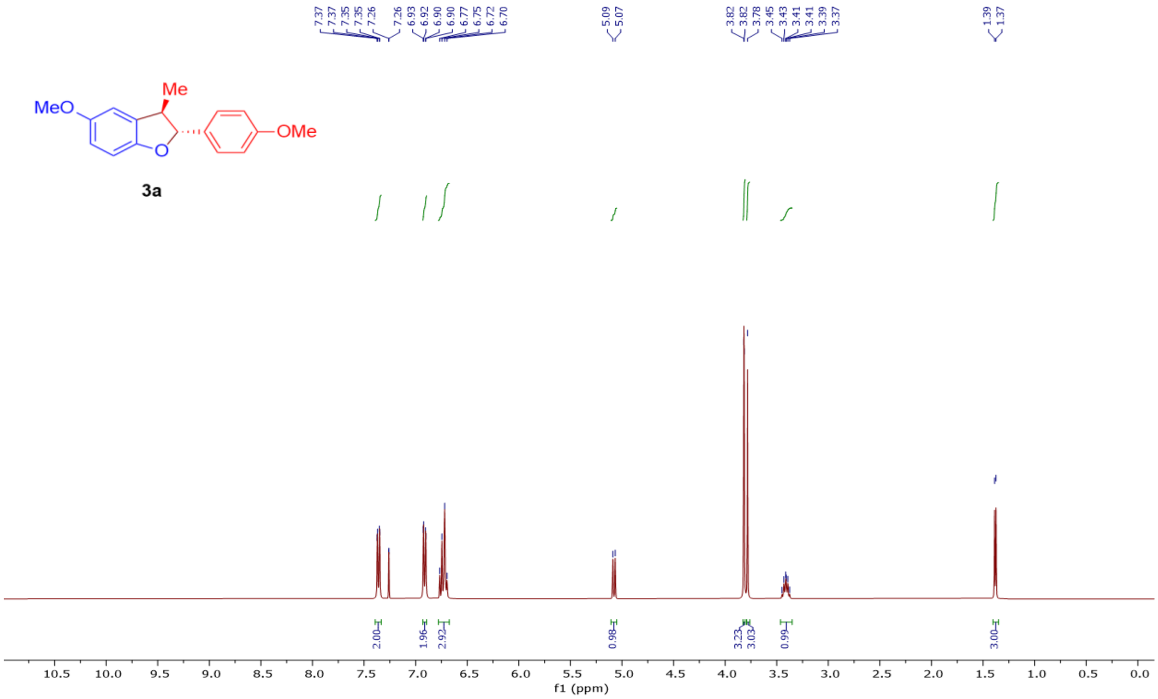

Figure S7-1: ^{1}H NMR spectrum of **3a** in $CDCl_3$

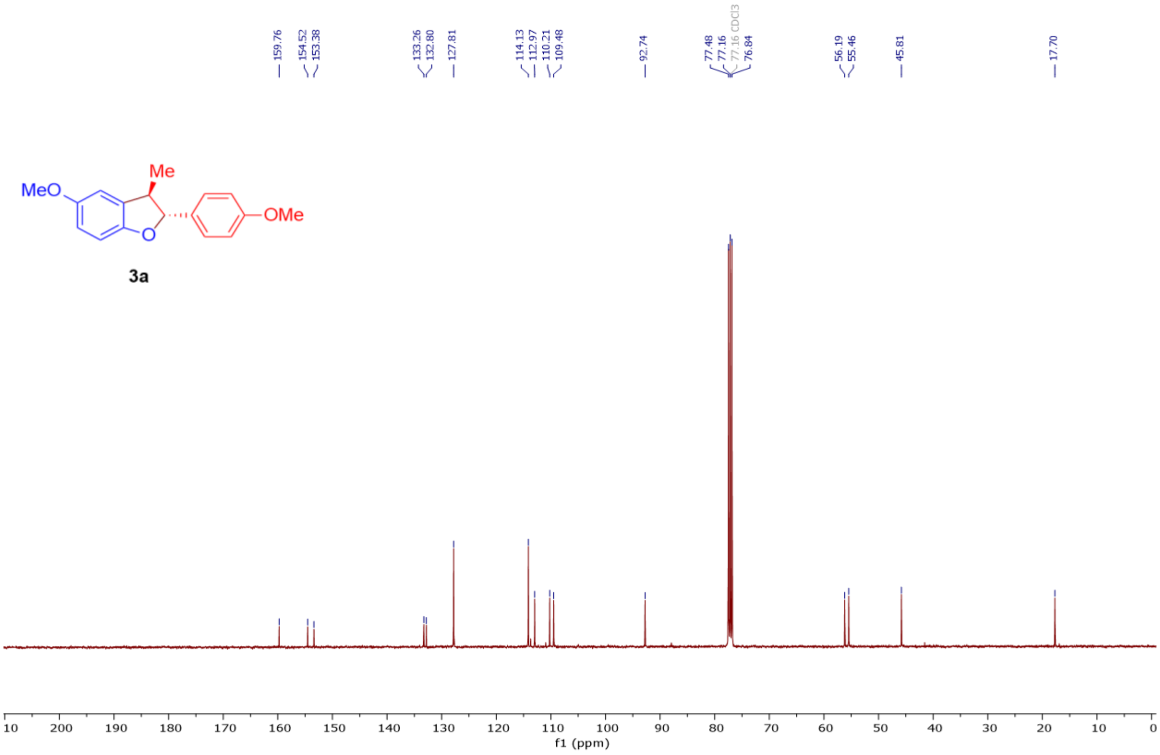

Figure S7-2: ^{13}C NMR spectrum of **3a** in $CDCl_3$

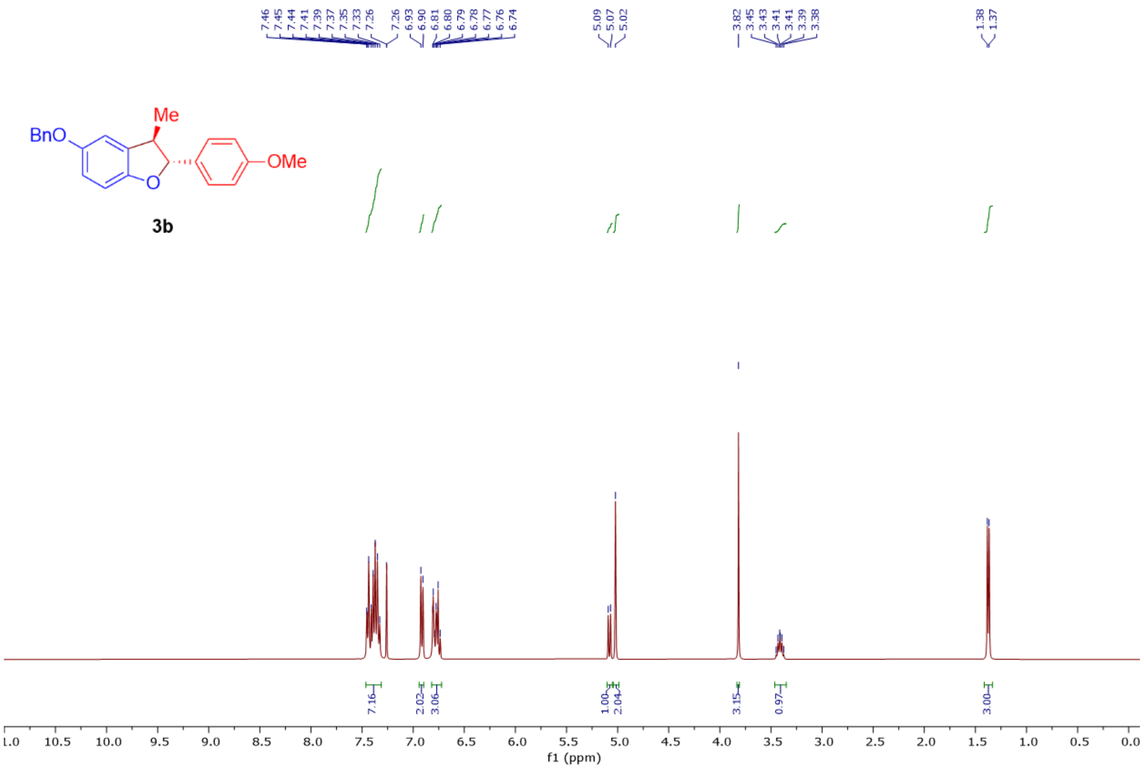

Figure S7-3: ^{1}H NMR spectrum of **3b** in $CDCl_3$

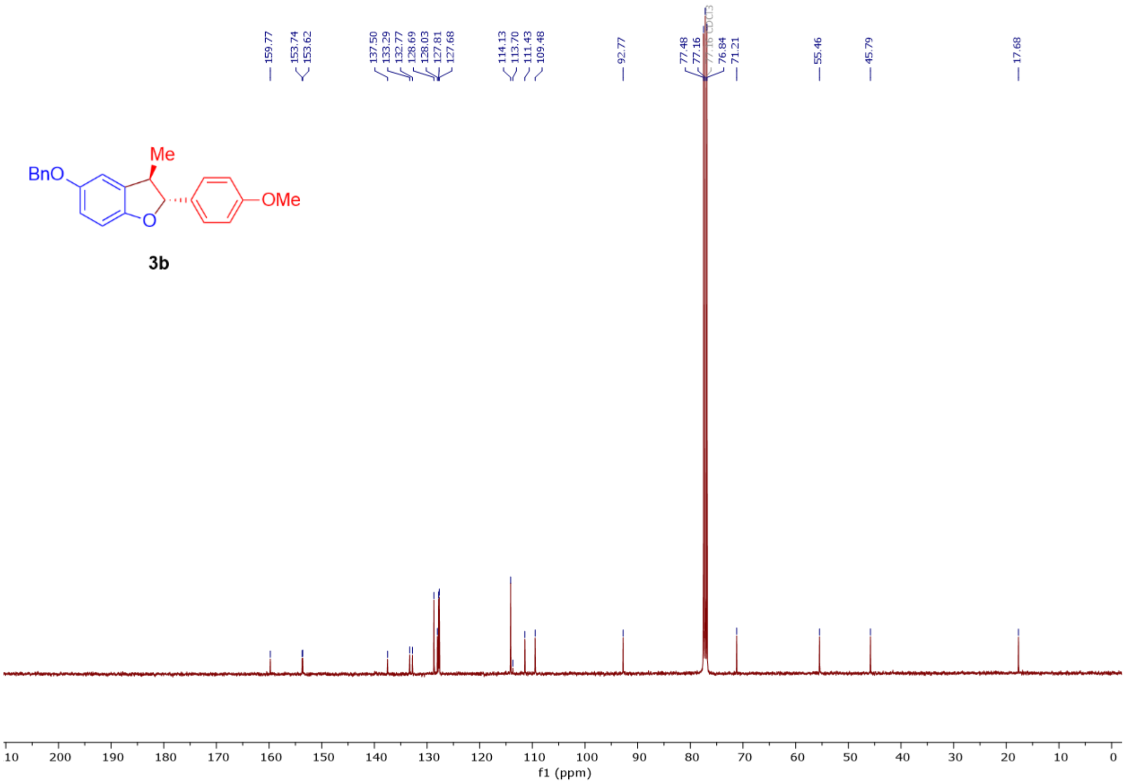

Figure S7-4: ^{13}C NMR spectrum of **3b** in $CDCl_3$

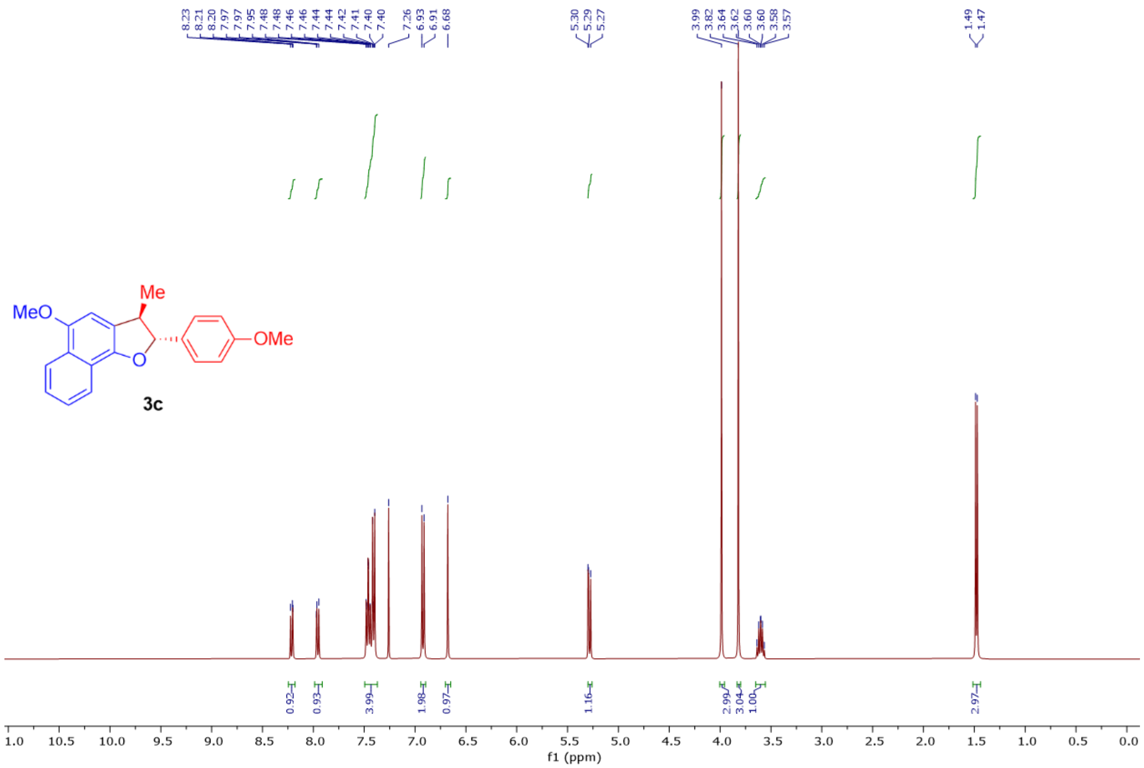

Figure S7-5: ^{1}H NMR spectrum of **3c** in $CDCl_3$

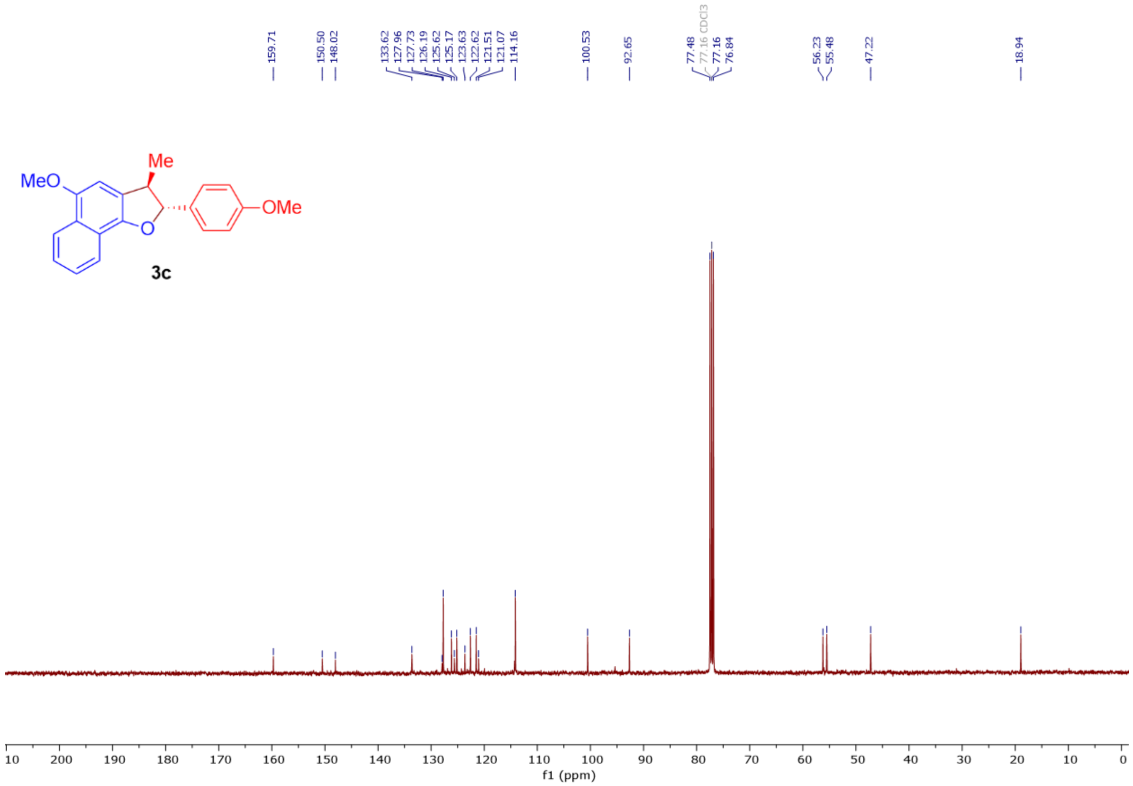

Figure S7-6: ^{13}C NMR spectrum of **3c** in $CDCl_3$

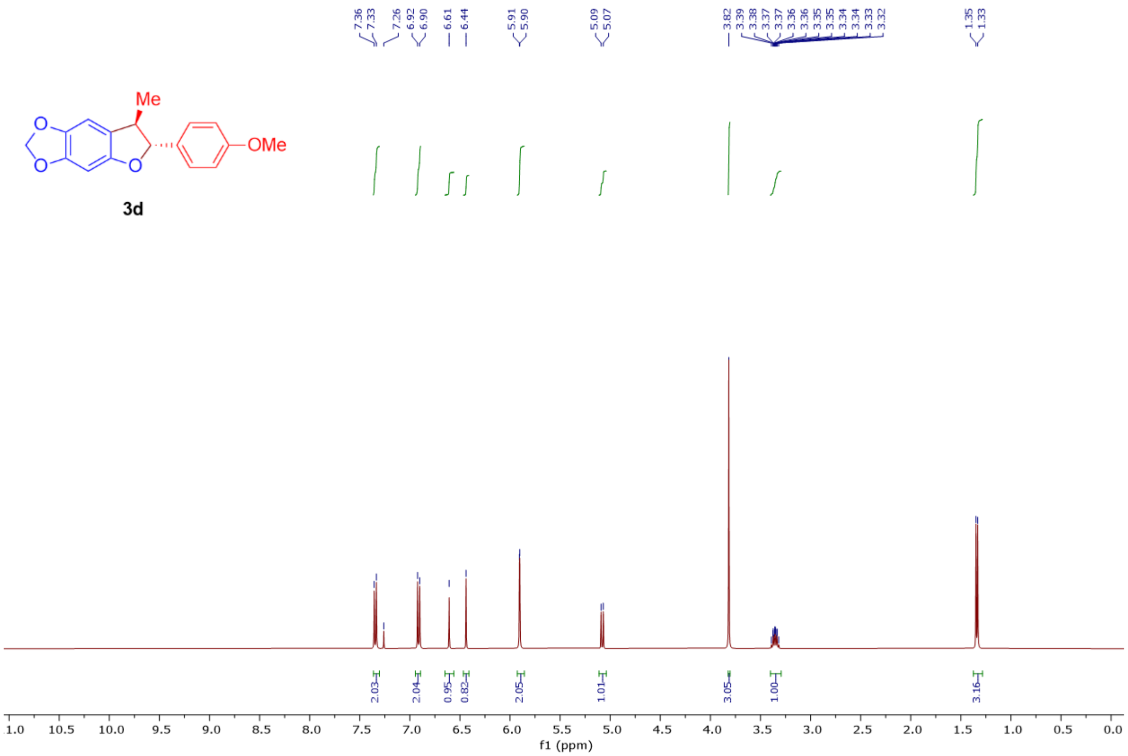

Figure S7-7: ^{1}H NMR spectrum of **3d** in $CDCl_3$

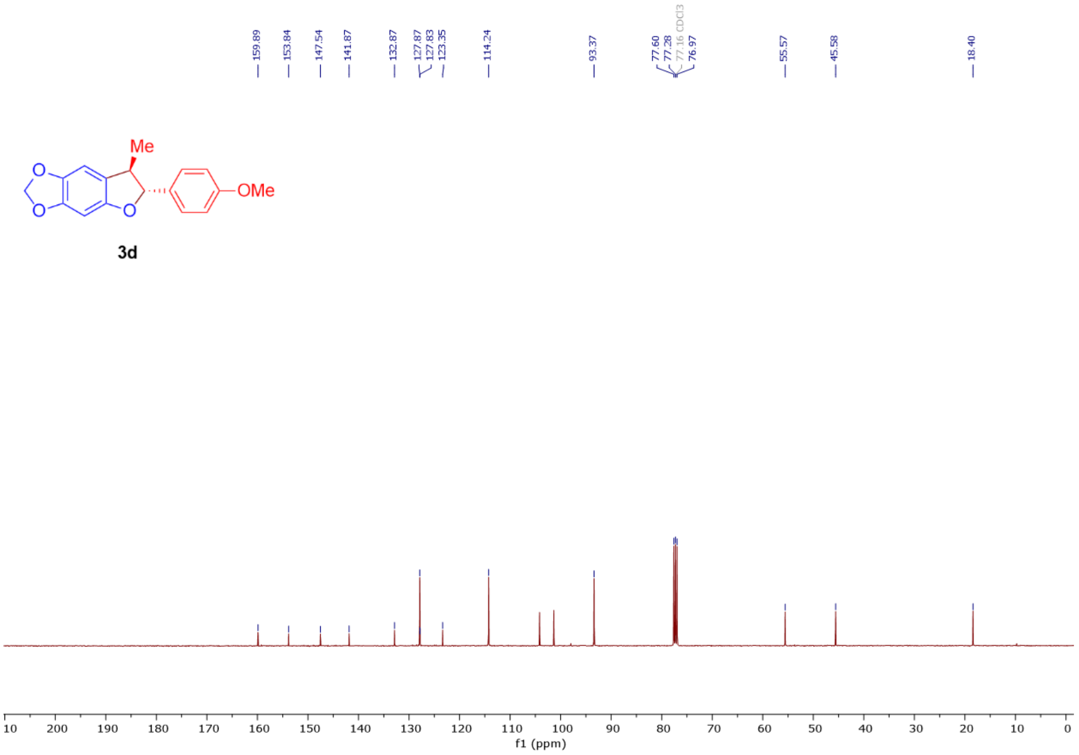

Figure S7-8: ^{13}C NMR spectrum of **3d** in $CDCl_3$

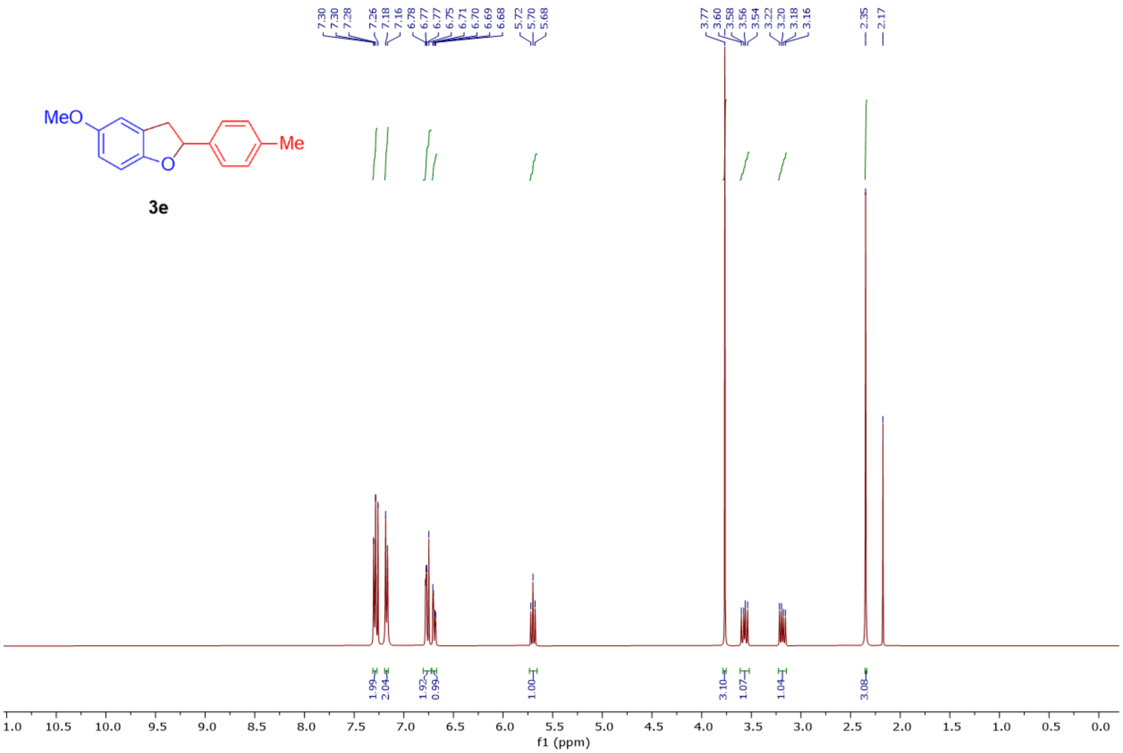

3e

Figure S7-9: ^{1}H NMR spectrum of **3e** in $CDCl_3$

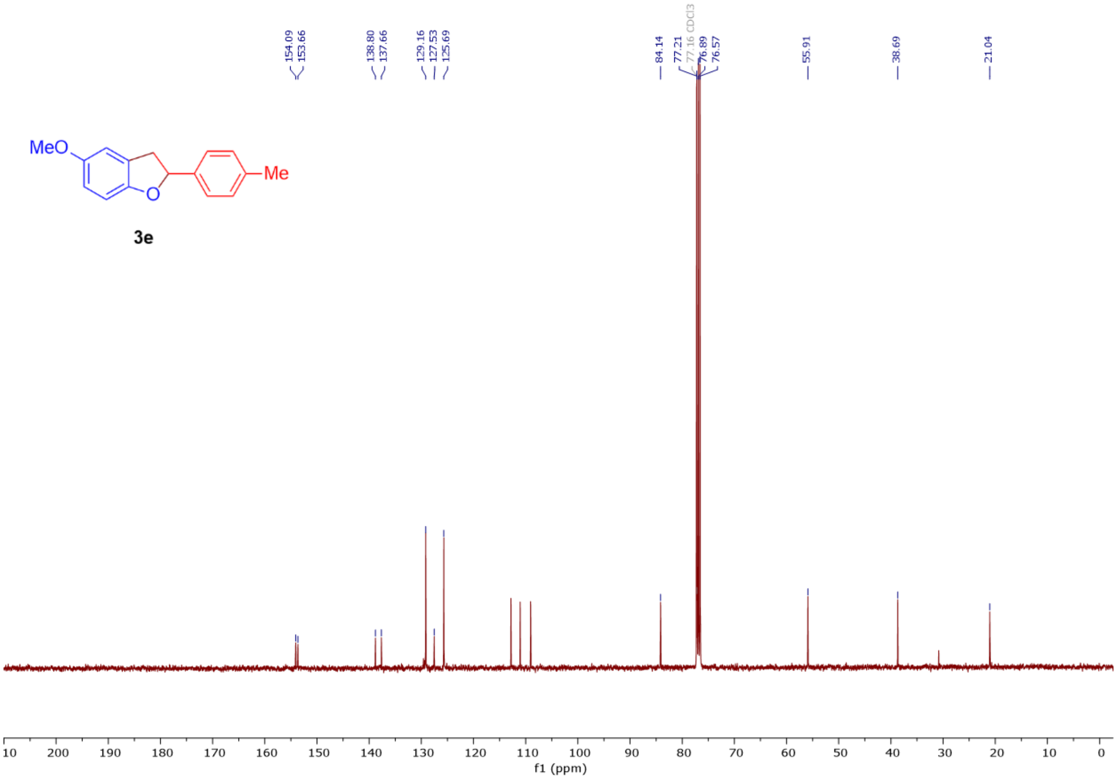

3e

Figure S7-10: ^{13}C NMR spectrum of **3e** in $CDCl_3$

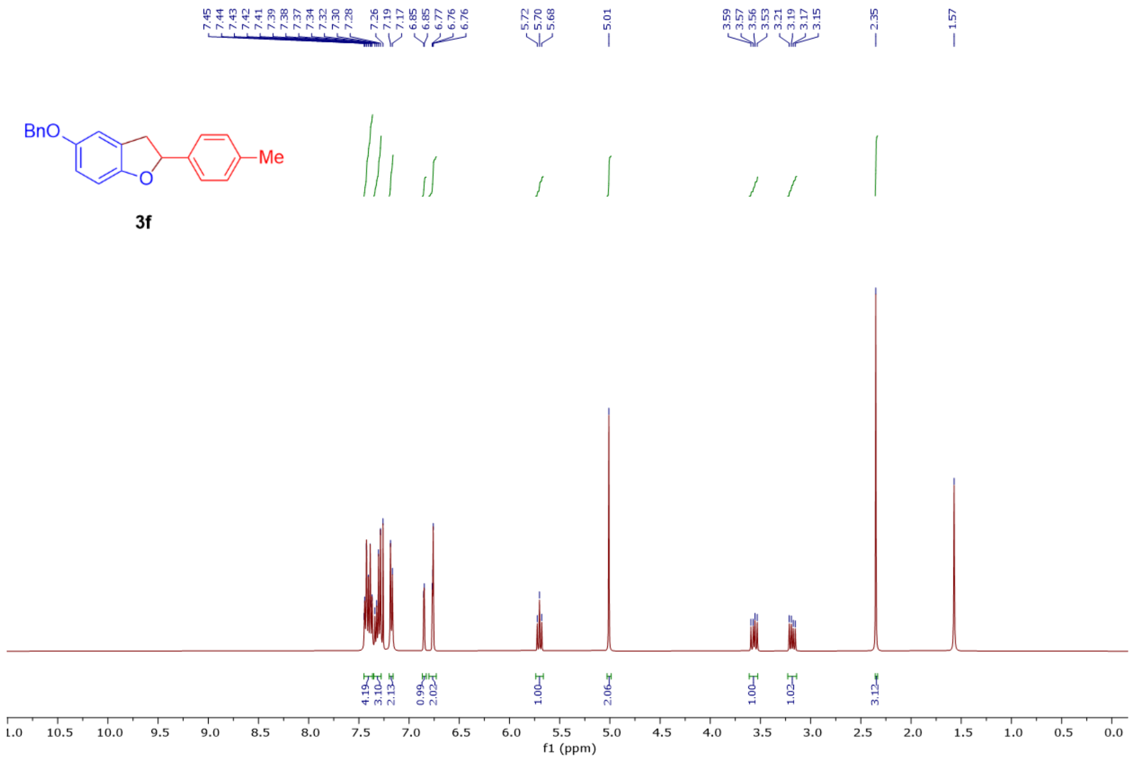

Figure S7-11: ^{1}H NMR spectrum of **3f** in $CDCl_3$

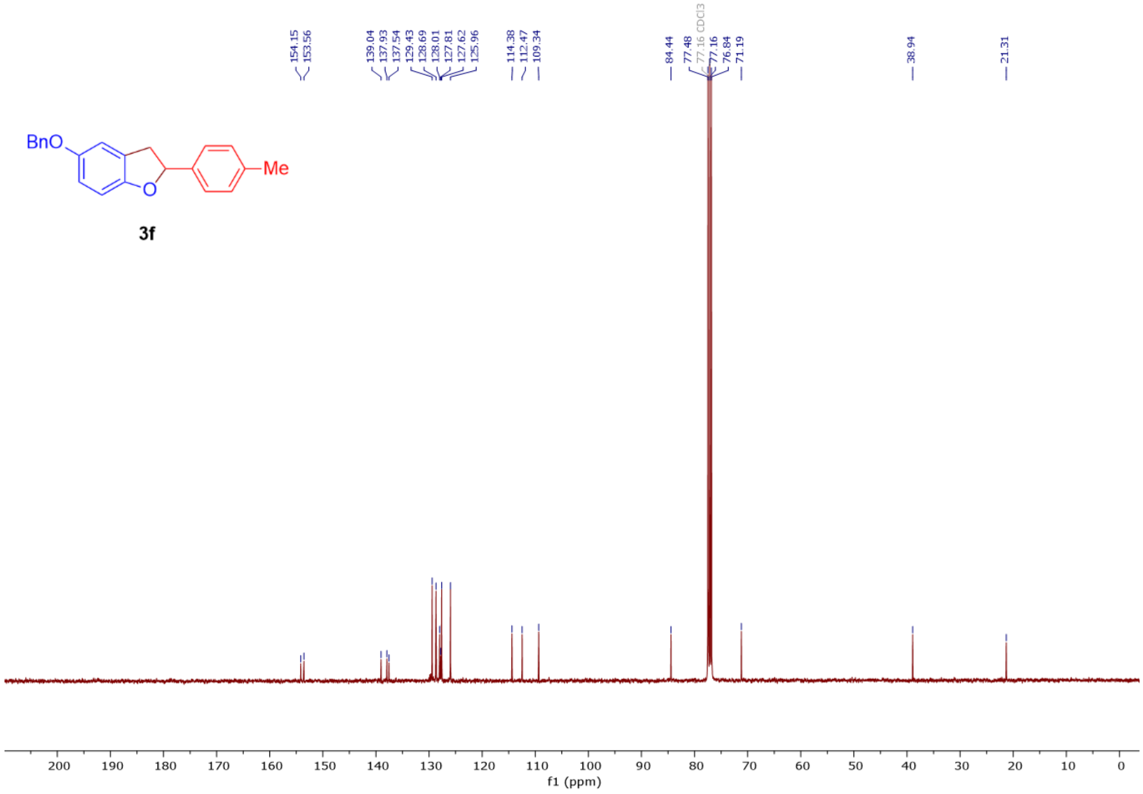

Figure S7-12: ^{13}C NMR spectrum of **3f** in $CDCl_3$

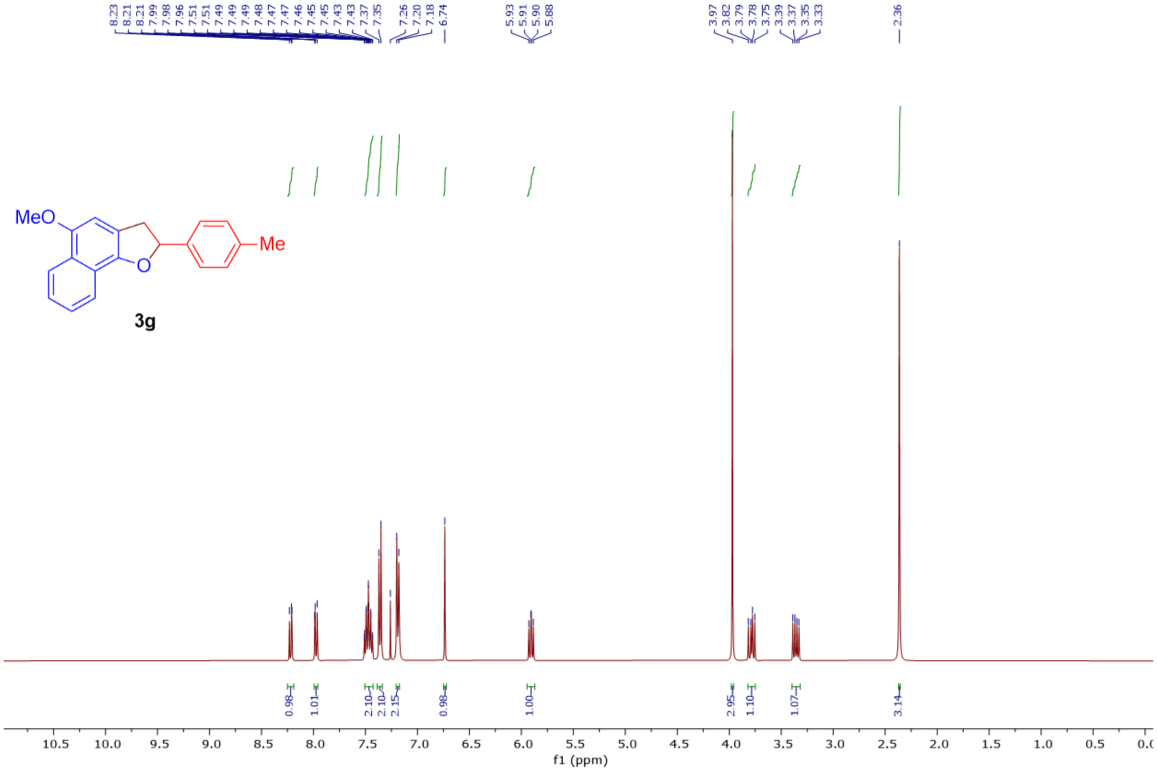

Figure S7-13: ^{1}H NMR spectrum of **3g** in $CDCl_3$

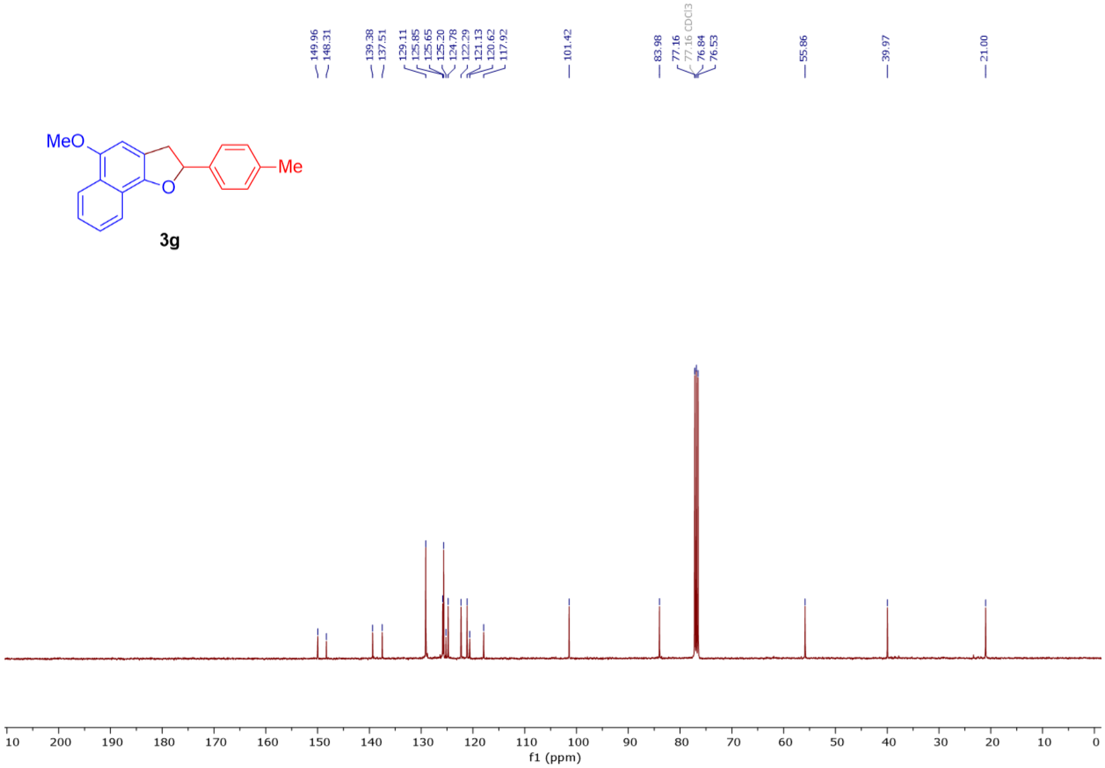

Figure S7-14: ^{13}C NMR spectrum of **3g** in $CDCl_3$

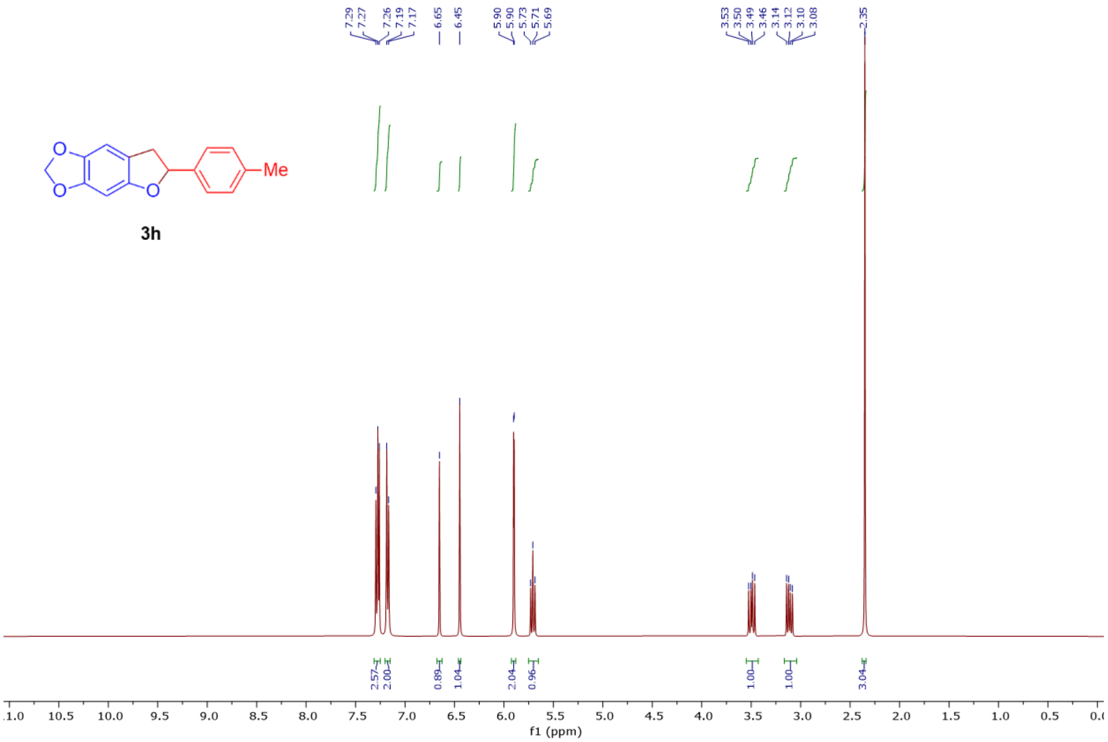

Figure S7-15: ^{1}H NMR spectrum of **3h** in $CDCl_3$

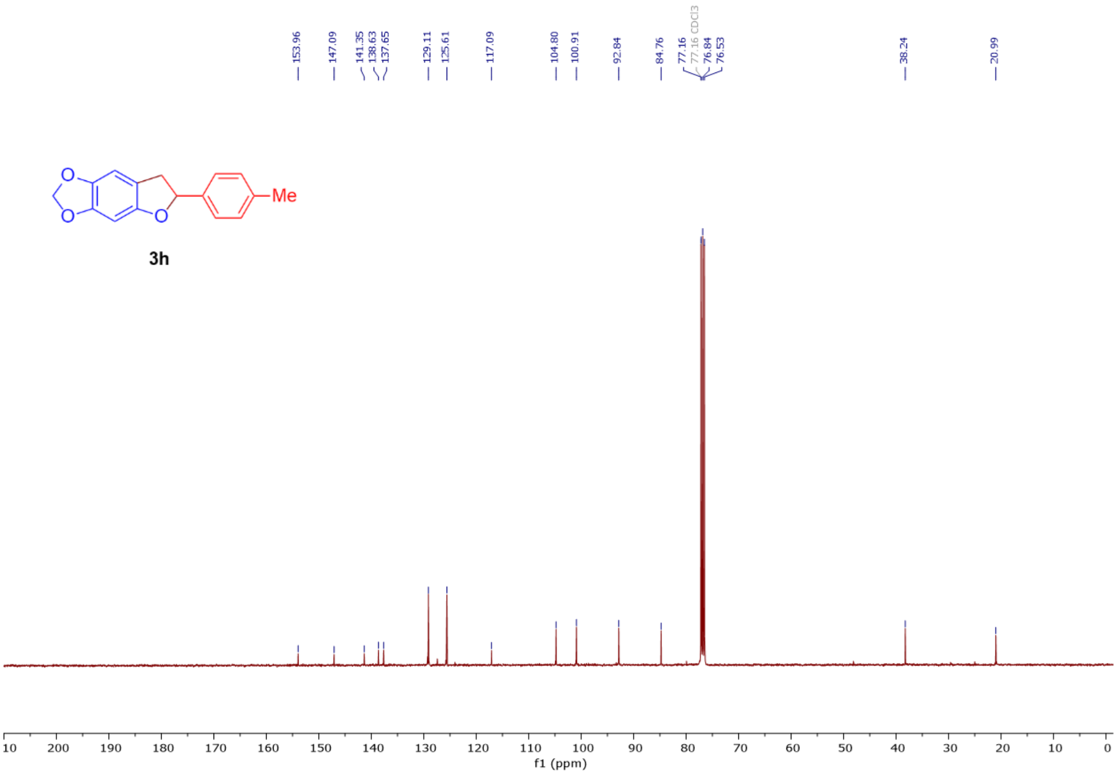

Figure S7-16: ^{13}C NMR spectrum of **3h** in $CDCl_3$

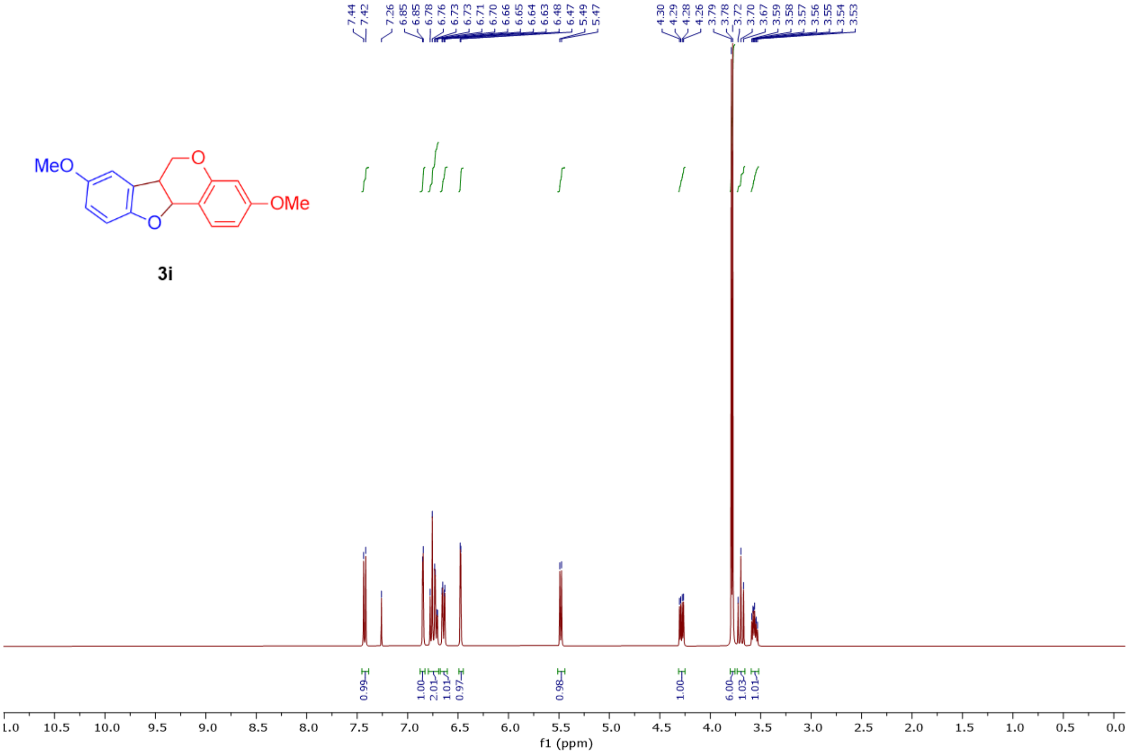

Figure S7-17: ^{1}H NMR spectrum of **3i** in $CDCl_3$

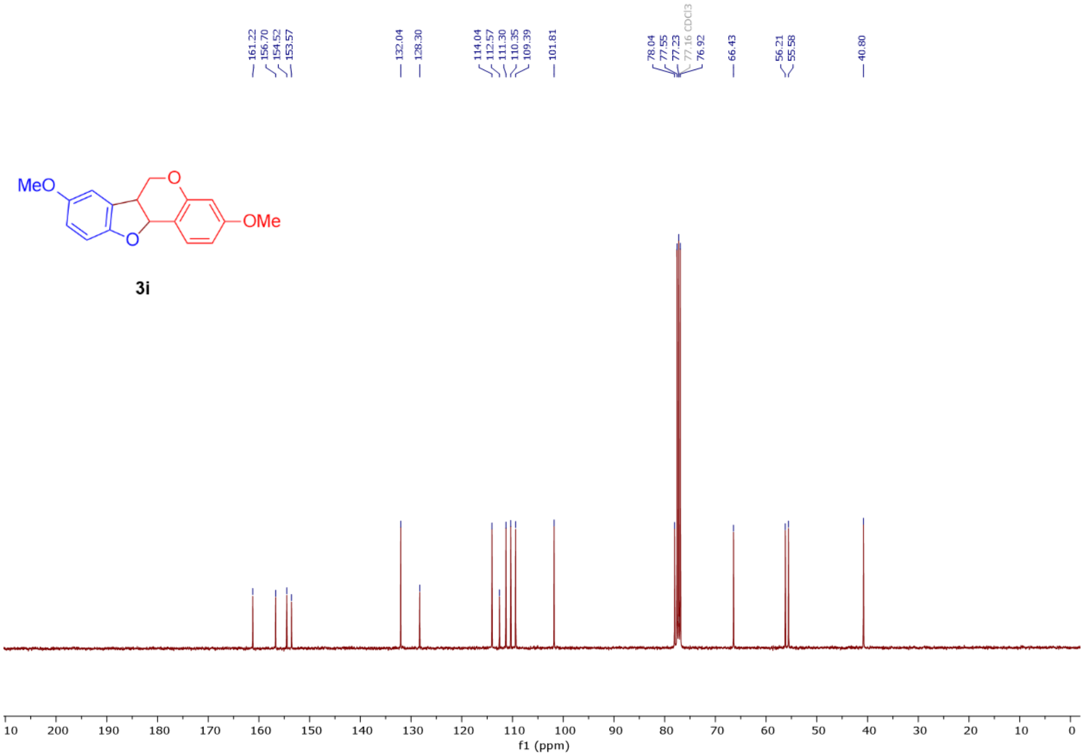

Figure S7-18: ^{13}C NMR spectrum of **3i** in $CDCl_3$

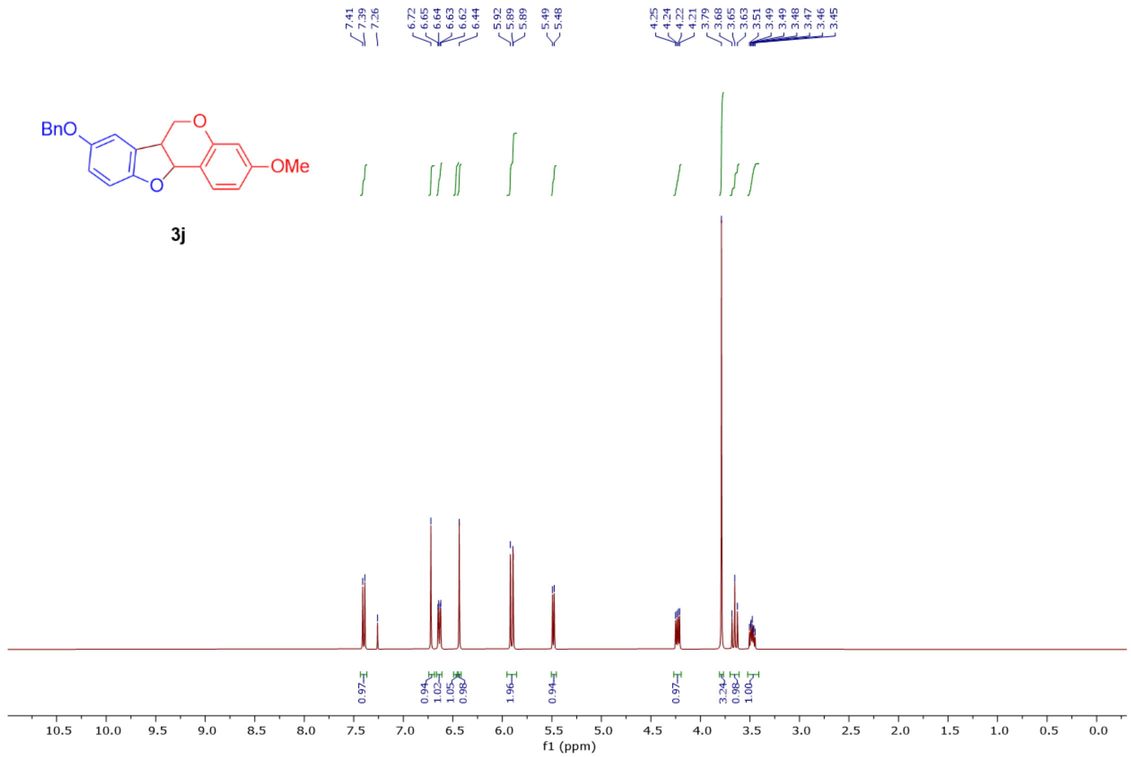

Figure S7-19: ^{1}H NMR spectrum of **3j** in $CDCl_3$

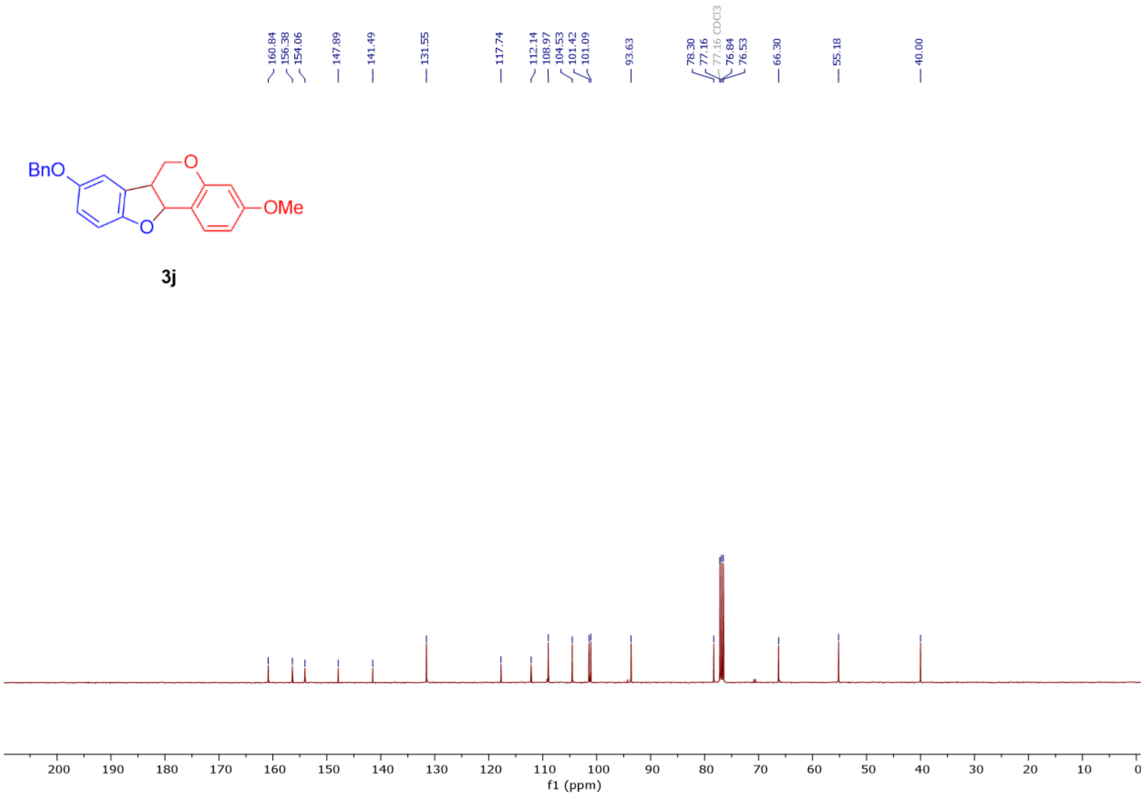

Figure S7-20: ^{13}C NMR spectrum of **3j** in $CDCl_3$

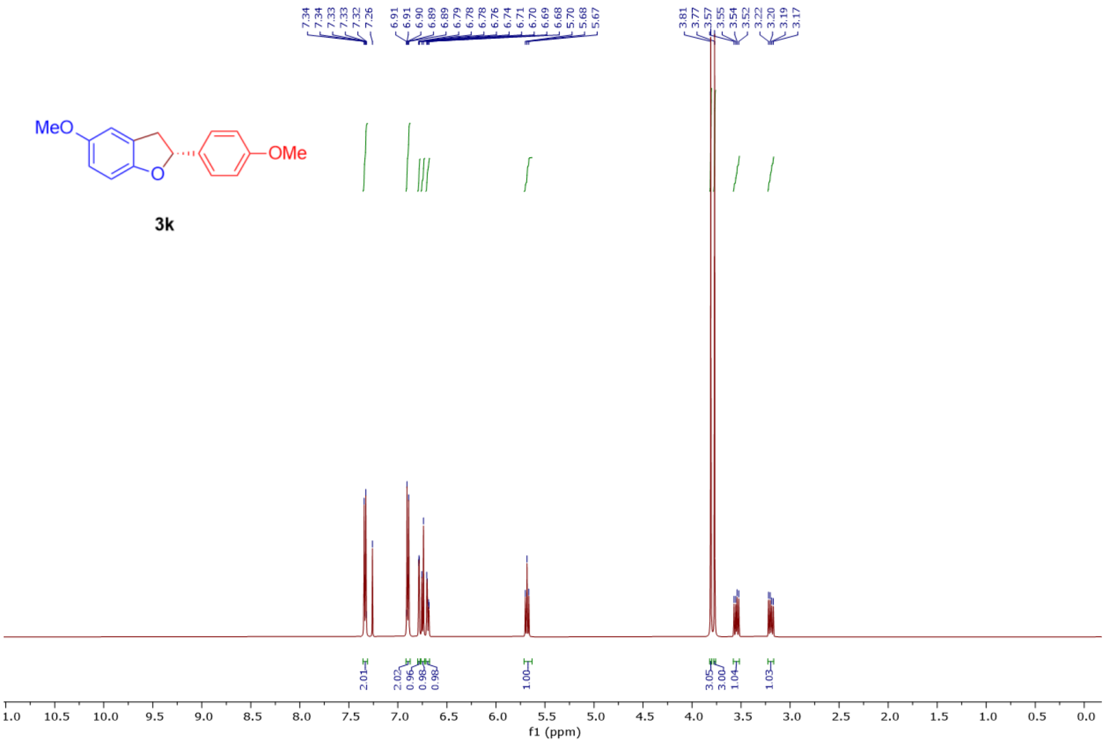

Figure S7-21: ^{1}H NMR spectrum of **3k** in $CDCl_3$

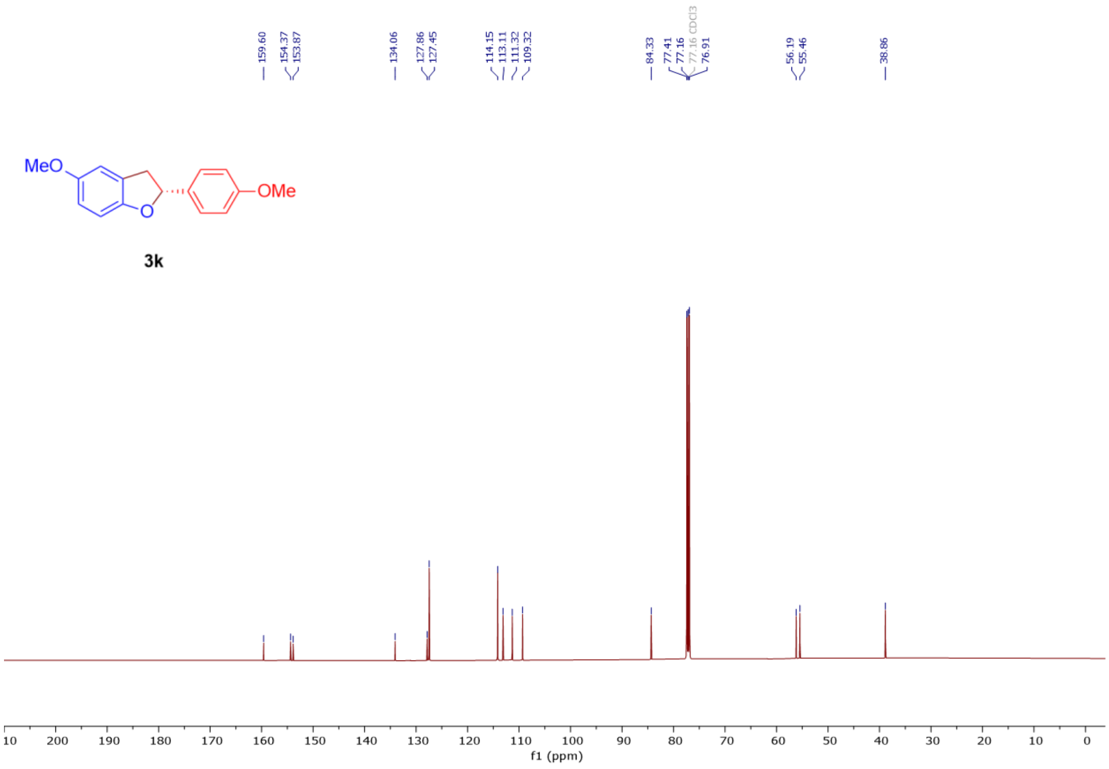

Figure S7-22: ^{13}C NMR spectrum of **3k** in $CDCl_3$

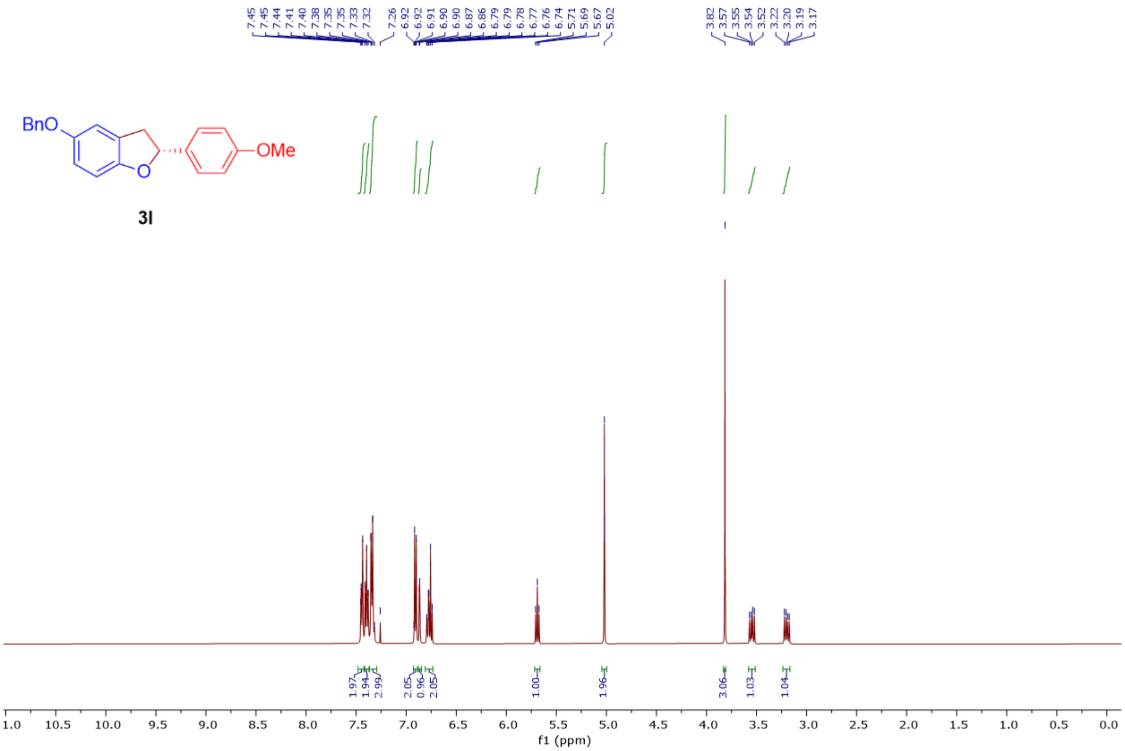

Figure S7-23: ^{1}H NMR spectrum of **3l** in $CDCl_3$

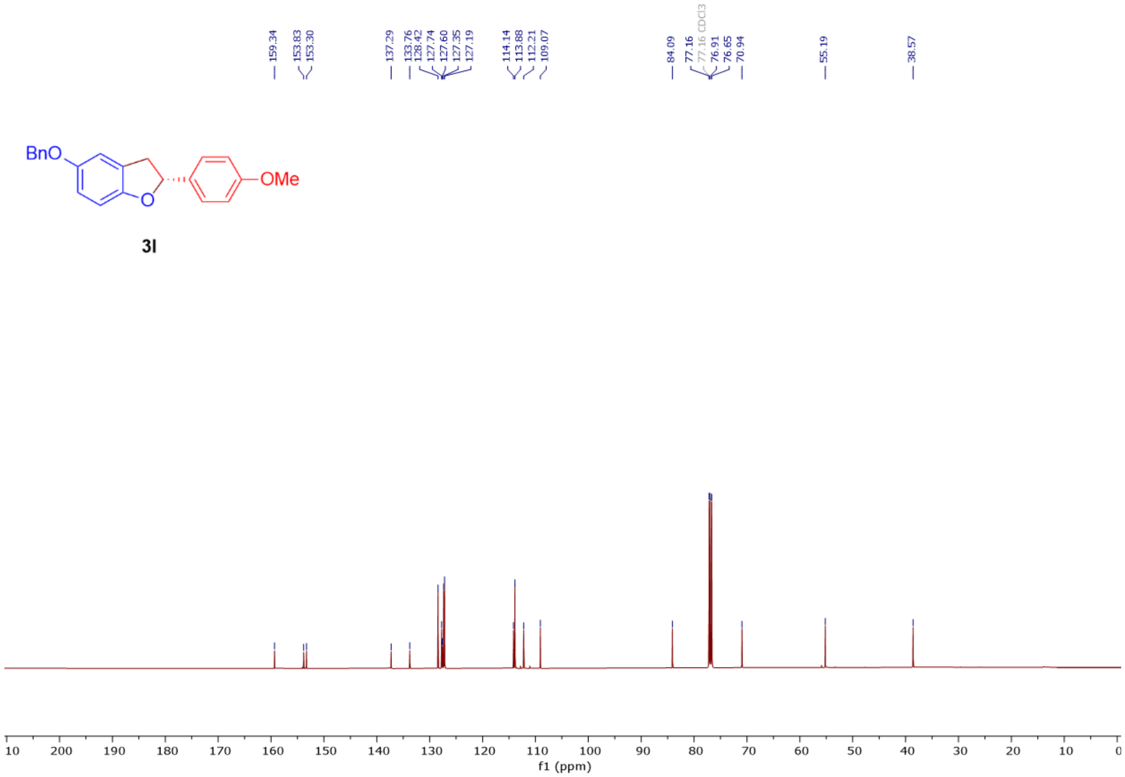

Figure S7-24: ^{13}C NMR spectrum of **3l** in $CDCl_3$

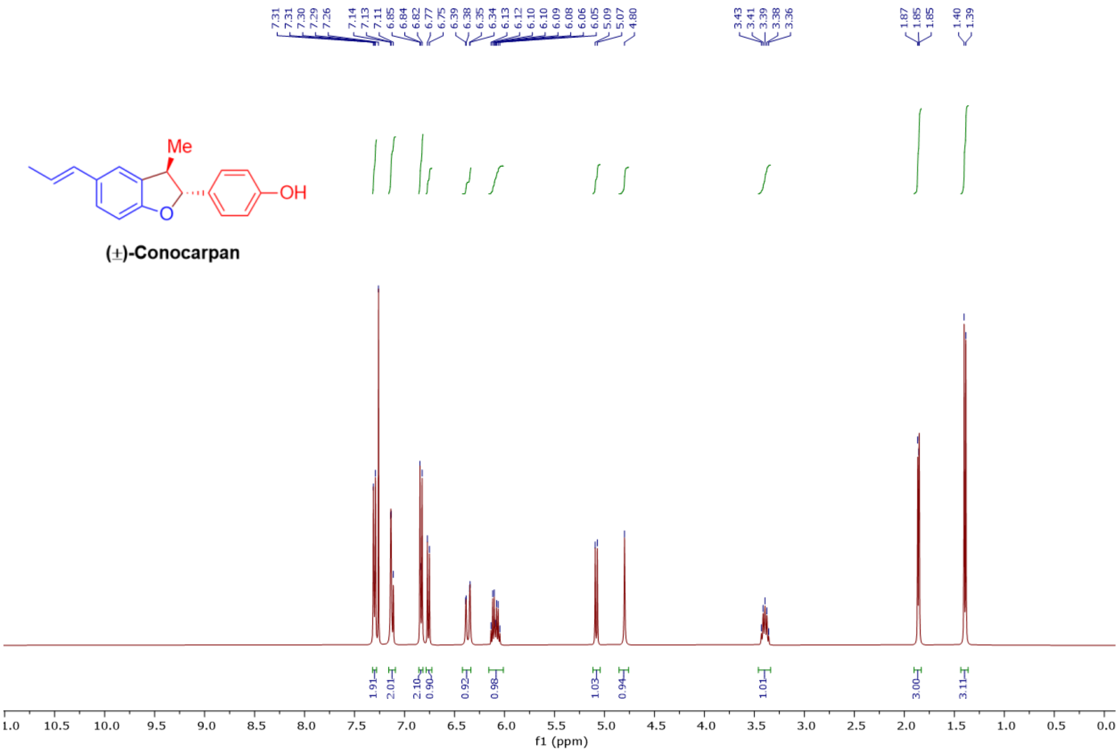

Figure S7-25: ^{1}H NMR spectrum of **(±)-conocarpan** in $CDCl_3$

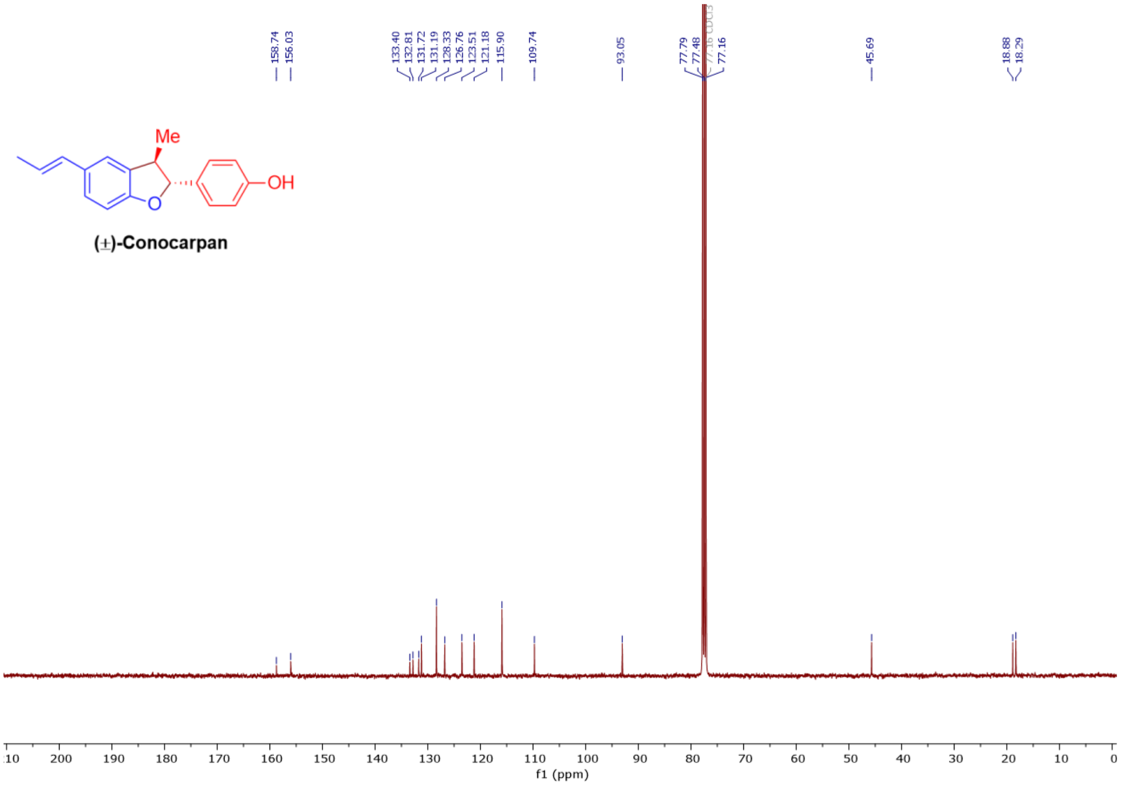

Figure S7-26: ^{13}C NMR spectrum of **(±)-conocarpan** in $CDCl_3$

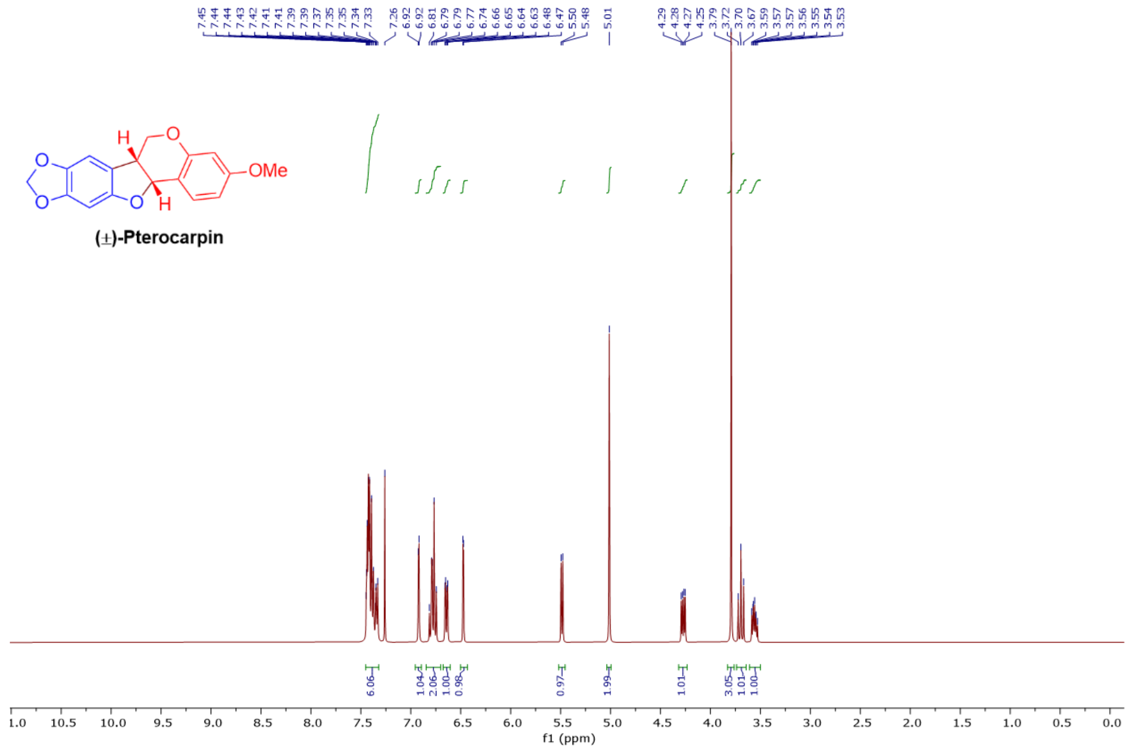

Figure S7-27: ^{1}H NMR spectrum of **(±)-pterocarpin** in $CDCl_3$

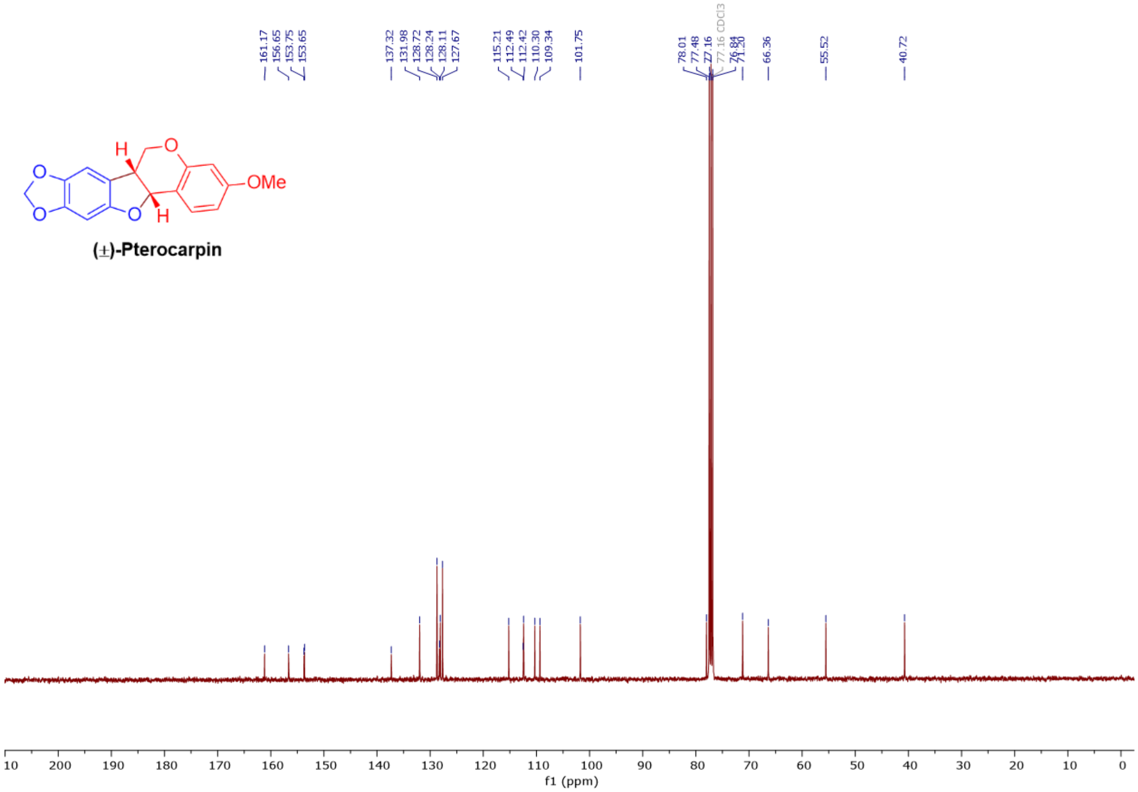

Figure S7-28: ^{13}C NMR spectrum of **(±)-pterocarpin** in $CDCl_3$

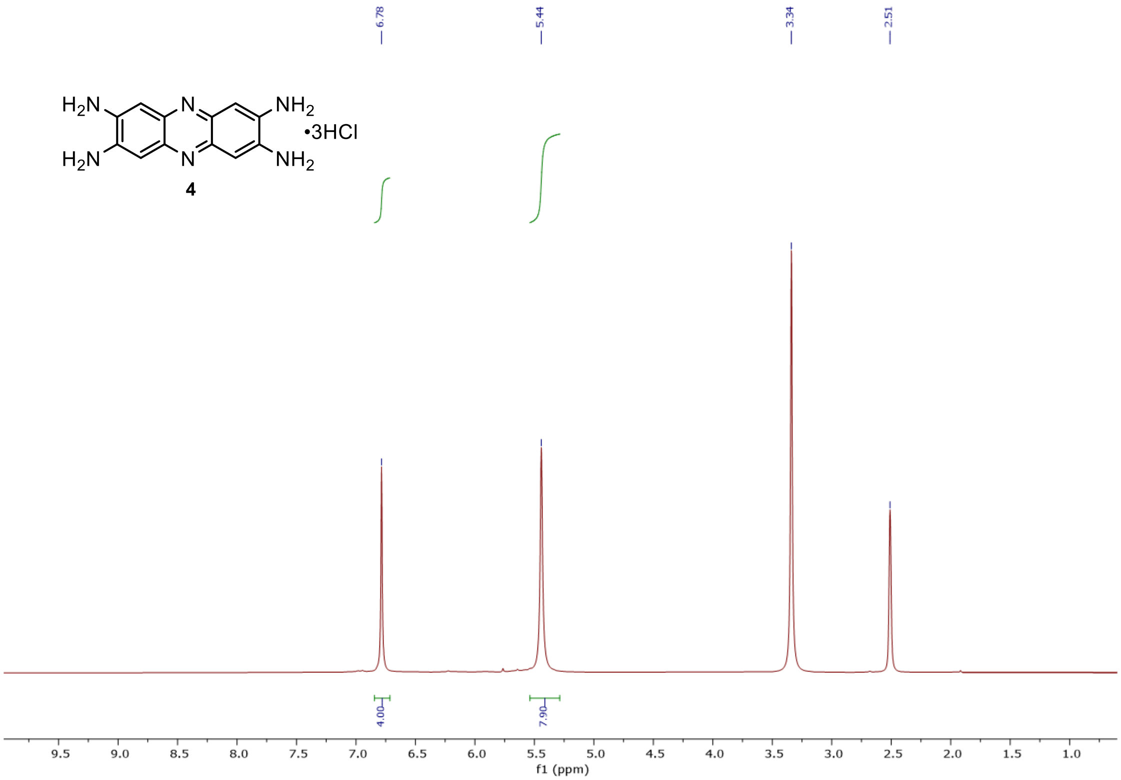

Figure S7-29: ^{1}H NMR spectrum of **4** in DMSO-d6

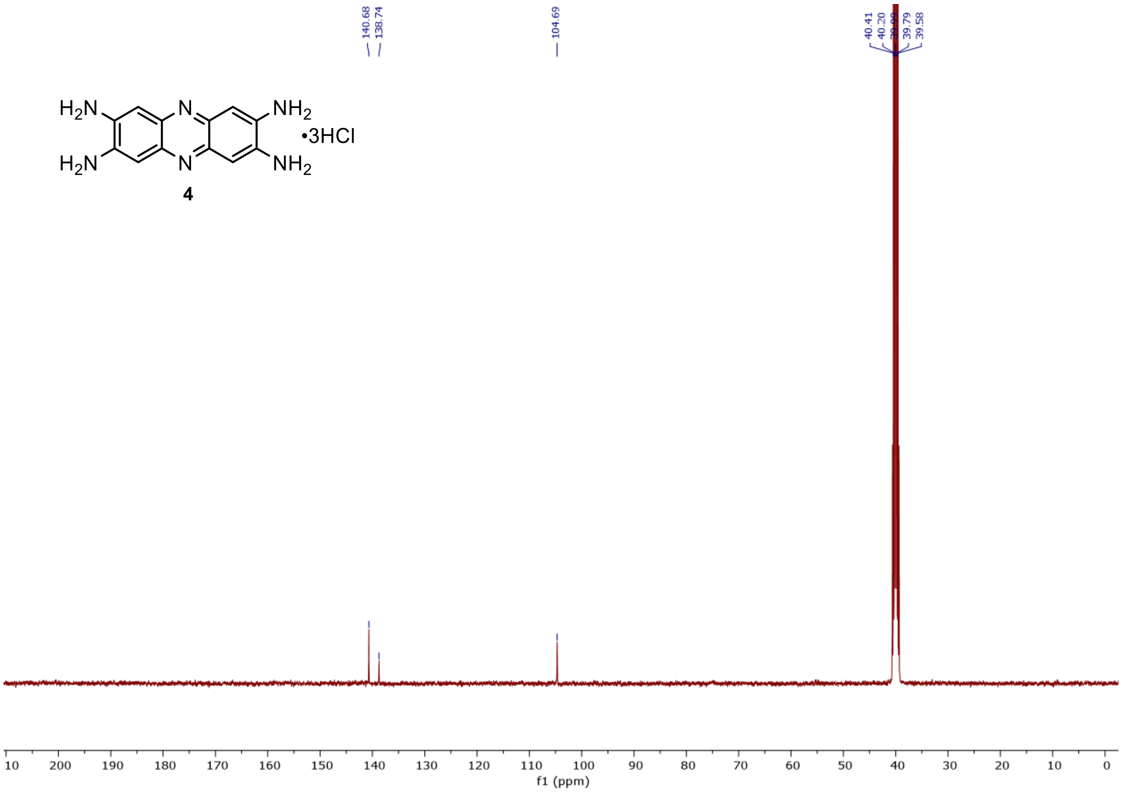

Figure S7-30: ^{13}C NMR spectrum of **4** in DMSO-d6

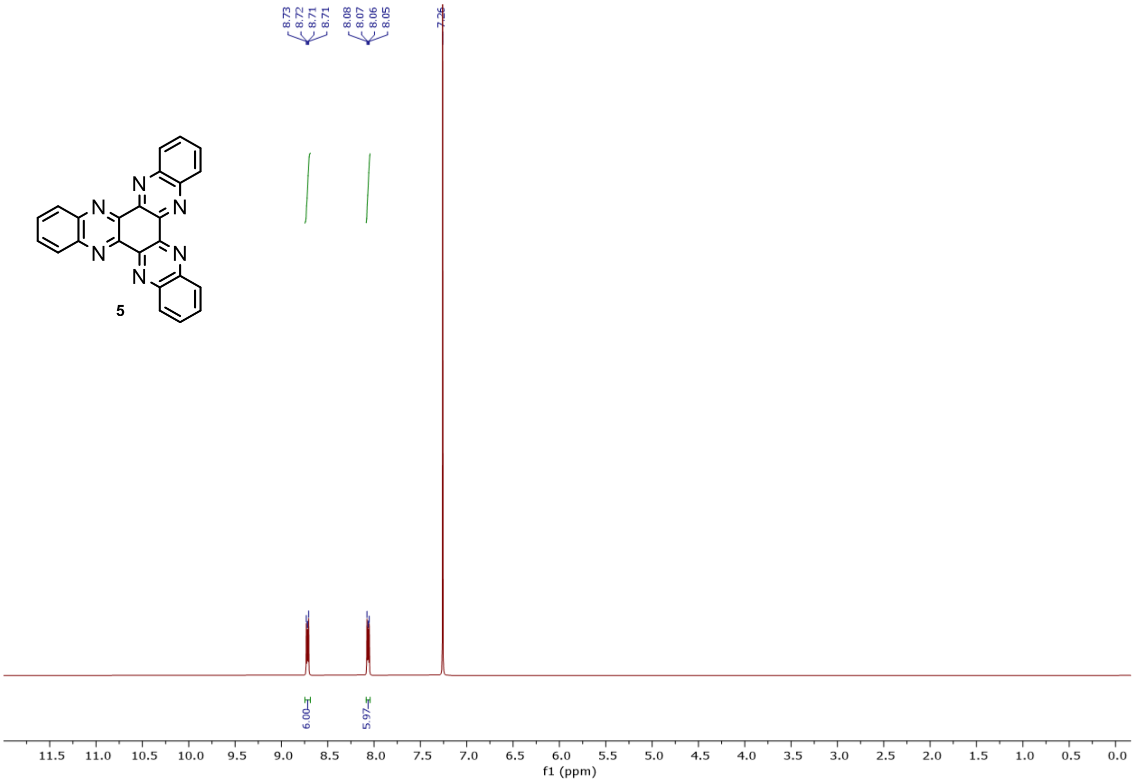

Figure S7-31: ^{1}H NMR spectrum of **5** in $CDCl_3$

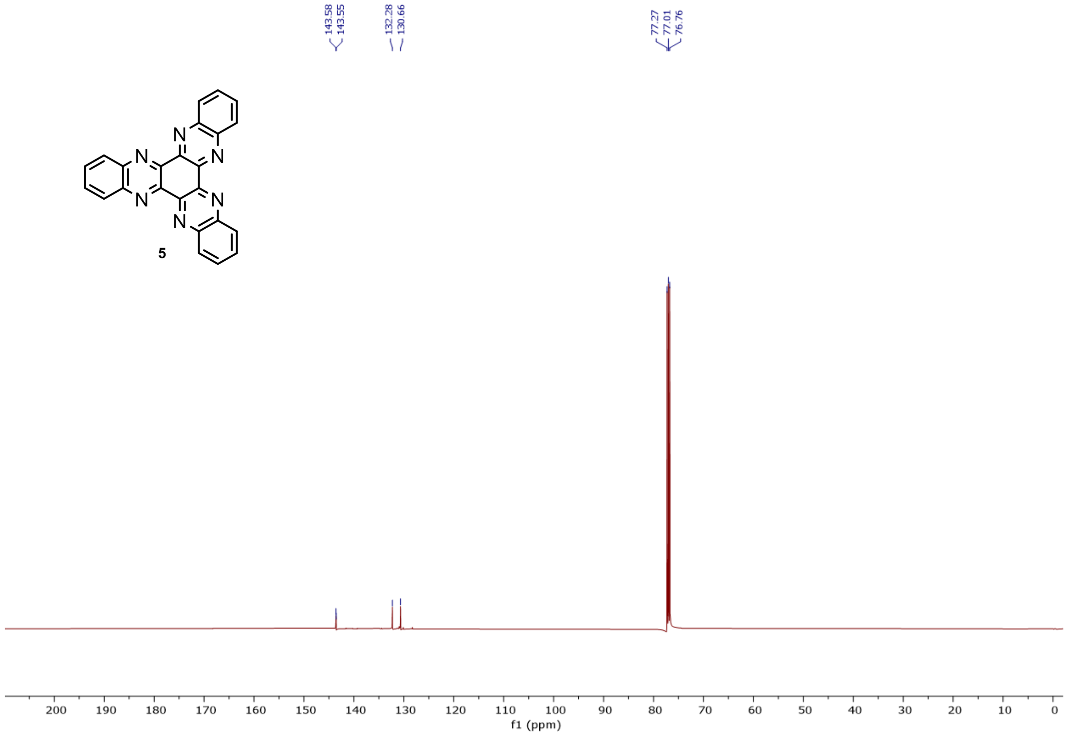

Figure S7-32: ^{13}C NMR spectrum of **5** in $CDCl_3$
